# Effects of non-pharmacological interventions for adults with subjective cognitive decline: a network meta-analysis and component network meta-analysis

**DOI:** 10.1186/s12916-024-03491-z

**Published:** 2024-06-27

**Authors:** Xiao-Hong Yu, Xin-Ru Li, Zhi-Run Du, Yu Zhang, Yang Fei, Wen-Ping Tang, Xian-Wen Li, Qing Zhao

**Affiliations:** 1 School of Nursing, Nanjing Medical University, Nanjing, China; 2 Department of Internal Neurology, The Affiliated Jiangning Hospital of Nanjing Medical University, Nanjing, China; 3 School of Humanities and Health, Changzhou Vocational Institute of Textile and Garment, Changzhou, China; 4 School of Public Health, Southern Medical University, Guangzhou, China; 5 School of Health Service Management, Southern Medical University, Guangzhou, China

**Keywords:** Non-pharmacological interventions, Subjective cognitive decline, Systematic review, Component network meta-analysis

## Abstract

**Background:**

Non-pharmacological interventions have a myriad of available intervention options and contain multiple components. Whether specific components of non-pharmacological interventions or combinations are superior to others remains unclear. The main aim of this study is to compare the effects of different combinations of non-pharmacological interventions and their specific components on health-related outcomes in adults with subjective cognitive decline.

**Methods:**

PubMed, Embase, Cochrane, CINAHL, PsycINFO, CENTRAL, Web of Science, and China’s two largest databases, CNKI and Wanfang, were searched from inception to 22nd, January 2023. Randomized controlled trials using non-pharmacological interventions and reporting health outcomes in adults with subjective cognitive decline were included. Two independent reviewers screened studies, extracted data, and assessed risk of bias. Component network meta-analysis was conducted employing an additive component model for network meta-analysis. This study followed the PRISMA reporting guideline and the PRISMA checklist is presented in Additional file 2.

**Results:**

A total of 39 trials with 2959 patients were included (range of mean ages, 58.79–77.41 years). Resistance exercise might be the optimal intervention for reducing memory complaints in adults with subjective cognitive decline; the surface under the cumulative ranking *p* score was 0.888, followed by balance exercise (*p* = 0.859), aerobic exercise (*p* = 0.832), and cognitive interventions (*p* = 0.618). Music therapy, cognitive training, transcranial direct current stimulation, mindfulness therapy, and balance exercises might be the most effective intervention components for improving global cognitive function (iSMD, 0.83; 95% CI, 0.36 to 1.29), language (iSMD, 0.31; 95% CI, 0.24 to 0.38), ability to perform activities of daily living (iSMD, 0.55; 95% CI, 0.21 to 0.89), physical health (iSMD, 3.29; 95% CI, 2.57 to 4.00), and anxiety relief (iSMD, 0.71; 95% CI, 0.26 to 1.16), respectively.

**Conclusions:**

The form of physical activity performed appears to be more beneficial than cognitive interventions in reducing subjective memory complaints for adults with subjective cognitive decline, and this difference was reflected in resistance, aerobic, and balance exercises. Randomized clinical trials with high-quality and large-scale are warranted to validate the findings.

**Trial registration:**

PROSPERO registry number. CRD42022355363.

**Supplementary Information:**

The online version contains supplementary material available at 10.1186/s12916-024-03491-z.

## Background

Subjective cognitive decline (SCD) refers to an individual’s subjective belief that they have memory or cognitive decline compared to the previous normal state and without objective cognitive impairment [1–3]. The updated Alzheimer’s disease (AD) research framework of the National Institute on Aging and Alzheimer’s Association recognized SCD within the cognitively unimpaired stage on the cognitive continuum [4]. Studies have shown that the annual conversion rate of SCD to mild cognitive impairment (MCI) or AD is approximately 6.67% and that SCD progressively worsens to MCI or AD over nearly 15 years [5]. The risk ratio of developing MCI in SCD is 4.5 and the risk ratio of developing AD is up to 6.5 compared to normal subjects [6]. SCD has become a research hotspot not only because it is a potential early manifestation of AD but also due to that SCD is a broader behavioral phenotype above and beyond preclinical AD [7]. SCD defines a group of people being concerned about their brain health, which reflected in an increasing number of individuals who seek medical advice because of SCD. Identifying effective interventions to address the health needs of elderly individuals with SCD and slow disease progression at an early stage is of utmost importance.

In the absence of specific drugs to treat cognitive-related disorders, non-pharmacological interventions (NPIs) offer an opportunity to delay age-related cognitive decline. Unlike medications that must be prescribed by a physician, NPIs, once standardized, can be implemented by a variety of healthcare professionals with relevant training and expertise (e.g., master’s-level clinicians, occupational therapists, and clinical psychologists). More importantly, the adults with SCD were thought to have preserved current cognitive function and greater cognitive reserve, which increased the likelihood that they would be able to benefit from NPIs before significant cognitive difficulties arose [8].

To our knowledge, there have been some meta-analyses evaluating NPIs for SCD. However, these studies have some areas that need improvement. Firstly, existing systematic reviews tend to focus on objective cognitive abilities as the primary outcome measure [9–11], which is valuable. However, the benefits derived from NPIs may be multi-dimensional. Further examination of differences in the impact of NPIs on different health dimensions of adults with SCD could provide more nuanced insights into the effectiveness of interventions, such as physical health, quality of life, and anxiety. Secondly, most meta-analyses used traditional meta-analysis, which allowed for comparative analyses of the effects of the implementation of the interventions, but it was limited to direct comparisons (within the study) of the evidence provided. Notably, in 2021, Roheger et al. [9] conducted the first network meta-analysis (NMA) to analyze the impact of NPIs on adults with SCD. This analysis allowed for direct comparisons of different interventions by considering both direct and indirect evidence (between-study with shared comparable interventions) in the same model. The results suggest that education programs were most effective for improving memory and cognition. However, the outcome measures in their study were also limited to memory and cognition. Thirdly, the heterogeneity of the study population may affect the generalization of the findings to adults with SCD. For example, a systematic review and meta-analysis by Metternich et al. noted that memory training was the only effective intervention in objective memory [12]. However, the study included a population that involved healthy volunteers who may not necessarily have SCD, which somewhat reduces the informativeness of the findings for the effectiveness of interventions for adults with SCD.

What needs to be emphasized further is that in clinical practice and research literature, NPIs often consist of different combinations of several therapeutic components and some combinations of these components (i.e., some specific forms of NPIs) may have differential effects on interventions for adults with SCD. For example, a web-based in-home multidomain lifestyle intervention may include cognitive training, exercise training, health education, etc. In addition, depending on the intervention’s delivery, setting, and materials, it may also include internetwork, home, and computer components. Consideration of these various components when evaluating the effectiveness and impact of non-pharmacological complex interventions can provide a more comprehensive understanding of the intervention’s potential benefits and provide valuable insights for designing and implementing effective healthcare strategies.

Component network meta-analysis (cNMA) is a newly developed meta-analysis methodology and an extension of the standard NMA that can be used to isolate the treatment effects of different components of a composite intervention [13, 14]. Using cNMA for separating non-pharmacological complex interventions when comparing the treatment effects of NPIs can effectively avoid the influence of heterogeneity of intervention delivery, frequency, setting, and materials across studies on the results and increase the precision of effect estimates between interventions. However, cNMA has not been used to identify non-pharmacological complex interventions in SCD, and the specific combinations of NPI types and the most effective components of complex interventions remain unclear.

Therefore, in this study, standard NMA and cNMA will be applied for the same aimed data. Among them, the standard NMA is mainly used to analyze the effectiveness of different NPIs and their combinations in adults with SCD. It will incorporate more indicators of health outcomes in the analysis to complement the gaps in existing studies. cNMA will be used to determine the incremental effects of the various components of a non-pharmacological complex intervention and to identify the most effective combination of interventions. This will help minimize the complexity of the treatments being offered and may provide an improvement in terms of time, money, and effort for both patients and clinicians.

## Methods

### Search strategy

We systematically searched PubMed, Embase, Cochrane, CINAHL, PsycINFO, CENTRAL, Web of Science, and the two largest databases of CNKI and Wanfang in China. The search period was from the date of database building to January 22, 2023. The search strategy is reported in eAppendix 1 in Additional file 1. We contacted investigators and relevant experimenters by mail to obtain detailed literature information for incomplete reports and information on unpublished experiments. We also conducted searches in reviewed reference lists from included studies and relevant systematic reviews, and performed manual and non-database internet searches to identify gray literature and additional papers that fulfilled the criteria.

### Study selection

We included randomized clinical trials (RCTs) conducted in adults with SCD aged 50 years or older and published in English or Chinese. The definition of the included population is based on the diagnostic criteria of subjective cognitive decline initiative (SCD-I), where SCD is defined as self-reported cognitive complaints, without objective evidence of deficits on cognitive testing and unaccounted for by medical or psychiatric causes [1]. The intervention delivered needed to fall within the domains of NPIs. We excluded (1) studies that included cognitively impaired participants (MCI, AD), psychiatric disease, neurological disease, or substance abuse among the SCD participants and (2) letters, commentaries, method papers, abstracts, and unpublished data. Two assessors independently screened the titles and abstracts and critically reviewed the full texts of the selected studies to assess eligibility.

In this study, subjective memory complaint was defined as the primary outcome because it is not only the most commonly reported area of impairment in adults with SCD but also the core deficit in MCI and AD [15–17]. Secondary outcomes included global cognitive function, language function, executive function, visuospatial ability, attention, and noncognitive functions, including the ability to perform activities of daily living, quality of life, anxiety, depression, and physical health.

### Data extraction and quality assessment

A uniform data extraction form was created that covered the characteristics of the study, demographic details, specific measures of intervention and control, instruments for each outcome measure, and values. For the classification of NPIs, the original table included 11 interventions (cognitive intervention, exercise, aerobic exercise, balance exercise, resistance exercise, psychotherapy, dietary therapy, occupational therapy, usual care, active placebo, and waitlist). However, no study on dietary therapy was included, so this categorization was excluded. Ultimately, the intervention extraction table contained 10 interventions, and the definition of each intervention can be found in Table 1. Data extraction from the included literature was performed independently by two authors (ZRD and XRL). If discrepancies arose, we went through a process of double-checking, group discussions, and consulting the corresponding author (XWL) to make a final decision. The risk of bias for each study was assessed using the Cochrane Evaluation Handbook 5.1.0 [18]. Confidence in Network Meta-Analysis (CINeMA) was used to evaluate confidence in the results from NMA [19].

**Table 1:** List of included interventions, definitions, and their possible components

Interventions	Descriptions	Possible decompositions into components
*Active placebo*	Effect of an intervention due to the patients’ belief that they are receiving some form of treatment	(± fo ± fg ± io ± ig) + (± lf ± mf ± hf) + (± app ± cp ± em ± vr) + (± gym ± hm ± hp ± iom ± lcf)
*Cognitive intervention*	Interventions to improve cognitive functioning, which divided into cognitive training, cognitive stimulation, and cognitive rehabilitation [20]	(± cr ± cs ± ct) + (± fo ± fg ± io ± ig) + (± lf ± mf ± hf) + (± app ± cp ± em ± vr) + (± gym ± hm ± hp ± iom ± lcf)
*Exercise (aerobic group)*	Activity in which the body’s large muscles move in a rhythmic manner for a sustained period of time. Aerobic activity—also called endurance activity—improves cardiorespiratory fitness. Examples include walking, running, swimming, and bicycling [21]	(± ael ± aem ± aev) ± (± fo ± fg ± io ± ig) + (± lf ± mf ± hf) + (± app ± cp ± em ± vr) + (± gym ± hm ± hp ± iom ± lcf)
*Exercise (balance group)*	Static and dynamic exercises that are designed to improve an individual’s ability to withstand challenges from postural sway or destabilizing stimuli caused by self-motion, the environment, or other objects [21]	bat + (± fo ± fg ± io ± ig) + (± lf ± mf ± hf) + (± app ± cp ± em ± vr) + (± gym ± hm ± hp ± iom ± lcf)
*Exercise (resistance group)*	A specialized form of muscle strengthening activity designed to enhance muscular strength, local muscular endurance, and muscular power [22]	ret + (± fo ± fg ± io ± ig) + (± lf ± mf ± hf) + (± app ± cp ± em ± vr) + (± gym ± hm ± hp ± iom ± lcf)
*Exercise*	Physically demanding activities. When the inclusion of literature does not specify the type of exercise or is difficult to categorize as aerobic group, balance group, and resistance group will be referred to as exercise	(± ael ± aem ± aev ± bat ± hitt ± ret) + (± fo ± fg ± io ± ig) + (± lf ± mf ± hf) + (± app ± cp ± em ± vr) + (± gym ± hm ± hp ± iom ± lcf)
*Occupational therapy*	Case management or activities to enhance functional independence, delivered by an occupational therapist, which in this case primarily includes transcranial direct current stimulation [23]	tdcs + fo + (± lf ± mf ± hf) + em + (± gym ± hm ± hp ± iom ± lcf)
*Psychotherapy*	Apply psychological theories, methods, and techniques to change or influence the patient’s negative cognitive emotions	(± cbt ± mft ± mtt) + (± fo ± fg ± io ± ig) + (± lf ± mf ± hf) + (± app ± cp ± em ± vr) + (± gym ± hm ± hp ± iom ± lcf)
*Usual care*	Maintenance of interventions at normality with no additional interventions	hlkp + (± fo ± fg ± io ± ig) + (± lf ± mf ± hf) + (± app ± cp ± em ± vr) + (± gym ± hm ± hp ± iom ± lcf)
*Wl*	Participants are aware that they will receive an active treatment after a waiting phase or be a blank control group	wl

*ael* aerobic exercise and low intensity, *aem* aerobic exercise and moderate intensity, *aev* aerobic exercise and vigorous intensity, *bat* balance training, *cbt* cognitive behavior therapy, *cr* cognitive rehabilitation, *cs* cognitive stimulation, *ct* cognitive training, *hitt* high-intensity interval training, *hlkp* healthy living knowledge promotion, *mft* mindfulness therapy, *mtt* music therapy, *ret* resistance training, *tdcs* transcranial direct current stimulation, *hf* high frequency, *lf* low frequency, *mf* medium frequency, *app* application, *cp* computer, *em* educational material, *vr* virtual reality, *fg* face-to-face and groups, *fo* face-to-face and one-on-one, *ig* internetwork and groups, *io* internetwork and one-on-one, *gy* gym, *hm* home, *hp* hospital, *iom* institute of medicine, *lcf* long-term care facilities, *wl*, waitlist

Symbols: “ + ” means “and”; “ ± ” means “with or without”

### Statistical analysis

Pairwise meta-analysis and standard NMA were performed to compare the comparative effectiveness of different treatments or combinations of NPIs through a frequentist approach based on electrical networks and graph theory [24]. Component network meta-analysis was conducted employing an additive component model for network meta-analysis. We evaluated possible heterogeneity of treatment effects and the robustness of our findings through subgroup analyses. In these analyses, we employed the median duration of treatment as covariates for the primary outcome, ensuring a comprehensive examination of treatment effects across various subgroups. The leave-one-out method and exclusion of high risk of bias studies were employed to conduct sensitivity analysis. Minimally contextualized framework was applied to rank interventions for each intervention outcome, with waitlist as the reference intervention and 95% intervals excluding zero values as the decision threshold [25]. We utilized both the forest plot and the surface under the cumulative ranking (SUCRA) score to compute the relative ranking probabilities for all NPIs concerning research outcomes [26]. Transitivity was assessed by investigating the distribution of potential effect modifiers, including age, gender, and treatment duration. We used the node-splitting approach to estimate whether an inconsistency existed between the direct and indirect evidence. The *Q* statistic was used to assess the fit of the models. A comparison adjusted funnel plot was used to assess small study effects and publication bias. Data analysis was performed using the netmeta package in R (version 4.3.0).

## Results

### Study selection and characteristics

A total of 12,845 publications were searched. After removal of duplicates and screening based on title and abstract, 161 references were retrieved for a full inspection. Finally, 39 RCTs were identified. The selection process and list of the included studies can be seen in eAppendix 2 and eAppendix 3 in Additional file 1, respectively. The 39 studies were published between 1999 and 2022, covering 16 countries, and a total of 2959 adults with SCD were included in the quantitative study. Participants were mostly community-based and predominantly female. The duration of the intervention ranged from 10 days to 26 weeks. These results are described in eAppendix 4 in Additional file 1. The transitivity assessment showed that exercise (aerobic group) + exercise (resistance group) had a higher treatment duration than the other intervention subgroups, but otherwise the other moderators appeared to be evenly distributed across the subgroups (eAppendix 10 in Additional file 1).

### Risk of *bias*

The concordance of the two assessors’ risk of bias was 82.48% in 39 RCTs, and then a third assessor (XWL) was included. Finally, 1 study did not use blinding of participants, personnel, and outcome assessment, 5 studies did not use blinding of participants and personnel, and 1 study did not use blinding for outcome assessment, and these were rated as having a high risk of bias in the corresponding entries. The specific evaluation results can be found in eAppendix 5 in Additional file 1.

### Pairwise *meta*-analysis

#### Primary outcome: subjective memory complaints

We compared the effectiveness of NPIs with usual care, active placebo, and waitlist groups on subjective memory complaints. The results showed that NPIs might be more effective in reducing subjective memory complaints in adults with SCD than usual care (SMD, − 0.29; 95% CI, − 0.48 to − 0.09) and waitlist (SMD, − 0.16; 95% CI, − 0.30 to − 0.01). A comparison of forest plots can be seen in eAppendix 6 in Additional file 1.

#### Secondary outcome

Due to the limitation of the number of active placebo interventions contained in the included studies, the secondary outcome could not be analyzed in a pairwise meta-analysis with active placebo. Therefore, in this section, the comparative reference group for pairwise meta-analysis included the usual care and waitlist group. Among them, the data on quality of life, language function, executive function, visuospatial ability, and depression were insufficient to make comparisons with the usual care and activities of daily living and quality of life data were insufficient to make comparisons with the waitlist. The remaining results show that NPIs had a statistically significant effect on improvements in global cognitive function (SMD, 0.50; 95% CI, 0.09 to 0.91), activities of daily living (SMD, 0.51; 95% CI, 0.34 to 0.67), and physical health (SMD, 0.46; 95% CI, 0.09 to 0.83), when compared with usual care. NPIs may improve subjective memory complaints (SMD, − 0.16; 95% CI, − 0.30 to − 0.01), executive functions (SMD, 0.28; 95% CI, 0.10 to 0.46), and anxiety (SMD, 0.23; 95% CI, 0.02 to 0.43), when compared with the waitlist. The results of all analyzable pairwise meta-analyses are provided in eAppendix 6 in Additional file 1.

### Network *meta*-analysis

#### Primary outcome: subjective memory complaints

**Fig. 1:**
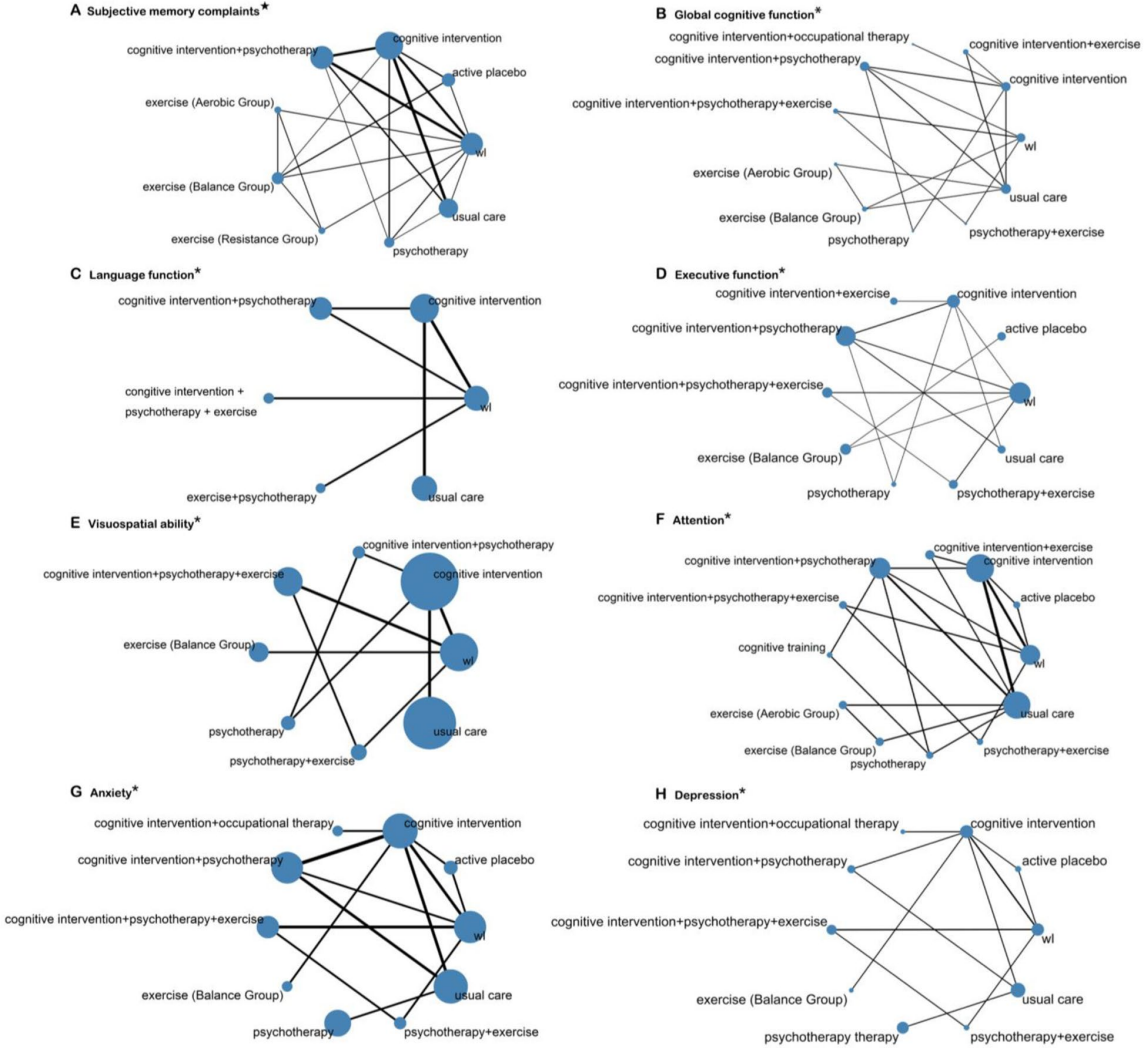
Geometry of networks for comparison of interventions. ^★^Primary outcomes; *secondary outcomes; wl, waitlist. The size of the circles denotes the contribution of participants in each intervention and the thickness of the lines between circles represents the contribution of studies comparing the two interventions

Of the 39 trials, the primary outcome of subjective memory complaints was reported in 21 trials representing 1105 participants, comparing cognitive intervention, cognitive intervention + psychotherapy, exercise (aerobic group), exercise (balance group), exercise (resistance group), psychotherapy, usual care, active placebo, and waitlist (Fig. 1). Cognitive interventions, resistance exercise, aerobic exercise, and balance exercise groups might be more efficacious than usual care and waitlist in reducing subjective memory complaints (Table 2), with SMD ranging from − 0.18 to − 0.78. Resistance exercise received the highest ranking in the minimally contextualized framework and the highest probability (*p* = 0.888) in the SUCRA score. This was followed in order by balance exercise (*p* = 0.859), aerobic exercise (*p* = 0.832), and cognitive interventions (*p* = 0.618). Minimally contextualized framework results and SUCRA ranking can be found in eAppendix 9 in Additional file 1.

**Table 2:** NMA of subjective memory complaint comparisons, iSMD (95% CI)

Active placebo	0.15 (− 0.27 to 0.57)	NA	NA	0.45 (− 0.04 to 0.94)	NA	NA	NA	0.09 (− 0.77 to 0.59)
0.12 (− 0.23 to 0.47)	Cognitive intervention	− **0.27 (**− **0.53 to** − **0.01)**	NA	0.74 (− 0.13 to 1.61)	NA	− **0.85 (**− **1.32 to** − **0.39)**	− **0.39 (**− **0.62 to** − **0.16)**	− **0.32 (**− **0.58 to** − **0.06)**
− 0.19 (− 0.59 to 0.20)	− **0.31 (**− **0.53 to** − **0.10)**	Cognitive intervention + psychotherapy	NA	NA	NA	0.22 (− 0.65 to 1.10)	− 0.11 (− 0.53 to 0.31)	− 0.03 (− 0.28 to 0.22)
0.50 (− 0.14 to 1.14)	0.38 (− 0.23 to 0.99)	**0.69 (0.07 to 1.31)**	Exercise (aerobic group)	− 0.02 (− 0.66 to 0.61)	0.09 (− 0.54 to 0.73)	NA	NA	− 0.64 (− 1.28 to 0.00)
**0.51 (0.12 to 0.91)**	0.39 (− 0.02 to 0.81)	**0.71 (0.27 to 1.15)**	0.01 (− 0.57 to 0.60)	Exercise (balance group)	0.12 (− 0.52 to 0.75)	NA	NA	− 0.62 (− 1.26 to 0.02)
0.59 (− 0.05 to 1.24)	0.47 (− 0.14 to 1.09)	**0.79 (0.17 to 1.41)**	0.10 (− 0.54 to 0.73)	0.08 (− 0.51 to 0.67)	Exercise (resistance group)	NA	NA	− **0.74 (**− **1.38 to** − **0.09)**
− **0.65 (**− **1.17 to** − **0.13)**	− **0.77 (**− **1.16 to** − **0.37)**	− **0.45 (**− **0.87 to** − **0.04)**	− **1.15 (**− **1.86 to** − **0.44)**	− **1.16 (**− **1.72 to** − **0.60)**	− **1.24 (**− **1.95 to** − **0.53)**	Psychotherapy	0.13 (− 0.94 to 1.19)	**0.76 (0.18 to 1.35)**
− 0.28 (− 0.69 to 0.13)	− **0.40 (**− **0.62 to** − **0.18)**	− 0.08 (− 0.35 to 0.19)	− **0.78 (**− **1.42 to** − **0.14)**	− **0.79 (**− **1.25 to** − **0.33)**	− **0.87 (**− **1.51 to** − **0.23)**	0.37 (− 0.06 to 0.80)	Usual care	0.00 (− 0.73 to 0.73)
− 0.18 (− 0.56 to 0.19)	− **0.40 (**− **0.62 to** − **0.18)**	0.01 (− 0.20 to 0.23)	− **0.68 (**− **1.27 to** − **0.09)**	− **0.70 (**− **1.11 to** − **0.28)**	− **0.78 (**− **1.37 to** − **0.18)**	**0.47 (0.06 to 0.88)**	0.10 (− 0.18 to 0.38)	Wl

Results of direct comparisons were listed in the upper triangle, and the estimation was calculated as the standardized mean differences for treatment in the row vs treatment in the column. Results of NMA were listed in the lower triangle, and the estimation was calculated as the standardized mean differences for treatment in the column vs treatment in the row. Statistically significant effects are shown in bold black. Clinically desirable outcome is a decrease ( −)

*iSMD* incremental standardized mean differences, *CI* Confidential intervals, *NA* Not available

#### Secondary outcome

NMA was also performed for all secondary outcomes, and outcomes of global cognitive function, language function, executive function, visuospatial ability, attention, anxiety, and depression with good reticulation were specifically analyzed (Fig. 1). For improving global cognitive functioning, cognitive intervention + psychotherapy might be more effective than usual care (SMD, 0.90; 95% CI, 0.16 to 1.65). For improving executive function, cognitive intervention, cognitive intervention + psychotherapy, cognitive intervention + psychotherapy + exercise, and psychotherapy + exercise might be more effective compared to the usual care, and the SMDs ranged from 0.58 to 1.21. Psychotherapy + exercise might be the most effective measure for improving executive function when compared to waitlist (*p* = 0.942). The results of the network meta-analysis for each of the secondary outcomes are provided in eAppendix 8 in Additional file 1, and the *p* score treatment rankings are provided in eAppendix 7 in Additional file 1.

#### Inconsistency test and certainty of evidence

For all the outcome metrics that form mesh evidence, we did not detect significant inconsistencies, indicating good internal consistency within the network (eAppendix 11 in Additional file 1). The certainty of the evidence was low overall. Concerning the primary and secondary outcomes, we judged the confidence of the evidence to be very low for 34.72% versus waitlist, with 64.89% being low (eAppendix 12 in Additional file 1). Comparison-adjusted funnel plot and Egger’s test did not reveal large asymmetries, suggesting that publication bias and small-study effects may have had a small impact on this study (eAppendix 12 in Additional file 1). No significant heterogeneity was found for the sensitivity analyses that were performed (eAppendix 13 in Additional file 1). Subgroup analyses grouped by median treatment duration showed that the longer treatment duration group (≥ 8 weeks) was more effective in improving subjective memory complaints in adults with SCD than the shorter treatment duration group (< 8 weeks). The results of the subgroup analysis are summarized in eAppendix 14 in Additional file 1.

### Component network *meta*-analysis

According to current research, this study disaggregated the NPIs into 30 components by intervention, delivery, setting, material, and frequency. Specifically, this includes 14 intervention components, 4 delivery and material components each, 5 settings, and 3 frequency components. The components and their definitions are shown in Table 3. The possible components of each intervention are shown in Table 1. The fit of the cNMA model is indicated by *Q* = 224.5, df = 8; for the corresponding NMA model using the same dataset, the fit is represented by *Q* = 20.1, df = 19.

**Table 3:** List of included components and their definitions

	**Component**	**Descriptions**
*Interventions*		
* Cognitive rehabilitation*	***cr***	Physicians and caregivers collaborate to use individualized interventions or strategies to help maintain and improve certain daily living abilities or social functions, such as eating, taking medications, and washing
* Cognitive training*	***ct***	Enhancing cognitive function and increasing cognitive reserve through training in different cognitive domains and cognitive processing
* Cognitive stimulation*	***cs***	Non-specific cognitive interventions in the form of team activities or discussions to improve overall cognitive functioning or social functioning
* Aerobic exercise; low intensity*	***ael***	< 45% VO_2_max or < 50% HRR or < 65% HRmax
* Aerobic exercise; moderate intensity*	***aem***	45–65% VO_2_max or > 50–65% HRR or 65–75% HRmax
* Aerobic exercise; vigorous intensity*	***aev***	> 65% VO_2_max or > 65% HRR or > 75% HRmax
* Balance training*	***bat***	Same as Table 1 definitions
* High-intensity interval training*	***hitt***	A sport that involves repeated bouts of intense exercise (e.g., ≥ 80% of peak or maximum heart rate) separated by intervals of recovery or rest [27]
* Resistance training*	***ret***	Same as Table 1 definitions [22]
* Healthy living knowledge promotion*	***hlkp***	Promote understanding about how behavior impact health, and require individuals to have the capacity to acquire, understand, and operationalize the content of health education in order to improve their health status [28]
* Transcranial direct current stimulation*	***tdcs***	One particular technique, transcranial direct current stimulation, involves the application of low amplitude (e.g., 1–2 mA), sustained current over a short duration (e.g., 20 min) via strategically positioned electrodes on the scalp [29]
* Cognitive behavior therapy*	***cbt***	A therapy in which cognitive restructuring was one of the core components. The aim is evaluating, challenging, and modifying a patient’s dysfunctional beliefs [30]
* Mindfulness therapy*	***mft***	The awareness that emerges through paying attention on purpose, in the present moment, and nonjudgmentally to the unfolding of experience moment by moment [31]
* Music therapy*	***mtt***	The professional use of music and its elements as an intervention in medical, educational, and everyday environment with individuals, groups, families, or communities, who seek to optimize their quality of life and improve their physical, social, communicative, emotional, intellectual, and spiritual health and well-being [32]
*Delivery*		
* Face-to-face and one-on-one*	***fo***	Face-to-face and one-on-one between intervention implementer and study participant when implementing intervention
* Face to face and groups*	***fg***	Face-to-face and groups between intervention implementer and study participant when implementing intervention
* Internetwork and one-on-one*	***io***	One-on-one delivery of interventions through the internet
* Internetwork and groups*	***ig***	Group delivery of interventions through the internet
*Setting*		
* Gym*	***gy***	Indoor places to play sports
* Home*	***hm***	Individual residences of research subjects
* Hospital*	***hp***	Various medical institutions for the purpose of saving lives and treating diseases
* Institute of medicine*	***iom***	Organizations with a mission to advise the nation on matters of health and medicine
* Long-term care facilities*	***lcf***	Public or private long-term care facilities
*Materials*		
* Educational material*	***em***	With the help of paper-based educational materials such as pamphlets and books
* Application*	***app***	Intervention with application software
* Computer*	***cp***	Intervention with computer
* Virtual reality*	***vr***	A technique that utilizes computers to generate a virtual world that directly imposes visual, auditory, and tactile sensations on participants and allows them to interactively observe and manipulate it
*Frequency*		
* Low frequency*	***lf***	Less than 2 times a week on average
* Medium frequency*	***mf***	Average 2 ~ 3 times a week
* High frequency*	***hf***	Greater than 3 times a week on average

*VO*_*2*_*max* maximal oxygen consumption, *HRR* Heart rate reserve, *HRmax* Max heart rate

In terms of the frequency of interventions, medium frequency was able to obtain more benefits compared to low and high frequency. It was effective in improving activities of daily living (iSMD, 0.20; 95% CI, 0.16 to 0.24), quality of life (iSMD, 0.12; 95% CI, 0.08 to 0.17), and physical health (iSMD, 0.46; 95% CI, 0.12 to 0.81). For the delivery of NPIs, face-to-face communication provides more benefits for language function and visuospatial abilities than adopting the internet. In interventions, music therapy, cognitive training, transcranial direct current stimulation, and balance exercises were the most effective intervention components for improving global cognitive function (iSMD, 0.83; 95% CI, 0.36 to 1.29), language functioning (iSMD, 0.31; 95% CI, 0.24 to 0.38), ability to perform activities of daily living (iSMD, 0.55; 95% CI, 0.21 to 0.89), and anxiety relief (iSMD, 0.71; 95% CI, 0.26 to 1.16), respectively. Mindfulness therapy was the most effective component in improving quality of life and physical health in adults with SCD, but it was also the least effective component in reducing subjective memory complaints (iSMD, 0.89; 95% CI, 0.02 to 1.76). From the material, the most effective intervention materials for global cognitive function, quality of life, and physical health were educational material (iSMD, 0.73; 95% CI, 0.30 to 1.16), computers (iSMD, 0.30; 95% CI, 0.25 to 0.36), and applications (iSMD, 0.98; 95% CI, 0.51 to 1.45), respectively. In terms of setting, home was shown to be unfavorable for language functioning (iSMD, − 0.34; 95% CI, − 0.50 to − 0.18) and visuospatial ability (iSMD, − 0.14; 95% CI, − 0.24 to − 0.03), but favorable for quality of life (iSMD, 0.27; 95% CI, 0.21 to 0.33). Hospital was unfavorable for visuospatial ability (iSMD, − 0.12; 95% CI, − 0.22 to − 0.01) and quality of life (iSMD, − 0.14; 95% CI, − 0.14 to − 0.09). Estimates of the incremental standardized mean difference of each component are shown in Table 4.

**Table 4:** Estimates of the incremental standardized mean difference of each component when added to the waitlist

Component		Subjective memory complaints^a^	Global cognitive function^b^	Language function^b^	Executive function^b^	Visuospatial ability^b^	Attention^b^	Activities of daily living^b^	Quality of life^b^	Anxiety^b^	Depression^b^	Physical health^b^
		**iSMD (95% CI)**	**iSMD (95% CI)**	**iSMD (95% CI)**	**iSMD (95% CI)**	**iSMD (95% CI)**	**iSMD (95% CI)**	**iSMD (95% CI)**	**iSMD (95% CI)**	**iSMD (95% CI)**	**iSMD (95% CI)**	**iSMD (95% CI)**
***Delivery***	***fg***	0.04 [− 0.85 to 0.94]	− 0.36 [− 0.77 to 0.05]	**0.26[0.14 to 0.37]**	− 0.10 [− 0.80 to 0.60]	**0.39[0.19 to 0.58]**	**0.66[0.04 to 1.27]**	**0.78[0.27 to 1.29]**	− **0.18[**− **0.24 to** − **0.11]**	− 0.09 [− 0.29 to 0.11]	0.33 [− 0.08 to 0.74]	− 0.14 [− 0.73 to 0.44]
***Delivery***	***fo***	− 0.11 [− 0.80 to 0.59]	− **0.93[**− **1.62 to** − **0.24]**	**0.26[0.14 to 0.37]**	0.43 [− 0.85 to 1.71]	**0.48[0.20 to 0.77]**	− 0.22 [− 0.79 to 0.36]	− 0.05 [− 0.13 to 0.03]	**0.27[0.21 to 0.33]**	− **0.66[**− **1.02 to** − **0.29]**	0.04 [− 0.46 to 0.53]	− **0.25[**− **0.46 to** − **0.04]**
***Delivery***	***ig***	− 0.06 [− 0.87 to 0.74]	0.11 [− 0.23 to 0.44]	− **0.07[**− **0.13 to** − **0.01]**	0.14 [− 2.13 to 2.42]	NA	0.23 [− 0.31 to 0.76]	NA	NA	NA	NA	NA
***Delivery***	***io***	− 0.24 [− 0.82 to 0.34]	0.54 [− 0.20 to 1.29]	− 0.13 [− 0.28 to 0.01]	0.14 [− 1.72 to 2.00]	− **0.81[**− **1.30 to** − **0.31]**	− 0.16 [− 1.00 to 0.67]	0.51 [− 0.03 to 1.04]	**0.60[0.41 to 0.79]**	**0.52[0.15 to 0.90]**	0.12 [− 0.47 to 0.72]	0.02 [− 0.62 to 0.66]
***Frequency***	***hf***	− 0.18 [− 0.65 to 0.30]	NA	− 0.06 [− 0.26 to 0.13]	− 0.04 [− 1.02 to 0.95]	0.06 [− 0.37 to 0.49]	0.04 [− 0.27 to 0.34]	NA	NA	− 0.15 [− 0.35 to 0.05]	0.05 [− 0.26 to 0.36]	NA
***Frequency***	***lf***	0.28 [− 0.63 to 1.19]	− 0.28 [− 0.58 to 0.01]	− **0.14[**− **0.21 to** − **0.08]**	− 0.27 [− 1.86 to 1.33]	− 0.14 [− 0.53 to 0.24]	− 0.25 [− 0.82 to 0.31]	− 0.05 [− 0.13 to 0.03]	NA	0.09 [− 0.11 to 0.28]	0.30 [− 0.10 to 0.69]	− **0.45[**− **0.82 to** − **0.09]**
***Frequency***	***mf***	− 0.22 [− 1.26 to 0.82]	− 0.01 [− 0.38 to 0.37]	− **0.45[**− **0.58 to** − **0.31]**	0.31 [− 0.69 to 1.31]	− 0.09 [− 0.21 to 0.02]	− 0.81 [− 1.63 to 0.01]	**0.20[0.16 to 0.24]**	**0.12[0.08 to 0.17]**	− 0.18 [− 0.36 to 0.01]	0.04 [− 0.27 to 0.34]	**0.46[0.12 to 0.81]**
***Interventions***	***ael***	− 0.52 [− 1.88 to 0.85]	− 0.12 [− 0.40 to 0.17]	NA	0.13 [− 0.85 to 1.11]	− **0.12[**− **0.22 to** − **0.01]**	0.05 [− 0.44 to 0.55]	**0.10[0.04 to 0.16]**	− 0.14 [− 0.20 to − 0.09]	**0.53[0.22 to 0.85]**	0.12 [− 0.14 to 0.39]	− 0.11 [− 0.40 to 0.18]
***Interventions***	***aem***	− 0.14 [− 0.71 to 0.42]	0.35 [0.00 to 0.70]	**0.11[0.03 to 0.18]**	0.32 [− 0.41 to 1.06]	− 0.12 [− 0.23 to 0.00]	0.28 [− 0.21 to 0.76]	**0.05[0.02 to 0.09]**	NA	− 0.06 [− 0.37 to 0.25]	− 0.17 [− 0.56 to 0.21]	− **0.53[**− **0.79 to** − **0.26]**
***Interventions***	***aev***	− 0.18 [− 0.82 to 0.46]	− 0.18 [− 0.60 to 0.24]	**0.11[0.03 to 0.18]**	− 0.41 [− 1.47 to 0.64]	− **0.14[**− **0.24 to** − **0.03]**	NA	**0.05[0.02 to 0.09]**	NA	− 0.06 [− 0.37 to 0.25]	− 0.17 [− 0.56 to 0.21]	− **0.25[**− **0.46 to** − **0.04]**
***Interventions***	***bat***	− 0.30 [− 0.79 to 0.19]	NA	NA	NA	NA	NA	NA	NA	**0.71[0.26 to 1.16]**	0.08 [− 0.39 to 0.56]	0.29 [− 0.12 to 0.71]
***Interventions***	***cbt***	0.77 [− 0.50 to 2.04]	NA	NA	0.28 [− 0.58 to 1.14]	NA	0.08 [− 0.44 to 0.59]	NA	NA	NA	NA	− 0.07 [− 0.39 to 0.24]
***Interventions***	***cr***	NA	NA	NA	0.28 [− 0.58 to 1.14]	NA	NA	NA	NA	NA	NA	− 0.07 [− 0.39 to 0.24]
***Interventions***	***cs***	− 0.41 [− 1.22 to 0.40]	**0.65[0.48 to 0.82]**	**0.11[0.03 to 0.18]**	− **0.19[**− **1.72 to 1.34]**	− **0.14[**− **0.24 to** − **0.03]**	0.39 [− 0.02 to 0.79]	− 0.05 [− 0.13 to 0.03]	**0.58[0.49 to 0.66]**	**0.59[0.02 to 1.16]**	− 0.26 [− 0.54 to 0.02]	− **0.25[**− **0.46 to** − **0.04]**
***Interventions***	***ct***	0.03 [− 0.40 to 0.47]	− 0.08 [− 0.36 to 0.20]	**0.31[0.24 to 0.38]**	− 0.06 [− 1.16 to 1.04]	0.39 [0.00 to 0.78]	0.06 [− 0.22 to 0.33]	NA	**0.16[0.10 to 0.22]**	− 0.01 [− 0.27 to 0.25]	− 0.20 [− 0.68 to 0.27]	0.08 [− 0.43 to 0.59]
***Interventions***	***hitt***	NA	NA	NA	NA	NA	NA	NA	NA	NA	NA	− **0.40[**− **0.79 to** − **0.01]**
***Interventions***	***hlkp***	0.27 [− 0.16 to 0.70]	− 0.27 [− 0.77 to 0.23]	**0.26[0.20 to 0.32]**	− 0.12 [− 1.22 to 0.98]	0.23 [− 0.16 to 0.61]	− 0.06 [− 0.48 to 0.37]	− **0.20[**− **0.24 to** − **0.16]**	− 0.02 [− 0.15 to 0.12]	0.02 [− 0.17 to 0.21]	0.24 [− 0.12 to 0.61]	− 0.10 [− 0.40 to 0.20]
***Interventions***	***mft***	**0.89[0.02 to 1.76]**	**0.65[0.48 to 0.82]**	0.00 [− 0.13 to 0.14]	0.26 [− 1.14 to 1.66]	− **0.14[**− **0.24 to** − **0.03]**	0.06 [− 0.30 to 0.41]	− 0.05 [− 0.13 to 0.03]	**0.88[0.76 to 1.00]**	0.29 [− 0.14 to 0.72]	0.04 [− 0.36 to 0.44]	**3.29[2.57 to 4.00]**
***Interventions***	***mtt***	NA	**0.83[0.36 to 1.29]**	NA	0.67 [− 0.30 to 1.63]	NA	0.13 [− 0.36 to 0.63]	NA	**0.88[0.76 to 1.00]**	NA	NA	NA
***Interventions***	***ret***	− 1.18 [− 3.04 to 0.67]	− **0.42[**− **0.60 to** − **0.23]**	NA	NA	NA	NA	− 0.01 [− 0.07 to 0.06]	NA	NA	NA	− 0.02 [− 0.37 to 0.33]
***Interventions***	***tdcs***	NA	0.21 [− 0.25 to 0.68]	NA	NA	NA	NA	**0.55[0.21 to 0.89]**	NA	0.32 [− 0.09 to 0.72]	0.14 [− 0.51 to 0.78]	NA
***Materials***	***app***	− 0.18 [− 0.65 to 0.30]	**0.42[0.23 to 0.60]**	− 0.06 [− 0.26 to 0.13]	− 0.04 [− 1.02 to 0.95]	0.06 [− 0.37 to 0.49]	0.04 [− 0.27 to 0.34]	NA	NA	− 0.15 [− 0.35 to 0.05]	0.05 [− 0.26 to 0.36]	**0.98[0.51 to 1.45]**
***Materials***	***cp***	− 0.22 [− 1.04 to 0.60]	0.11 [− 0.23 to 0.44]	− **0.07[**− **0.13 to** − **0.01]**	0.34 [− 2.78 to 3.47]	NA	0.23 [− 0.31 to 0.76]	NA	**0.30[0.25 to 0.36]**	0.54 [− 0.04 to 1.12]	NA	NA
***Materials***	***em***	− 0.41 [− 0.93 to 0.12]	**0.73[0.30 to 1.16]**	0.00 [− 0.08 to 0.08]	0.23 [− 2.28 to 2.74]	− 0.06 [− 0.45 to 0.33]	0.18 [− 0.35 to 0.70]	− 0.05 [− 0.13 to 0.03]	**0.12[0.08 to 0.17]**	0.22 [− 0.14 to 0.58]	− 0.14 [− 0.44 to 0.15]	− 0.06 [− 0.67 to 0.55]
***Materials***	***vr***	NA	NA	NA	NA	NA	0.26 [− 0.46 to 0.97]	NA	NA	NA	NA	NA
***Setting***	***gym***	0.60 [− 1.86 to 3.06]	NA	NA	NA	NA	NA	NA	NA	NA	NA	NA
***Setting***	***hm***	0.45 [− 0.66 to 1.56]	− 0.18 [− 0.60 to 0.24]	− **0.34[**− **0.50 to** − **0.18]**	0.12 [− 0.50 to 0.73]	− **0.14[**− **0.24 to** − **0.03]**	0.04 [− 0.27 to 0.34]	− 0.05 [− 0.13 to 0.03]	**0.27[0.21 to 0.33]**	− 0.21 [− 0.45 to 0.03]	− 0.12 [− 0.41 to 0.16]	− 0.18 [− 0.45 to 0.10]
***Setting***	***hp***	NA	− 0.12 [− 0.40 to 0.17]	NA	0.13 [− 0.85 to 1.11]	− **0.12[**− **0.22 to** − **0.01]**	0.05 [− 0.44 to 0.55]	NA	− **0.14[**− **0.20 to** − **0.09]**	− 0.18 [− 0.36 to 0.01]	0.04 [− 0.27 to 0.34]	− 0.11 [− 0.40 to 0.18]
***Setting***	***iom***	NA	− 0.10 [− 0.52 to 0.33]	NA	− 0.38 [− 1.31 to 0.55]	0.02 [− 0.08 to 0.12]	NA	NA	NA	NA	NA	0.29 [− 0.13 to 0.71]
***Setting***	***lcf***	NA	NA	NA	NA	NA	NA	NA	NA	NA	NA	NA

*iSMD* incremental standardized mean differences, *CI* Confidential intervals, *ael* aerobic exercise and low intensity, *aem* aerobic exercise and moderate intensity, *aev* aerobic exercise and vigorous intensity, *bat* balance training, *cbt* cognitive behavior therapy, *cr* cognitive rehabilitation, *cs* cognitive stimulation, *ct* cognitive training, *hitt* high-intensity interval training, *hlkp* healthy living knowledge promotion, *mft* mindfulness therapy, *mtt* music therapy, *ret* resistance training, *tdcs* transcranial direct current stimulation, *hf* high frequency, *lf* low frequency, *mf* medium frequency, *app* application, *cp* computer, *em* educational material, *vr* virtual reality, *fg* face-to-face and groups, *fo* face-to-face and one-on-one, *ig* internetwork and groups, *io* internetwork and one-on-one, *gy* gym, *hm* home, *hp* hospital, *iom* institute of medicine, *lcf* long-term care facilities. Statistically significant effects are shown in bold black

primary outcomes: clinically desirable outcome is a decrease ( −); secondary outcomes: clinically desirable outcome is an increased ( +); *NA*, not available

^a^Primary outcomes

^b^secondary outcomes

## Discussion

To our knowledge, this systematic review and network meta-analysis is the most comprehensive synthesis of data on NPIs for adults with SCD, and it is also the first study using cNMA to explore the incremental effects of the components of a non-pharmacological complex intervention. By considering both direct and indirect comparative evidence, this study found that resistance, aerobic, and balance exercises were all superior to cognitive interventions in reducing subjective memory complaints in adults with SCD. In other words, for adults with SCD, the form of physical activity performed appears to be more beneficial in reducing subjective memory complaints. Among the forms of physical activity interventions, resistance exercise might be most effective in reducing subjective memory complaints in adults with SCD. This effect may be related to the fact that resistance exercise can increase blood flow to memory-related brain regions [33], produce neuroprotective factors [34], and achieve greater neuronal survival and synaptogenesis [35]. These findings suggest that subjective memory complaints can be reduced by identifying adults with SCD in community and primary care settings, adopting tailored exercise programs and appropriately adding a resistance exercise portion.

Furthermore, it has been hypothesized that the combination of two or more NPIs could potentially decelerate cognitive decline at a more substantial rate compared to the use of a single NPI alone [36]. However, we hold the perspective that this concept is not absolute, but rather reflects a dialectical viewpoint. For instance, in our study, the utilization of cognitive interventions alone yielded a significant improvement in subjective memory complaints among adults with SCD, whereas a combination of cognitive intervention and psychotherapy did not produce a significant effect. The notion that multiple combined NPIs do not consistently outperform single intervention is also evident in the outcomes related to executive function in the findings of Metternich et al. [12]. Accordingly, the implementation of NPIs should not be exclusively centered on quantity and complexity. Instead, it should emphasize the interplay of synergistic and antagonistic effects among various interventions.

NPIs encompass a multitude of components, even in their simplest forms. Regarding the subjective memory outcome measure, the most effective effect sizes were observed for the components io (internet and one-on-one), mf (medium frequency), ael (aerobic exercise; low intensity), cs (cognitive stimulation), and em (educational material). This implies that future NPIs for SCD could incorporate these components more extensively. It is important to note, however, that these effect sizes did not reach statistical significance, so the conclusions should be treated with caution. Similarly, in the context of global cognitive function, the components mft (mindfulness therapy), mtt (music therapy), app (application), and em (educational material) exhibited more favorable effect sizes, and these effect sizes were statistically significant. Consequently, when using NPIs to improve global cognitive functioning in adults with SCD, we suggest that future trials incorporate these components more broadly and incorporate longer follow-up periods to gain insight into the crucial role of duration in both the effectiveness and sustainability of interventions. In addition, we did not extract components for treatment duration and not included them in the cNMA. This is because 33 (84.6%) of the 39 studies in the literature included in this study had treatment durations centered on 1 to 3 months, with only 5 (12.8%) being greater than 3 months and 1 (2.6%) being less than 1 month. However, the transitivity analysis results suggested that the intervention duration was higher in the exercise (aerobic group) + exercise (resistance group) group than in the other intervention subgroups, which may influence the effect size of indirect evidence to be biased. On the other hand, the results of the subgroup analyses based on the median treatment duration show that appropriately increasing the duration of NPIs may be more conducive to the improvement of subjective memory complaint in adults with SCD, which also suggests differences in the impact of heterogeneity in treatment duration on health outcomes in adults with SCD. Therefore, we suggest that future studies use cNMA to explore further the differences in the efficacy of NPIs by treatment duration, such as short-term, medium-term, and long-term interventions, when sufficient data supports them. It will help to improve the statistical power of indirect comparisons and determine the incremental effects of different treatment durations.

It is worth emphasizing that we utilized the *Q* statistic to assess the fit of the comparative standard NMA and cNMA models, as they were used on the same dataset. In fact, with the widespread use of traditional NMA models, several organizations, such as the WHO, have endorsed NMA as a powerful tool in clinical decision-making because it has developed greater maturity and robustness. The results of the goodness-of-fit test of the NMA model in this study also increase the confidence in promoting and applying NPIs in adults with SCD. However, while NMA can improve the precision of estimates most of the time by combining not only direct but also indirect evidence, it cannot further assess the impact of components in non-pharmacological complex interventions. At this point, as an extension of the standard NMA, the cNMA can provide information on component effects by informing all studies and the results with intervention effects that show a monotonic pattern between effect and components. Therefore, NMA and cNMA should not be considered competitors but complementary. Although the inclusion of more classifications in the cNMA analysis may have affected the fit of their models, we still believe this article provides valuable insights for discerning the most effective components among NPIs for SCD. This, in turn, contributes to future research focusing on intervention components that are deemed effective. As far as we know, contemporary studies are adopting efficient research designs, such as multiphase optimization strategies to assess intervention components [37].

This study also has some other limitations. Firstly, the extensive number of studies included in the analysis and the intricate statistical analyses required a substantial amount of time to complete. Consequently, studies published in recent months could not be included in time. Secondly, the majority of the included studies exhibited small sample sizes, which compromises the reliability of the estimated results. Thirdly, despite our efforts to establish standardized training and evaluation criteria, the definition and identification of intervention components inevitably retained a degree of subjectivity. Fourthly, due to a scarcity of direct evidence, a considerable portion of our conclusions rely on indirect comparisons. Consequently, these outcomes should be approached with caution. Lastly, the limited availability of follow-up data hindered our capacity to conduct comprehensive analyses regarding the evolving effects of NPIs and their components on various outcomes over time. Consequently, our assessment of long-term outcomes remains incomplete.

## Conclusions

Our study demonstrates that different forms of physical exercise, including resistance, aerobic, and balance exercises, might outperform cognitive interventions in improving subjective memory complaints in adults with SCD. For delaying cognitive decline in adults with SCD, combined interventions of two or more NPIs are not always superior to one non-pharmacological intervention alone. The combination of multiple NPIs should focus more on synergistic and antagonistic effects among various interventions. We hope that these findings and the preliminary recommendations provided can inform clinical interventions and encourage the field to further explore optimal component combination strategies to maximize the efficacy of non-pharmacological interventions and influence or alter the trajectory of abnormal cognitive decline.

### Supplementary Information

Additional file 1.

Additional file 2. PRISMA 2020 checklist.

## Data Availability

All data analyzed in this study are available in this published article and supplementary material. The references of articles included in this network meta-analysis are presented on the reference list and the background data of the original studies in the supplementary material.

## Abbreviations

AD: Alzheimer’s disease
CI: Confidential intervals
CINeMA: Confidence in Network Meta-Analysis
cNMA: Component network meta-analysis
iSMD: Incremental standardized mean differences
MCI: Mild cognitive impairment
NMA: Network meta-analysis
NPIs: Non-pharmacological interventions
RCTs: Randomized clinical trials
SCD: Subjective cognitive decline
SMDs: Standardized mean differences
SUCRA: Surface under the cumulative ranking
