# Supplementary material for: Effects of non-pharmacological interventions for adults with subjective cognitive decline: a network meta-analysis and component network meta-analysis
Source: BMC Med. 2024 Jun 27;22:272. doi: 10.1186/s12916-024-03491-z (PMC11209990; doi:10.1186/s12916-024-03491-z)
Supplement: Supplementary file 1 — Additional file 1. [file 12916_2024_3491_MOESM1_ESM.docx]

**Supplementary**

[eAppendix 1. Full Search Strategy 2](#_Toc158624711)

[eAppendix 2. PRISMA flow diagram of the screened studies 7](#_Toc158624712)

[eAppendix 3. List of the included studies 8](#_Toc158624713)

[eAppendix 4. Study Characteristics 13](#_Toc158624714)

[eAppendix 5. Bias Assessment of Eligible Studies 19](#_Toc158624715)

[eAppendix 6. Pairwise Meta-analysis of forest plots 21](#_Toc158624716)

[eAppendix 7. Network Meta-analysis of forest plots and p-scores 25](#_Toc158624717)

[eAppendix 8. Comparison of network meta-analysis, iSMD (95%CI) 27](#_Toc158624718)

[eAppendix 9. Results of minimally contextualised framework 32](#_Toc158624719)

[eAppendix 10. Assessment of transitivity: distribution of effect modifiers 38](#_Toc158624720)

[eAppendix 11. Inconsistency test for direct and indirect evidence 40](#_Toc158624721)

[eAppendix 12. Grading the evidence of the network meta-analysis using CINeMA 45](#_Toc158624722)

[12.1 CINeMA quality assessment of the comparisons in the network 45](#_Toc158624723)

[12.2 Results of the comparison-adjusted funnel plot 48](#_Toc158624724)

[12.3 Risk of Bias Chart showing the contribution of low, moderate or high RoB comparisons to each network estimates. 52](#_Toc158624725)

[12.4 Results of the CINeMA quality assessment 59](#_Toc158624726)

[eAppendix 13. Sensitivity analyses 74](#_Toc158624727)

[eAppendix 14. Subgroup analysis 74](#_Toc158624728)

# eAppendix 1. Full Search Strategy

We did not find a description consistent with subjective cognitive decline in the Mesh thesaurus, so we used free-word search.

**1.1 Cochrane Library**

Databases on the Cochrane Library CDSR, CENTRAL, DARE, HTA and NHS-EED have been searched using the following strategy note specific list of subjective memory decline:

#1 (subjective memory complaint*):ti,ab,kw

#2 (subjective memory loss):ti,ab,kw

#3 (subjective memory impairment*):ti,ab,kw

#4 (subjective memory decline*):ti,ab,kw

#5 (cognitive complaint*):ti,ab,kw

#6(memory complaint*):ti,ab,kw

#7 (subjective cognitive complaint*):ti,ab,kw

#8 (subjective cognitive loss):ti,ab,kw

#9 (subjective cognitive impairment*):ti,ab,kw

#10 (subjective cognitive decline*):ti,ab,kw

#11 #1 OR #2 OR #3 OR #4 OR #5 OR #6 OR #7 OR #8 OR #9 OR #10

#12 (randomized controlled trial):ti,ab,kw

#13 (controlled clinical trial):ti,ab,kw

#14 (random*):ti,ab,kw

#15 (alloc*):ti,ab,kw

#16 (assign*):ti,ab,kw

#17 (placebo):ti,ab,kw

#18 #12 OR #13 OR #14 OR #15 OR #16 OR #17

#19 #11 AND #18

**1.2 PubMed**

PubMed have been searched using the following terms:

#1 subjective memory complaint*[Title/Abstract]

#2 subjective memory loss[Title/Abstract]

#3 subjective memory impairment*[Title/Abstract]

#4 subjective memory decline*[Title/Abstract]

#5 cognitive complaint*[Title/Abstract]

#6 memory complaint*[Title/Abstract]

#7 subjective cognitive complaint*[Title/Abstract]

#8 subjective cognitive loss[Title/Abstract]

#9 subjective cognitive impairment*[Title/Abstract]

#10 subjective cognitive decline*[Title/Abstract]

#11 #1 OR #2 OR #3 OR #4 OR #5 OR #6 OR #7 OR #8 OR #9 OR #10

#12 randomized controlled trial[Title/Abstract]

#13 controlled clinical trial[Title/Abstract]

#14 random*[Title/Abstract]

#15 alloc*[Title/Abstract]

#16 assign*[Title/Abstract]

#17 placebo[Title/Abstract]

#18#12 OR #13 OR #14 OR #15 OR #16 OR #17

#19 #11 AND #18

**1.3 EBSCOhost Research Databases**

PsycINFO, PsycARTICLES and CINAHL databases were searched through EBSCOhost Research Databases.

1.3.1 CINAHL

CINAHL have been searched using the following terms:

S1AB subjective memory complaint* OR AB subjective memory loss OR AB subjective memory impairment* OR AB subjective memory decline* OR AB cognitive complaint* OR AB memory complaint* OR AB subjective cognitive complaint* OR AB subjective cognitive loss OR

AB subjective cognitive impairment* OR AB subjective cognitive decline* Expanders - Apply equivalent subjects

*Search modes* - Find all my search terms Interface - EBSCOhost Research Databases

*Search Screen* - Advanced Search

*Database* - CINAHL Plus with Full Text

S2AB randomized controlled trial OR AB controlled clinical trial OR AB random* OR AB alloc* OR AB assign* OR AB placebo Expanders- Apply equivalent subjects

*Search modes* - Find all my search terms Interface- EBSCOhost Research Databases

*Search Screen* - Advanced Search

*Database* - CINAHL Plus with Full Text

S3(AB randomized controlled trial OR AB controlled clinical trial OR AB random* OR AB alloc* OR AB assign* OR AB placebo) AND (S1 AND S2) Expanders - Apply equivalent subjects

*Search modes* - Find all my search terms Interface - EBSCOhost Research Databases

*Search Screen* - Advanced Search

*Database* - CINAHL Plus with Full Text

1.3.2 PsycINFO

We searched PsycINFO using the following terms:

S1 AB subjective memory complaint* OR AB subjective memory loss OR AB subjective memory impairment* OR AB subjective memory decline* OR AB cognitive complaint* OR AB memory complaint* OR AB subjective cognitive complaint* OR AB subjective cognitive loss OR AB subjective cognitive impairment* OR AB subjective cognitive decline*

S2 AB randomized controlled trial OR AB controlled clinical trial OR AB random* OR AB alloc* OR AB assign* OR AB placebo

S3 S1 AND S2

1.3.3 PsycARTICLES

We searched PsycINFO using the following terms:

S1 AB subjective memory complaint* OR AB subjective memory loss OR AB subjective memory impairment* OR AB subjective memory decline* OR AB cognitive complaint* OR AB memory complaint* OR AB subjective cognitive complaint* OR AB subjective cognitive loss OR

AB subjective cognitive impairment* OR AB subjective cognitive decline*

S2 AB randomized controlled trial OR AB controlled clinical trial OR AB random* OR AB alloc* OR AB assign* OR AB placebo

S3 S1 AND S2

**1.4 Web of Science**

#1 (TI=(subjective memory complaint)) OR AB=(subjective memory complaint)

#2 (TI=(subjective memory loss)) OR AB=(subjective memory loss)

#3 (TI=(subjective memory impairment)) OR AB=(subjective memory impairment)

#4 (TI=(subjective memory decline)) OR AB=(subjective memory decline)

#5 (TI=(cognitive complaint)) OR AB=(cognitive complaint)

#6 (TI=(memory complaint)) OR AB=(memory complaint)

#7 (TI=(subjective cognitive complaint)) OR AB=(subjective cognitive complaint)

#8 (TI=(subjective cognitive loss)) OR AB=(subjective cognitive loss)

#9 (TI=(subjective cognitive impairment)) OR AB=(subjective cognitive impairment)

#10 (TI=(subjective cognitive decline)) OR AB=(subjective cognitive decline)

#11 #1 OR #2 OR #3 OR #4 OR #5 OR #6 OR #7 OR #8 OR #9 OR #10

#12 (TI=(randomized controlled trial)) OR AB=(randomized controlled trial)

#13 (TI=(controlled clinical trial)) OR AB=(controlled clinical trial)

#14 (TI=(random*)) OR AB=(random*)

#15 (TI=(alloc*)) OR AB=(alloc*)

#16 (TI=(assign)) OR AB=(assign)

#17 (TI=(placebo)) OR AB=(placebo)

#18 #12 OR #13 OR #14 OR #15 OR #16 OR #17

#19 #11 AND #18

**1.5 Embase**

We searched Embase using the following terms:

#1 'subjective memory complaint*':ab,ti

#2 'subjective memory loss':ab,ti

#3 'subjective memory impairment*':ab,ti

#4 'subjective memory decline*':ab,ti

#5 'cognitive complaint*':ab,ti

#6 'memory complaint*':ab,ti

#7 'subjective cognitive complaint*':ab,ti

#8 'subjective cognitive loss':ab,ti

#9 'subjective cognitive impairment*':ab,ti

#10 'subjective cognitive decline*':ab,ti

#11 #1 OR #2 OR #3 OR #4 OR #5 OR #6 OR #7 OR #8 OR #9 OR #10

#12 'randomized controlled trial':ab,ti

#13 'controlled clinical trial':ab,ti

#14 'random*':ab,ti

#15 'alloc*':ab,ti

#16 'assign*':ab,ti

#17 'placebo':ab,ti

#18 #12 OR #13 OR #14 OR #15 OR #16 OR #17

#19 #11 AND #18

**1.6 Chinese Databases**

1.6.1 China National Knowledge Infrastructure (CNKI)

We performed searches in China National Knowledge Infrastructure (CNKI) using the following Chinese terms:

#1 （篇关摘%主观记忆减退）OR(关键词%主观记忆减退）

#2 （篇关摘%主观记忆障碍）OR(关键词%主观记忆障碍）

#3 （篇关摘%主观记忆损害）OR(关键词%主观记忆损害）

#4 （篇关摘%主观记忆衰退）OR(关键词%主观记忆衰退）

#5 （篇关摘%主观记忆抱怨）OR(关键词%主观记忆抱怨）

#6 （篇关摘%主观记忆下降）OR(关键词%主观记忆下降）

#7 （篇关摘%记忆抱怨主诉）OR(关键词%记忆抱怨主诉）

#8 （篇关摘%主观认知减退）OR(关键词%主观认知减退）

#9 （篇关摘%主观认知障碍）OR(关键词%主观认知障碍）

#10 （篇关摘%主观认知损害）OR(关键词%主观认知损害）

#11 （篇关摘%主观认知衰退）OR(关键词%主观认知衰退）

#12 （篇关摘%主观认知抱怨）OR(关键词%主观认知抱怨）

#13 （篇关摘%主观认知下降）OR(关键词%主观认知下降）

#14 #1 OR #2 OR #3 OR #4 OR #5 OR #6 OR #7 OR #8 OR #9 OR #10 OR #11 OR #12 OR #13

#15 (篇关摘%随机对照试验）OR(关键词%随机对照试验）

#16 (篇关摘%随机对照研究）OR(关键词%随机对照研究）

#17 (篇关摘%临床对照试验）OR(关键词%临床对照试验）

#18 (篇关摘%临床对照研究）OR(关键词%临床对照研究）

#19 (篇关摘%随机）OR(关键词%随机）

#20 (篇关摘%分配）OR(关键词%分配）

#21 (篇关摘%安慰剂）OR(关键词%安慰剂）

#22 #15 OR #16 OR #17 OR #18 OR #19 OR #20 OR #21

#23 #14 * #22

[中国知网 (cnki.net)](https://www.cnki.net/) （Hold down ctrl and click the accessible link）

1.6.2 WanFang Database

We performed searches in WanFang Database using the following Chinese terms:

#1 题名或关键词:(主观记忆减退) or 摘要:(主观记忆减退)

#2 题名或关键词:(主观记忆障碍) or 摘要:(主观记忆障碍)

#3 题名或关键词:(主观记忆损害) or 摘要:(主观记忆损害)

#4 题名或关键词:(主观记忆衰退) or 摘要:(主观记忆衰退)

#5 题名或关键词:(主观记忆抱怨) or 摘要:(主观记忆抱怨)

#6 题名或关键词:(主观记忆下降) or 摘要:(主观记忆下降)

#7 题名或关键词:(记忆抱怨主诉) or 摘要:(记忆抱怨主诉)

#8 题名或关键词:(主观认知减退) or 摘要:(主观认知减退)

#9 题名或关键词:(主观认知障碍) or 摘要:(主观认知障碍)

#10 题名或关键词:(主观认知损害) or 摘要:(主观认知损害)

#11 题名或关键词:(主观认知衰退) or 摘要:(主观认知衰退)

#12 题名或关键词:(主观认知抱怨) or 摘要:(主观认知抱怨)

#13 题名或关键词:(主观认知下降) or 摘要:(主观认知下降)

#14 #1 OR #13

#15 题名或关键词:(随机对照试验) or 摘要:(随机对照试验)

#16 题名或关键词:(随机对照研究) or 摘要:(随机对照研究)

#17 题名或关键词:(临床对照试验) or 摘要:(临床对照试验)

#18 题名或关键词:(临床对照研究) or 摘要:(临床对照研究)

#19 题名或关键词:(随机) or 摘要:(随机)

#20 题名或关键词:(分配) or 摘要:(分配)

#21 题名或关键词:(安慰剂) or 摘要:(安慰剂)

#22 #15 OR #16 OR #17 OR #18 OR #19 OR #20 OR #21

#23 #14 and #22

[万方数据知识服务平台 (wanfangdata.com.cn)](https://www.wanfangdata.com.cn/) （Hold down ctrl and click the accessible link）

# eAppendix 2. PRISMA flow diagram of the screened studies


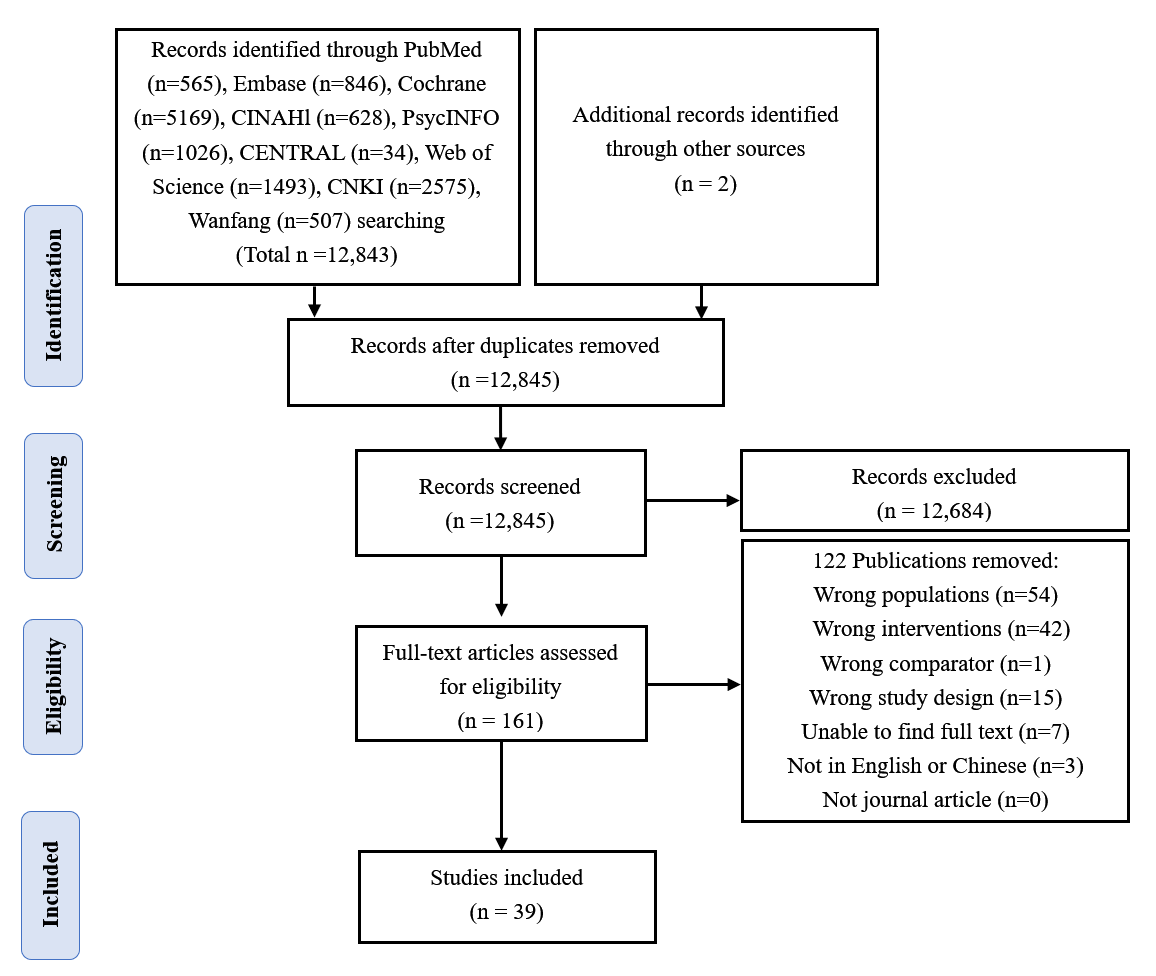


# eAppendix 3. List of the included studies

1. Bozoki A, Radovanovic M, Winn B, Heeter C, Anthony JC. Effects of a computer-based cognitive exercise program on age-related cognitive decline. Arch Gerontol Geriatr. 2013;57(1):1-7.
2. Brooks H, Oughli HA, Kamel L, Subramanian S, Morgan G, Blumberger DM, et al. Enhancing Cognition in Older Persons with Depression or Anxiety with a Combination of Mindfulness-Based Stress Reduction (MBSR) and Transcranial Direct Current Stimulation (tDCS): Results of a Pilot Randomized Clinical Trial. Mindfulness (N Y). 2021;12(12):3047-59.
3. Cabral DF, Santos VS, Pereira OTT, Silva MJ, Pascual-Leone A, Rundek T, et al. Feasibility and Preliminary Efficacy of a Multimodal Approach to Increase Physical Activity in Older Adults With Memory Complaints: The Education for Action Study. J Aging Phys Act. 2022;30(2):204-16.
4. Cavalcante BR, de Souza MF, Falck RS, Liu-Ambrose T, Behm DG, Pitangui ACR, et al. Effects of Resistance Exercise with Instability on Cognitive Function (REI Study): A Proof-Of-Concept Randomized Controlled Trial in Older Adults with Cognitive Complaints. J Alzheimers Dis. 2020;77(1):227-39.
5. Chan AS, Cheung WK, Yeung MK, Woo J, Kwok T, Shum DHK, et al. A Chinese Chan-based Mind-Body Intervention Improves Memory of Older Adults. Front Aging Neurosci. 2017;9:190.
6. Cohen-Mansfield J, Cohen R, Buettner L, Eyal N, Jakobovits H, Rebok G, et al. Interventions for older persons reporting memory difficulties: a randomized controlled pilot study. Int J Geriatr Psychiatry. 2015;30(5):478-86.
7. Cox KL, Clare L, Cyarto EV, Ellis KA, Etherton-Beer C, Southam J, et al. A Randomized Controlled Trial on the Effects of a 6-Month Home-Based Physical Activity Program with Individual Goal-Setting and Volunteer Mentors on Physical Activity, Adherence, and Physical Fitness in Inactive Older Adults at Risk of Cognitive Decline: The INDIGO Study. J Alzheimers Dis. 2021;84(1):207-26.
8. Cuesta M, Verty LV, Abdessalem HB, Byrns A, Bruneau M-A, Frasson C, et al., editors. Virtual Reality and EEG-Based Intelligent Agent in Older Adults With Subjective Cognitive Decline: A Feasibility Study for Effects on Emotion and Cognition. Frontiers in Virtual Reality. 2022;2:807991.
9. Dawson D, Richardson J, Troyer A, Binns M, Clark A, Polatajko H, et al. An occupation-based strategy training approach to managing age-related executive changes: a pilot randomized controlled trial. Clin Rehabil. 2014;28(2):118-27.
10. Denny KG, Chan ML, Gravano J, Harvey D, Meyer OL, Huss O, et al. A randomized control trial of a behavioral intervention for older adults with subjective cognitive complaints that combines cognitive rehabilitation strategies and lifestyle modifications. Neuropsychol Dev Cogn B Aging Neuropsychol Cogn. 2023;30(1):78-93.
11. Fabre C, Massé-Biron J, Chamari K, Varray A, Mucci P, Préfaut C. Evaluation of quality of life in elderly healthy subjects after aerobic and/or mental training. Arch Gerontol Geriatr. 1999;28(1):9-22.
12. Fairchild JK, Scogin FR. Training to Enhance Adult Memory (TEAM): an investigation of the effectiveness of a memory training program with older adults. Aging Ment Health. 2010;14(3):364-73.
13. Frankenmolen NL, Overdorp EJ, Fasotti L, Claassen J, Kessels RPC, Oosterman JM. Memory Strategy Training in Older Adults with Subjective Memory Complaints: A Randomized Controlled Trial. J Int Neuropsychol Soc. 2018;24(10):1110-20.
14. Fujita K, Umegaki H, Makino T, Uemura K, Hayashi T, Inoue A, et al. Short- and long-term effects of different exercise programs on the gait performance of older adults with subjective cognitive decline: A randomized controlled trial. Exp Gerontol. 2021;156:111590.
15. Hermes S, Lurz M, Böhm M, Krcmar H, editors. Evaluating the Usability and Usefulness of a Mobile Application for Training Visual Mnemonic Techniques in Participants with Subjective Cognitive Decline: An Exploratory Pilot Study. EUSPN/ICTH. 2019;160:439-444.
16. Hong YJ, Lee JH, Choi EJ, Han N, Kim JE, Park SH, et al. Efficacies of Cognitive Interventions in the Elderly with Subjective Cognitive Decline: A Prospective, Three-Arm, Controlled Trial. J Clin Neurol. 2020;16(2):304-13.
17. Hoogenhout EM, de Groot RH, van der Elst W, Jolles J. Effects of a comprehensive educational group intervention in older women with cognitive complaints: a randomized controlled trial. Aging Ment Health. 2012;16(2):135-44.
18. Innes KE, Montgomery C, Selfe TK, Wen S, Khalsa DS, Flick M. Incorporating a Usual Care Comparator into a Study of Meditation and Music Listening for Older Adults with Subjective Cognitive Decline: A Randomized Feasibility Trial. J Alzheimers Dis Rep. 2021;5(1):187-206.
19. Iuliano E, Fiorilli G, Aquino G, Di Costanzo A, Calcagno G, di Cagno A. Twelve-Week Exercise Influences Memory Complaint but not Memory Performance in Older Adults: A Randomized Controlled Study. J Aging Phys Act. 2017;25(4):612-20.
20. Krause-Sorio B, Siddarth P, Kilpatrick L, Milillo MM, Aguilar-Faustino Y, Ercoli L, et al. Yoga Prevents Gray Matter Atrophy in Women at Risk for Alzheimer's Disease: A Randomized Controlled Trial. J Alzheimers Dis. 2022;87(2):569-81.
21. Kwok T, Wong A, Chan G, Shiu YY, Lam KC, Young D, et al. Effectiveness of cognitive training for Chinese elderly in Hong Kong. Clin Interv Aging. 2013;8:213-9.
22. Kwok TC, Bai X, Li JC, Ho FK, Lee TM. Effectiveness of cognitive training in Chinese older people with subjective cognitive complaints: a randomized placebo-controlled trial. Int J Geriatr Psychiatry. 2013;28(2):208-15.
23. Postigo JML, Hernández-Viadel JV, Trives JJR. Efficacy of a Group Memory Training Method for Older Adults Based on Visualization and Association Techniques: A Randomized, Controlled Trial with a Placebo Group. Applied Cognitive Psychology. 2010;24:956-68.
24. Liou H, Stonnington CM, Shah AA, Buckner-Petty SA, Locke DEC. Compensatory and Lifestyle-Based Brain Health Program for Subjective Cognitive Decline: Self-Implementation versus Coaching. Brain Sci. 2021;11(10).
25. Marchant NL, Barnhofer T, Coueron R, Wirth M, Lutz A, Arenaza-Urquijo EM, et al. Effects of a Mindfulness-Based Intervention versus Health Self-Management on Subclinical Anxiety in Older Adults with Subjective Cognitive Decline: The SCD-Well Randomized Superiority Trial. Psychother Psychosom. 2021;90(5):341-50.
26. McEwen SC, Siddarth P, Rahi B, Kim Y, Mui W, Wu P, et al. Simultaneous Aerobic Exercise and Memory Training Program in Older Adults with Subjective Memory Impairments. J Alzheimers Dis. 2018;62(2):795-806.
27. Oh SJ, Seo S, Lee JH, Song MJ, Shin MS. Effects of smartphone-based memory training for older adults with subjective memory complaints: a randomized controlled trial. Aging Ment Health. 2018;22(4):526-34.
28. Pereira-Morales AJ, Cruz-Salinas AF, Aponte J, Pereira-Manrique F. Efficacy of a computer-based cognitive training program in older people with subjective memory complaints: a randomized study. Int J Neurosci. 2018;128(1):1-9.
29. Ramnath U, Rauch L, Lambert EV, Kolbe-Alexander T. Efficacy of interactive video gaming in older adults with memory complaints: A cluster-randomized exercise intervention. PLoS One. 2021;16(5):e0252016.
30. Smart CM, Segalowitz SJ, Mulligan BP, Koudys J, Gawryluk JR. Mindfulness Training for Older Adults with Subjective Cognitive Decline: Results from a Pilot Randomized Controlled Trial. J Alzheimers Dis. 2016;52(2):757-74.
31. Stroehlein JK, Vieluf S, Zimmer P, Schenk A, Oberste M, Goelz C, et al. Learning to play golf for elderly people with subjective memory complaints: feasibility of a single-blinded randomized pilot trial. BMC Neurol. 2021;21(1):200.
32. Su H, Wang H, Meng L. The effects of Baduanjin exercise on the subjective memory complaint of older adults: A randomized controlled trial. Medicine (Baltimore). 2021;100(30):e25442.
33. Sun Q. Effect and Mechanism of Baduanjin on Episodic Memory with SCD Patients-Based on the posterior Default Mode Network. PhD dissertation. Fujian University of Traditional Chinese Medicine; 2021. Accessed November 25, 2022. <https://chn.oversea.cnki.net/KCMS/detail/detail.aspx?dbcode=CDFD&dbname=CDFDLAST2022&filename=1021610265.nh&uniplatform=OVERSEA&v=s2R_qofYChSLXhrxI-omcQC5_TLqscWMctDV2_w945FQYKNl9SuGWUhcENjwK4_R>
34. Thana-Udom K, Siddarth P, Miller KJ, Dunkin JJ, Small GW, Ercoli LM. The Effect of Memory Training on Memory Control Beliefs in Older Adults with Subjective Memory Complaints. Exp Aging Res. 2021;47(2):131-44.
35. Valentijn SA, van Hooren SA, Bosma H, Touw DM, Jolles J, van Boxtel MP, et al. The effect of two types of memory training on subjective and objective memory performance in healthy individuals aged 55 years and older: a randomized controlled trial. Patient Educ Couns. 2005;57(1):106-14.
36. Xu S. Clinical Evaluation of the Effect of Tai Chi Training on Cognitive Function of the Elderly with Subjective Cognitive Decline. Master dissertation. Fujian University of Traditional Chinese Medicine; 2022. Accessed November 25, 2022.

<https://chn.oversea.cnki.net/KCMS/detail/detail.aspx?dbcode=CMFD&dbname=CMFDTEMP&filename=1022567488.nh&uniplatform=OVERSEA&v=BRU6EWBO6WAfl0eUMA389MXEvArfMVwry6mw_mvWbBskgDaHILUv2V2xL0D14mkZ>

1. Yin S, Zhu X, Li R, Huo L, Ren W, Niu Y, et al. Alleviated Anxiety Boosts Memory Training Gain in Older Adults with Subjective Memory Complaints: A Randomized Controlled Trial. Am J Geriatr Psychiatry. 2022;30(2):184-94.
2. Youn JH, Ryu SH, Lee JY, Park S, Cho SJ, Kwon H, et al. Brain structural changes after multi-strategic metamemory training in older adults with subjective memory complaints: A randomized controlled trial. Brain Behav. 2019;9(5):e01278.
3. Youn JH, Lee JY, Kim S, Ryu SH. Multistrategic memory training with the metamemory concept in healthy older adults. Psychiatry Investig. 2011;8(4):354-61.

# eAppendix 4. Study Characteristics

**4.1 Table 1. Study Characteristics of Included RCTs**

| **Study** | **Year** | **Country** | **Recruitment** | **Treatment** | **Participations (n)** | **Age (mean ± SD)** | **Female (%)** | **Research setting** | **Treatment Duration** |
| --- | --- | --- | --- | --- | --- | --- | --- | --- | --- |
| Bozoki A ^[1]^ | 2013 | USA | community | cognitive intervention | 32 | 67.20±6.37 | 50.0% | Not reported | 6 weeks |
|  |  |  |  | usual care | 28 | 70.80±6.81 | 67.9% |  |  |
| Brooks H ^[2]^ | 2021 | Canada | Not reported | cognitive intervention + occupational therapy | 14 | 68.30±5.90 | 64.3% | Hospital and home | 10 weeks |
|  |  |  |  | cognitive intervention | 12 | 69.00±5.00 | 25.0% |  |  |
| Cabral DF ^[3]^ | 2022 | Brazil | community | exercise + psychotherapy | 24 | 65.30±3.50 | 87.5% | Home | 4 weeks |
|  |  |  |  | wl | 22 | 66.90±6.10 | 90.9% |  |  |
| Cavalcante BR ^[4]^ | 2020 | Germany | community | exercise (Balance Group) | 22 | 71.00±6.00 | 77.0% | Gym | 12 weeks |
|  |  |  |  | exercise (Resistance Group) | 23 | 71.00±4.00 | 78.0% |  |  |
|  |  |  |  | usual care | 22 | 71.00±6.00 | 77.0% |  |  |
| Chan AS ^[5]^ | 2017 | China | community | cognitive intervention + psychotherapy | 42 | 68.34±4.42 | 35.7% | Not reported | 10 weeks |
|  |  |  |  | cognitive intervention | 26 | 69.50±6.89 | 80.7% |  |  |
| Cohen-Mansfield J ^[6]^ | 2015 | Israel | community | cognitive intervention | 15 | 72.80±3.78 | 60% | Institute of Medicine | 10 weeks |
|  |  |  |  | psychotherapy | 15 | 74.44±5.78 | 86.7% |  |  |
|  |  |  |  | occupational therapy | 14 | 73.21±5.97 | 71.4% |  |  |
| Cox KL ^[7]^ | 2021 | Australia | memory clinic and community | psychotherapy + exercise | 26 | 71.90±5.90 | 53.8% | Home | 24 weeks |
|  |  |  |  | usual care | 26 | 68.50±6.50 | 73.1% |  |  |
| Cuesta M ^[8]^ | 2022 | Canada | community | cognitive intervention | 19 | 69.70±5.50 | 57.9% | Not reported | 12 weeks |
|  |  |  |  | wl | 19 | 72.40±6.50 | 84.2% |  |  |
| Dawson D ^[9]^ | 2014 | Canada | community | occupational therapy | 10 | 74.10±8.77 | 90.0% | Institute of Medicine | 8 weeks |
|  |  |  |  | cognitive intervention + psychotherapy | 9 | 73.67±5.43 | 77.8% |  |  |
| Denny KG ^[10]^ | 2021 | USA | memory clinic and community | cognitive intervention + psychotherapy + exercise | 28 | 76.60±5.80 | 66.7% | Home | 10 weeks |
|  |  |  |  | wl | 29 | 73.20±6.80 | 93.1% |  |  |
| Fabre C ^[11]^ | 1999 | France | community | exercise (Aerobic Group) | 8 | 65.40±6.20 | NA | Not reported | 8 weeks |
|  |  |  |  | cognitive intervention | 8 | 67.50±3.40 | NA |  |  |
|  |  |  |  | cognitive intervention + exercise | 8 | 64.90±3.90 | NA |  |  |
|  |  |  |  | wl | 8 | 65.70±4.20 | NA |  |  |
| Fairchild JK ^[12]^ | 2010 | USA | community | cognitive intervention | 28 | 73.45±9.89 | 82.1% | Not reported | 6 weeks |
|  |  |  |  | usual care | 25 | 71.24±8.25 | 80.0% |  |  |
| Frankenmolen NL ^[13]^ | 2018 | Netherlands | community | cognitive intervention | 31 | 66.20±7.30 | 65.5% | Not reported | 7 weeks |
|  |  |  |  | active placebo | 29 | 68.00±7.80 | 65.5% |  |  |
| Fujita K ^[14]^ | 2021 | Japan | community | exercise (Aerobic Group) | 95 | 72.20 ±4.60 | 50.5% | Not reported | 26 weeks |
|  |  |  |  | exercise (Resistance Group) | 93 | 72.30±4.80 | 49.5% |  |  |
|  |  |  |  | exercise (Aerobic Group) + exercise (Resistance Group) | 98 | 72.60±4.50 | 41.8% |  |  |
|  |  |  |  | usual care | 102 | 72.10±4.60 | 51.0% |  |  |
| Hermes S ^[15]^ | 2019 | Germany | memory clinic | cognitive intervention | 8 | NA | 75.0% | Not reported | 10days |
|  |  |  |  | wl | 6 | NA | 50.0% |  |  |
| Hong YJ ^[16]^ | 2020 | South Korea | memory clinic and hospital | cognitive intervention + psychotherapy + exercise | 23 | 66.22±5.73 | 73.9% | Memory clinic | 12 weeks |
|  |  |  |  | Psychotherapy + exercise | 15 | 65.40±4.82 | 93.3% |  |  |
|  |  |  |  | wl | 18 | 65.83±4.89 | 66.7% |  |  |
| Hoogenhout EM ^[17]^ | 2012 | Netherlands | Not reported | cognitive intervention + psychotherapy | 30 | 66.00±4.23 | 100% | Institute of Medicine | 7 weeks |
|  |  |  |  | wl | 30 | 66.10±4.48 | 100% |  |  |
| Innes KE ^[18]^ | 2021 | USA | community | cognitive intervention + psychotherapy | 20 | 66.85±2.14 | 55.0% | Home | 12 weeks |
|  |  |  |  | usual care | 20 | 61.45±1.38 | 90.0% |  |  |
| Iuliano E ^[19]^ | 2017 | Italy | Not reported | exercise (Resistance Group) | 20 | 65.80±6.32 | 55.0% | Gym | 12 weeks |
|  |  |  |  | exercise (Aerobic Group) | 20 | 68.44±6.40 | 60.0% |  |  |
|  |  |  |  | exercise (Balance Group) | 20 | 66.67±5.83 | 65.0% |  |  |
|  |  |  |  | wl | 20 | 66.47±6.32 | 60.0% |  |  |
| Krause-Sorio B ^[20]^ | 2022 | USA | community | exercise (Balance Group) | 11 | NA | 100% | Not reported | 12 weeks |
|  |  |  |  | occupational therapy | 11 | NA | 100% |  |  |
| Kwok T ^[21]^ | 2013 | China | community | cognitive intervention | 86 | 77.41±6.75 | 87.2% | Not reported | 8 weeks |
|  |  |  |  | usual care | 90 | 73.50±7.35 | 83.3% |  |  |
| Kwok TC ^[22]^ | 2012 | China | community | cognitive intervention | 111 | 75.41±5.82 | 87.4% | Not reported | 12 weeks |
|  |  |  |  | usual care | 112 | 75.39±5.83 | 83.0% |  |  |
| Latorre-Postigo JM ^[23]^ | 2010 | Spain | community | cognitive intervention | 15 | 67.80±2.85 | 73.3% | Not reported | 5 weeks |
|  |  |  |  | usual care | 15 | 65.73±3.36 | 53.3% |  |  |
|  |  |  |  | wl | 15 | 67.40±2.99 | 66.7% |  |  |
| Liou H ^[24]^ | 2021 | USA | clinic | cognitive intervention + exercise | 7 | NA | 71.4% | Not reported | 10 weeks |
|  |  |  |  | usual care | 10 | NA | 60.0% |  |  |
| Marchant NL ^[25]^ | 2021 | UK | memory clinic | psychotherapy | 73 | 72.10±7.60 | 64.4% | Memory clinic | 8 weeks |
|  |  |  |  | usual care | 74 | 73.30±6.20 | 64.9% |  |  |
| McEwen SC ^[26]^ | 2018 | USA | Not reported | cognitive intervention + exercise | 29 | 67.00±5.10 | 65.5% | Institute of Medicine or Patient Room | 4 weeks |
|  |  |  |  | cognitive intervention | 26 | 65.40±3.00 | 73.1% |  |  |
| Oh SJ ^[27]^ | 2018 | South Korea | Not reported | cognitive intervention | 18 | 59.28±5.11 | 50.0% | Home | 8 weeks |
|  |  |  |  | active placebo | 19 | 58.79±5.00 | 52.6% |  |  |
|  |  |  |  | wl | 16 | 59.94±5.17 | 56.3% |  |  |
| Pereira-Morales AJ ^[28]^ | 2018 | Colombia | community | cognitive intervention + psychotherapy | 17 | 64.50±4.80 | 88.2% | Home | 8 weeks |
|  |  |  |  | cognitive intervention | 12 | 69.30±4.80 | 91.7% |  |  |
|  |  |  |  | usual care | 11 | 65.60±7.20 | 90.9% |  |  |
| Ramnath U ^[29]^ | 2021 | South Africa | community | cognitive intervention + exercise | 23 | 70.80±4.52 | NA | Nursing home or assisted living | 12 weeks |
|  |  |  |  | usual care | 22 | 74.14±5.80 | NA |  |  |
| Smart CM ^[30]^ | 2016 | Canada | Not reported | psychotherapy | 15 | 69.60±3.58 | 73.3% | Not reported | 8 weeks |
|  |  |  |  | usual care | 23 | 70.00±3.45 | 39.1% |  |  |
| Stroehlein JK ^[31]^ | 2021 | Germany | Not reported | exercise (Balance Group) | 23 | 67.87±4.70 | 56.5% | Institute of Medicine | 22 weeks |
|  |  |  |  | wl | 19 | 67.89±3.90 | 52.6% |  |  |
| Su H ^[32]^ | 2021 | China | community | exercise (Balance Group) | 32 | 64.40±6.57 | 54.3% | Not reported | 12 weeks |
|  |  |  |  | active placebo | 33 | 65.37±6.31 | 48.6% |  |  |
| Sun Q ^[33]^ | 2021 | China | community | exercise (Balance Group) | 34 | 65.94±4.35 | 47.1% | Not reported | 24 weeks |
|  |  |  |  | exercise (Aerobic Group) | 32 | 66.28±5.56 | 40.6% |  |  |
|  |  |  |  | usual care | 28 | 67.11±5.09 | 57.1% |  |  |
| Thana-Udom K ^[34]^ | 2021 | USA | community | cognitive intervention | 23 | 67.26 ±6.87 | 26.1% | Not reported | 7 weeks |
|  |  |  |  | psychotherapy | 23 | 67.78 ±6.57 | 17.3% |  |  |
|  |  |  |  | wl | 17 | 70.29± 6.74 | 47.1% |  |  |
| Valentijn SA ^[35]^ | 2005 | Netherlands | community | cognitive intervention + psychotherapy | 53 | 69.32±7.77 | 70.0% | Not reported | 8 weeks |
|  |  |  |  | cognitive intervention | 43 | 68.07±6.58 | 63.0% |  |  |
|  |  |  |  | wl | 43 | 68.30±8.03 | 63.0% |  |  |
| Xu S ^[36]^ | 2022 | China | community | usual care | 26 | NA | 69.2% | Not reported | 24 weeks |
|  |  |  |  | exercise (Balance Group) | 26 | NA | 61.5% |  |  |
|  |  |  |  | exercise (Aerobic Group) | 26 | NA | 65.4% |  |  |
| Yin S ^[37]^ | 2021 | China | community | cognitive intervention | 38 | 71.34±6.63 | 68.4% | Not reported | 7 weeks |
|  |  |  |  | cognitive intervention +psychotherapy | 42 | 72.90±5.29 | 59.5% |  |  |
|  |  |  |  | usual care | 44 | 71.93±6.46 | 59.1% |  |  |
| Youn JH ^[38]^ | 2019 | South Korea | memory clinic and community | cognitive intervention + psychotherapy | 112 | 69.93±5.10 | 57.1% | Not reported | 10 weeks |
|  |  |  |  | wl | 89 | 69.11±4.60 | 67.4% |  |  |
| Youn JH ^[39]^ | 2011 | South Korea | memory clinic and community | cognitive intervention | 16 | 68.75±4.60 | 62.5% | Not reported | 10 weeks |
|  |  |  |  | wl | 16 | 69.00±3.45 | 56.3% |  |  |

Note: Reference numbers here correspond to eAppendix 3 (List of the included studies).

**4.2 Table 2. The distribution of effect modifiers across studies**

| **Characteristic** | **Studies, n(%)** | **Characteristic** | **Studies, n(%)** |
| --- | --- | --- | --- |
| **Recruitment sourse** |  | **Women enrolled in study** |  |
| *Memory clinic and community* | 4 (10.3) | *0%-49%* | 4 (10.3) |
| *Memory clinic and hospital* | 1 (2.6) | *50%-100%* | 33 (84.6) |
| *Memory clinic* | 2 (5.1) | *Not reported* | 2 (5.1) |
| *Clinic* | 1 (2.6) | **Intervention duration (weeks)** |  |
| *Community* | 24 (61.5) | *＜10w* | 18 (46.2) |
| *Not reported* | 7 (17.9) | *10w-19w* | 16 (41.0) |
| **Number of participants** |  | *≥20w* | 5 (12.8) |
| *≤50* | 16 (41.0) |  |  |
| *51-99* | 16 (41.0) |  |  |
| *100-149* | 3 (7.7) |  |  |
| *≥150* | 4 (10.3) |  |  |
| **Mean age of participants (years)** |  |  |  |
| *＜60* | 1 (2.6) |  |  |
| *60-64.99* | 2 (5.1) |  |  |
| *65-69.99* | 19 (48.7) |  |  |
| *≥70* | 13 (33.3) |  |  |
| *Not reported* | 4 (10.3) |  |  |

# eAppendix 5. Bias Assessment of Eligible Studies

| **Study** | **Random sequence**  **Generation** | **Allocation concealment** | **Blinding of participants and personnel** | **Blinding of outcome assessment** | **Incomplete outcome data** | **Selective reporting** | **Other bias** | **Overall risk of bias** |
| --- | --- | --- | --- | --- | --- | --- | --- | --- |
| Bozoki A | Unclear | Unclear | High | Unclear | Unclear | Low | Low | High |
| Brooks H | Low | Low | Low | Low | Low | Unclear | Low | Low |
| Cabral DF | Low | Low | High | High | Low | Low | Low | High |
| Cavalcante BR | Low | Low | Unclear | High | Low | Low | Low | High |
| Chan AS | Unclear | Unclear | Unclear | Low | Low | Unclear | Low | Unclear |
| Cohen-Mansfield J | Unclear | Unclear | Unclear | Low | Low | Unclear | Low | Unclear |
| Cox KL | Low | Low | High | Low | Low | Low | Low | High |
| Cuesta M | Unclear | Unclear | Unclear | Unclear | Low | Unclear | Low | Unclear |
| Dawson D | Low | Low | Low | Low | Low | Low | Low | Low |
| Denny KG | Low | Low | Unclear | Unclear | Low | Low | Low | Low |
| Fabre C | Unclear | Unclear | Unclear | Unclear | Unclear | Unclear | Low | Unclear |
| Fairchild JK | Unclear | Unclear | Unclear | Unclear | Low | Unclear | Low | Unclear |
| Frankenmolen NL | Low | Low | Low | Low | Low | Unclear | Low | Low |
| Fujita K | Low | Low | Unclear | Unclear | Low | Low | Low | Low |
| Hermes S | Unclear | Unclear | Unclear | Unclear | Low | Low | Low | Unclear |
| Hong YJ | Low | Low | Unclear | Low | Low | Low | Low | Low |
| Hoogenhout EM | Low | Low | Unclear | Low | Low | Low | Low | Low |
| Innes KE | Low | Low | Low | Low | Low | Unclear | Low | Low |
| Iuliano E | Low | Low | Unclear | Low | Low | Unclear | Low | Low |
| Krause-Sorio B | Low | Low | Unclear | Unclear | Low | Unclear | Low | Low |
| Kwok T | Unclear | Unclear | Unclear | Low | Unclear | Unclear | Low | Unclear |
| Kwok TC | Unclear | Unclear | High | Low | Low | Unclear | Low | High |
| Latorre-Postigo JM | Low | Low | Low | Low | Low | Unclear | Low | Low |
| Liou H | Low | Unclear | Unclear | Unclear | Unclear | Unclear | Low | Unclear |
| Marchant NL | Low | Low | Low | Low | Low | Low | Low | Low |
| McEwen SC | Unclear | Unclear | High | Unclear | Low | Unclear | Low | High |
| Oh SJ | Unclear | Unclear | Unclear | Unclear | Low | Unclear | Low | Unclear |
| Pereira-Morales AJ | Unclear | Unclear | Unclear | Unclear | Unclear | Unclear | Low | Unclear |
| Qianqian Sun | Low | Low | Low | Low | Low | Low | Low | Low |
| Ramnath U | Unclear | Unclear | High | Unclear | Low | Low | Low | High |
| Shurui Xu | Low | Low | Low | Low | Low | Low | Low | Low |
| Smart CM | Unclear | Unclear | Low | Low | Low | Unclear | Low | Low |
| Stroehlein JK | Low | Low | Unclear | Unclear | Low | Low | Low | Low |
| Su H | Low | Low | Unclear | Unclear | Low | Unclear | Low | Low |
| Thana-Udom K | Unclear | Unclear | Unclear | Unclear | Low | Low | Low | Unclear |
| Valentijn SA | Low | Low | Low | Low | Low | Unclear | Low | Low |
| Yin S | Low | Low | Low | Low | Low | Low | Low | Low |
| Youn JH 2019 | Low | Low | Unclear | Unclear | Low | Unclear | Low | Low |
| Youn JH 2011 | Unclear | Unclear | Unclear | Unclear | Low | Unclear | Low | Unclear |

# eAppendix 6. Pairwise Meta-analysis of forest plots

**6.1 Pairwise meta-analyses for subjective memory complaints**


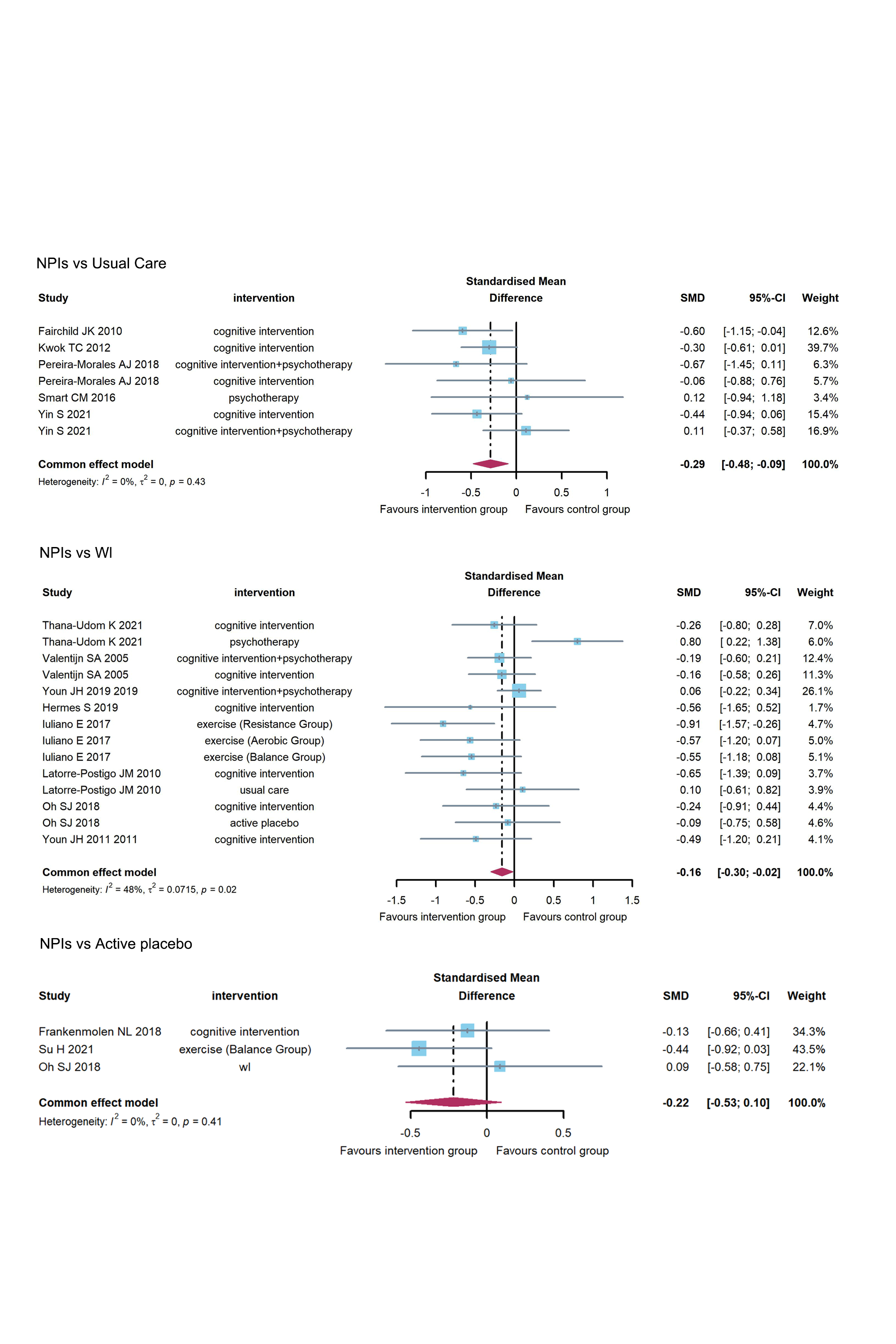


**6.2 Pairwise meta-analyses for global cognitive function**


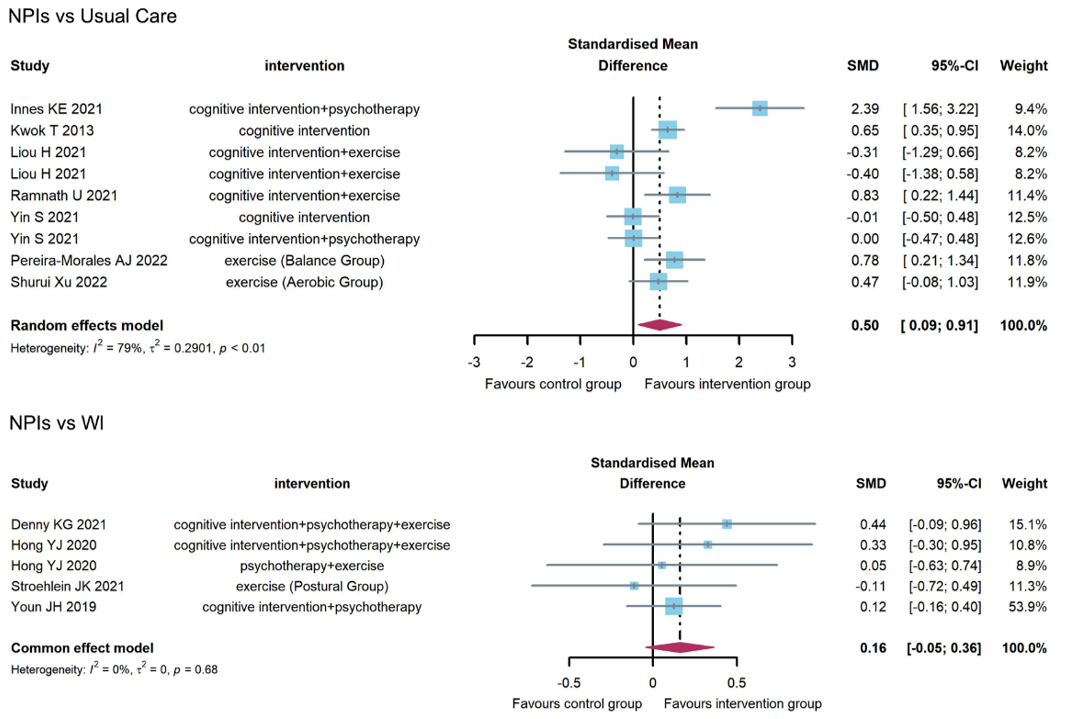


**6.3 Pairwise meta-analyses for language function**


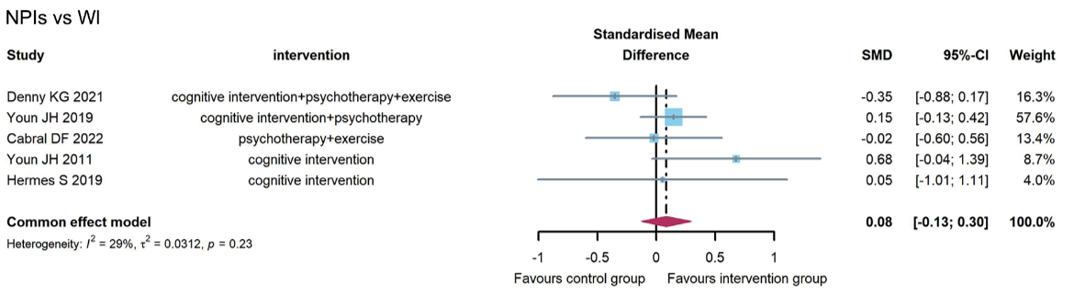


**6.4 Pairwise meta-analyses for executive function**


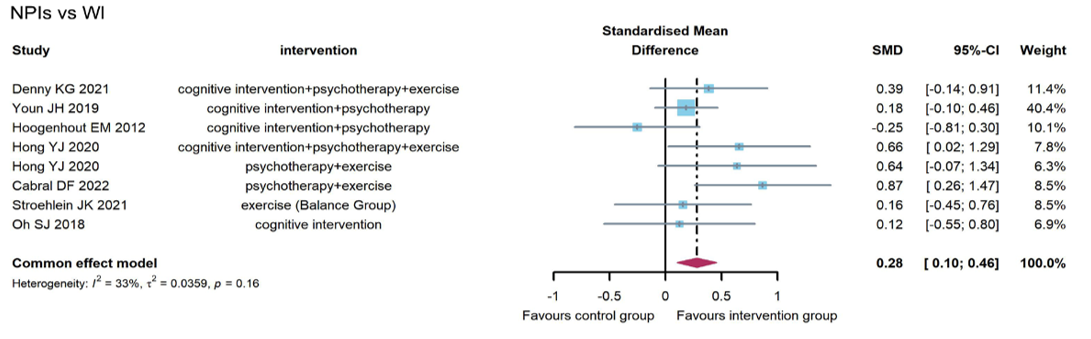


**6.5 Pairwise meta-analyses for visuospatial ability**


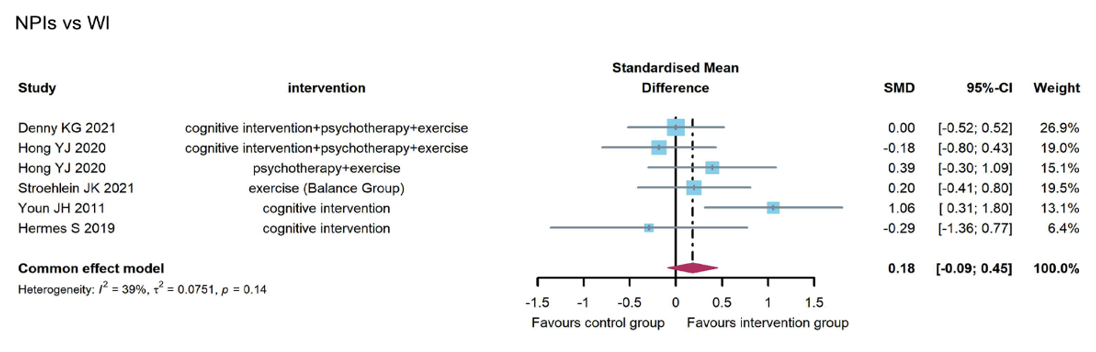


**6.6 Pairwise meta-analyses for attention**


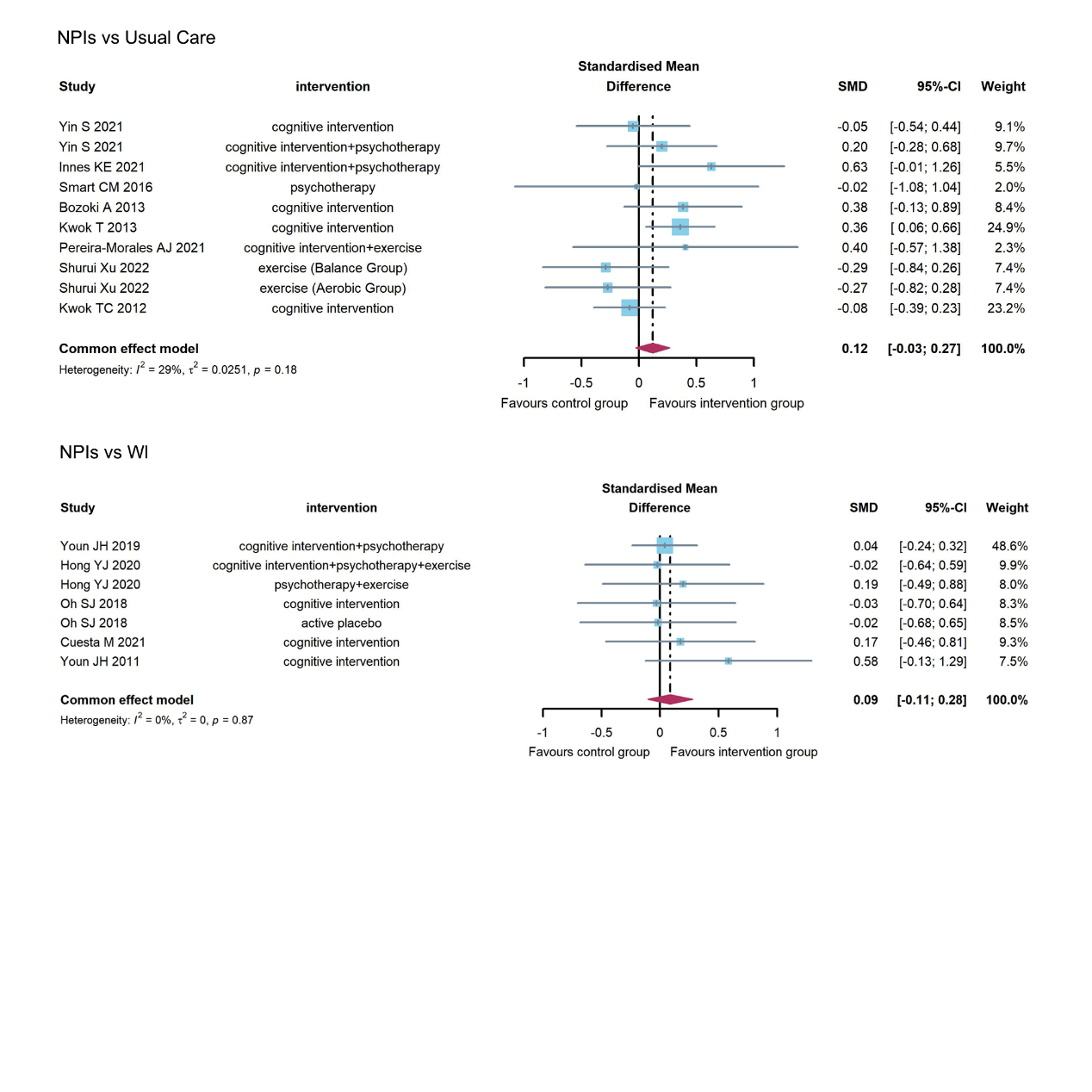


**6.7 Pairwise meta-analyses for activities of daily living**


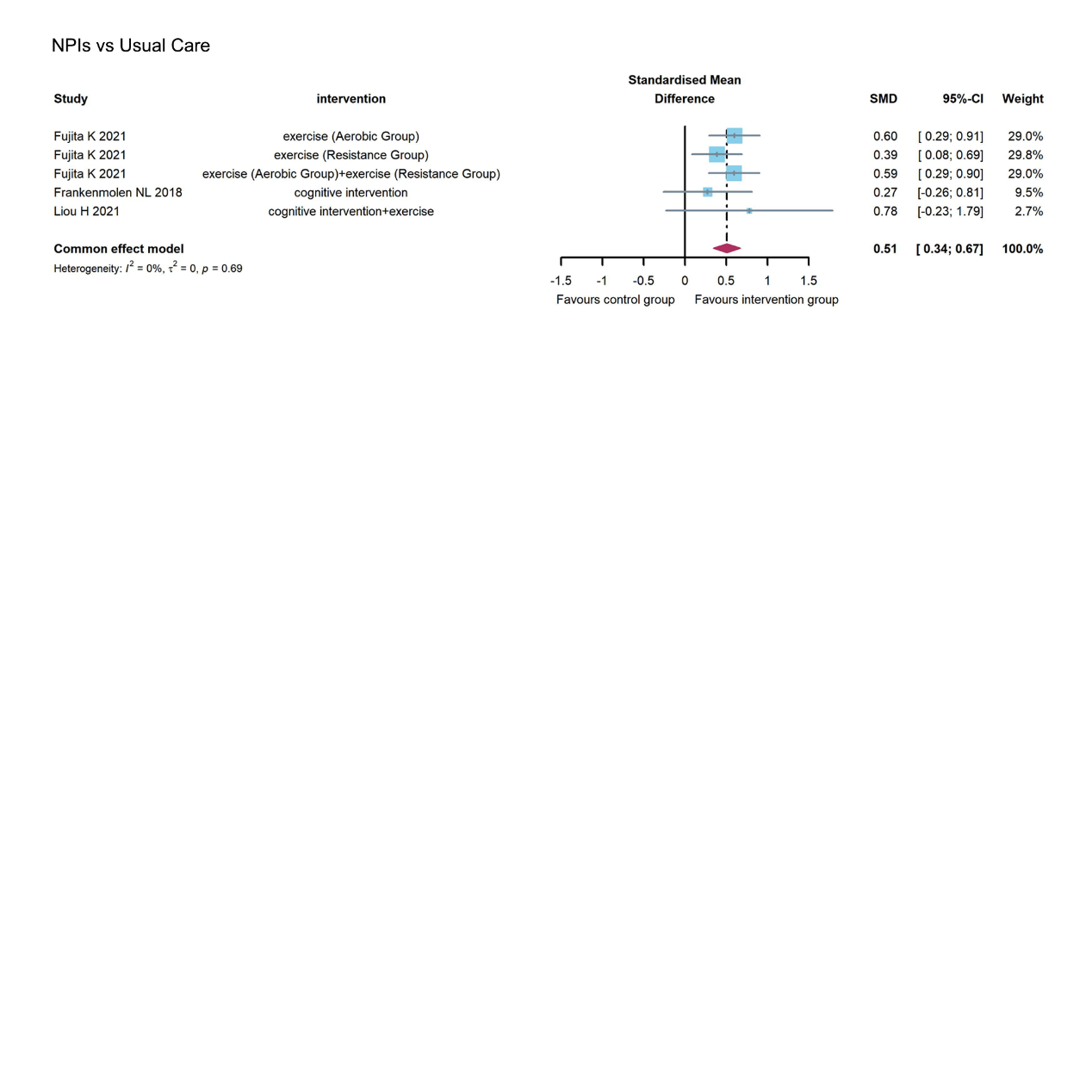


**6.8 Pairwise meta-analyses for anxiety**


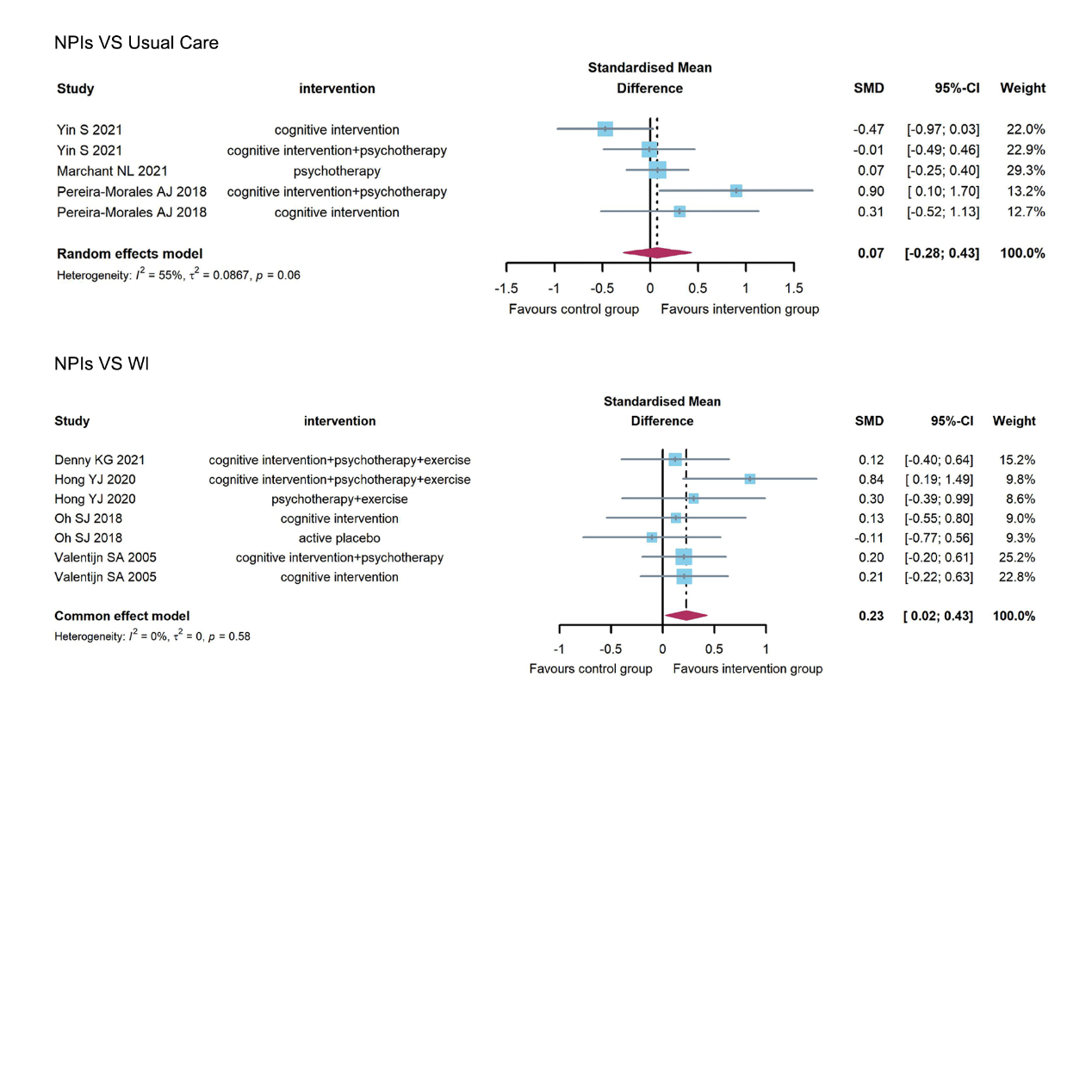


**6.9 Pairwise meta-analyses for depression**


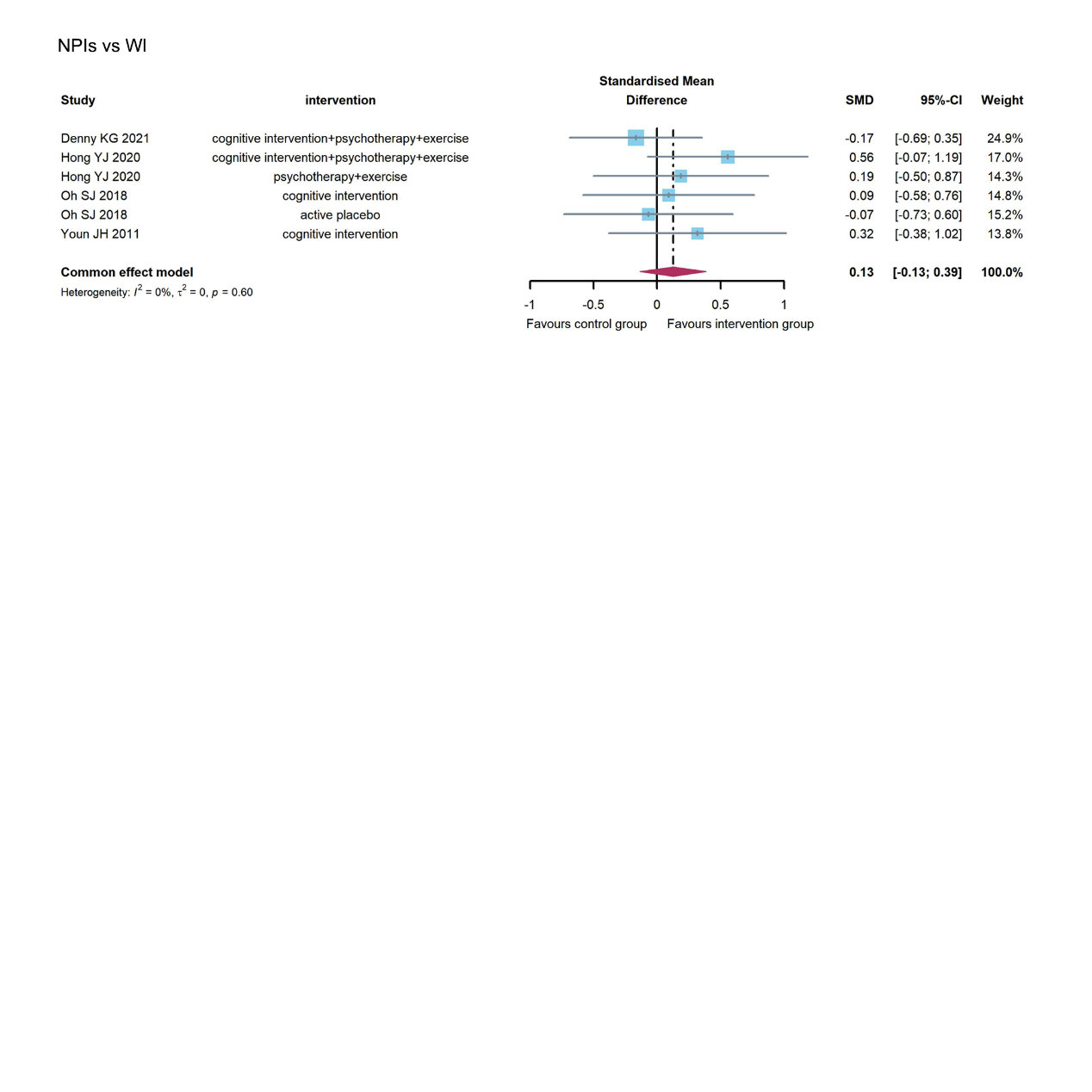


**6.10 Pairwise meta-analyses for physical health**

**
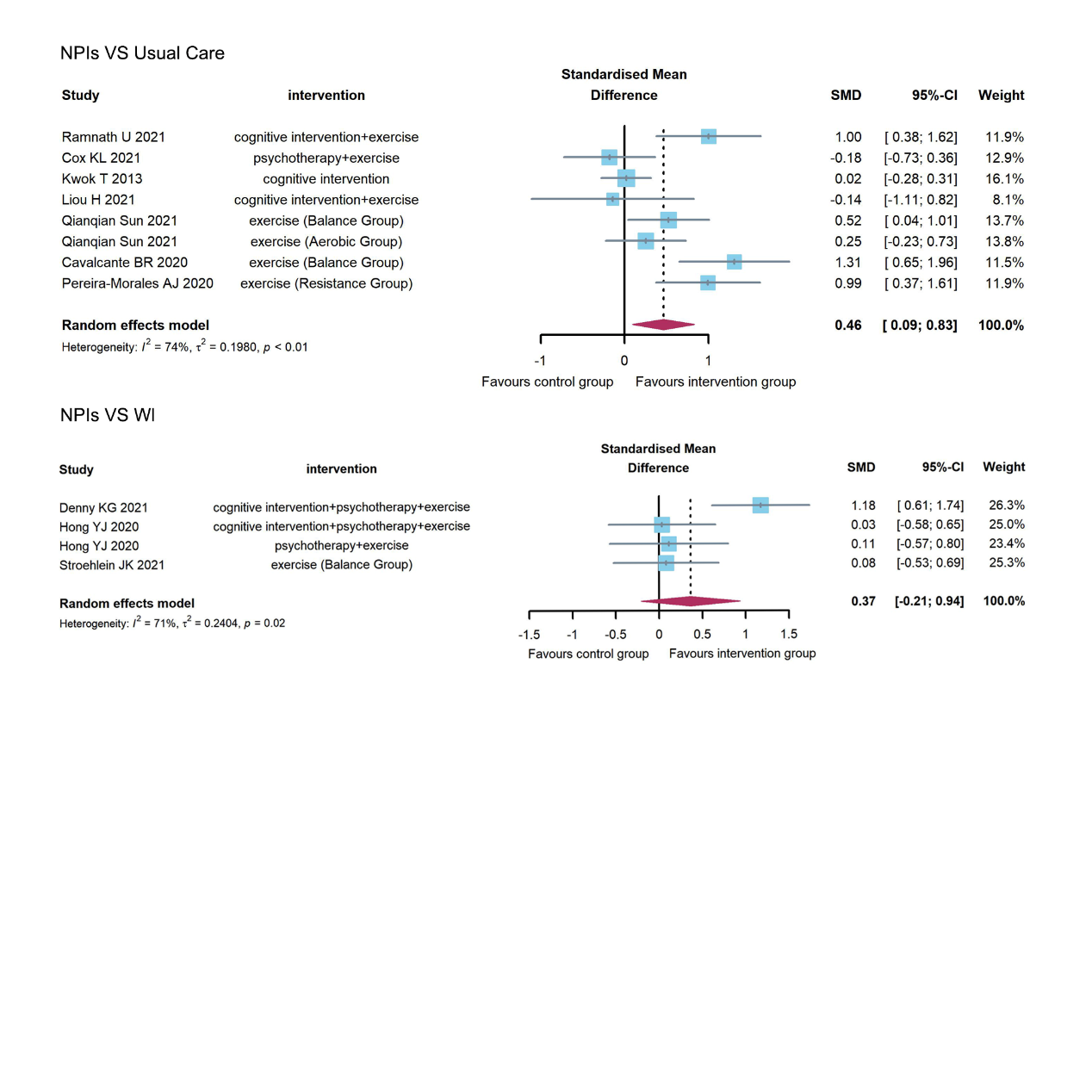
**

# eAppendix 7. Network Meta-analysis of forest plots and p-scores

**7.1 Subjective memory complaints; wl (SMD=0; p-score=0.310)**


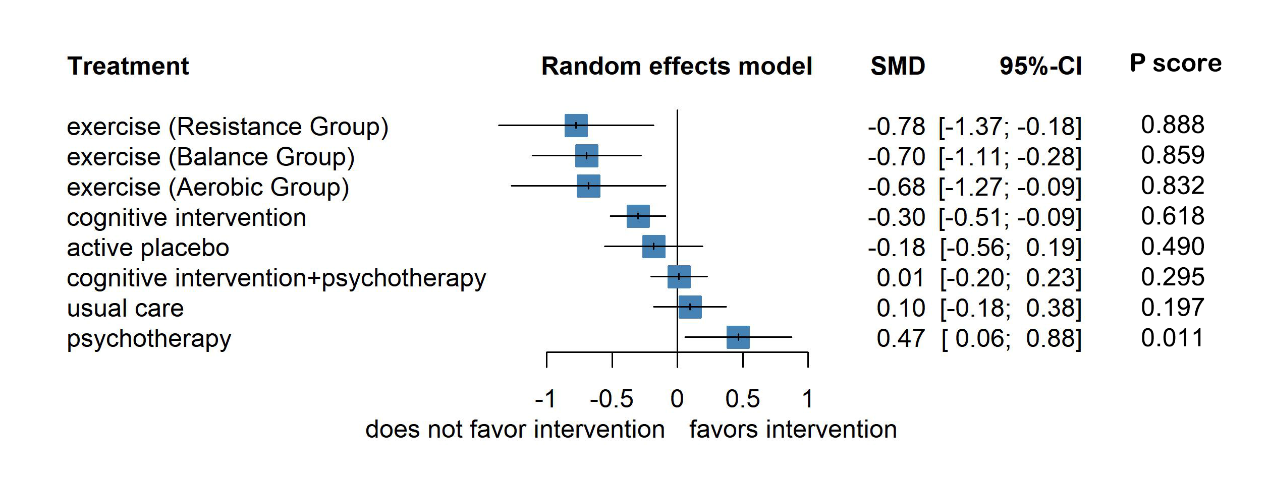


**7.2 Global cognitive function; wl (SMD=0; p-score=0.563)**


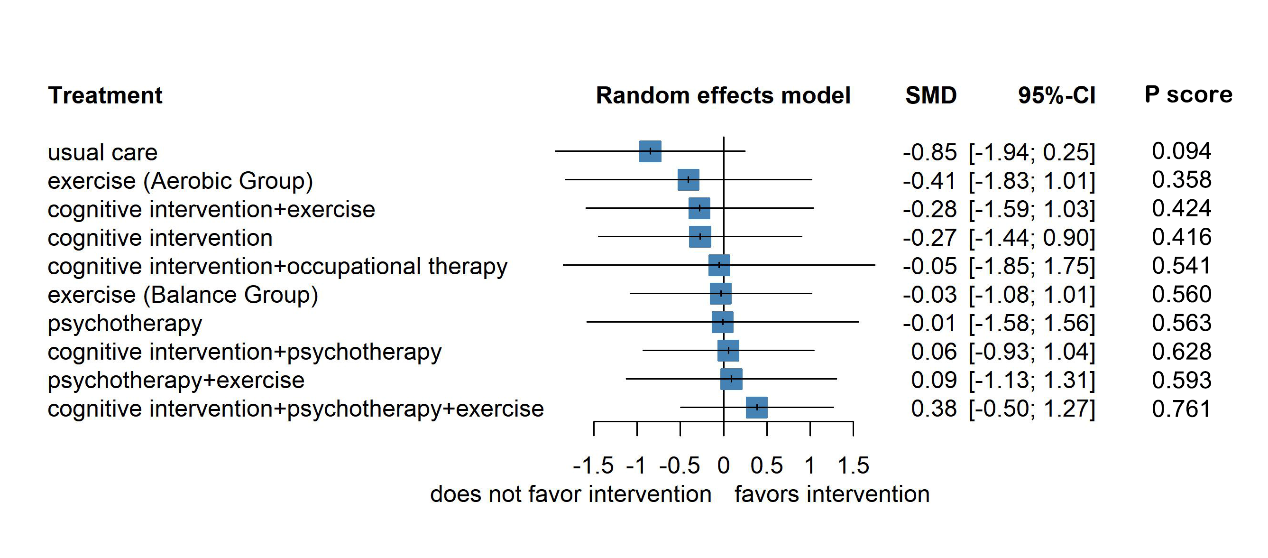


**7.3 Language function; wl (SMD=0; p-score=0.200)**


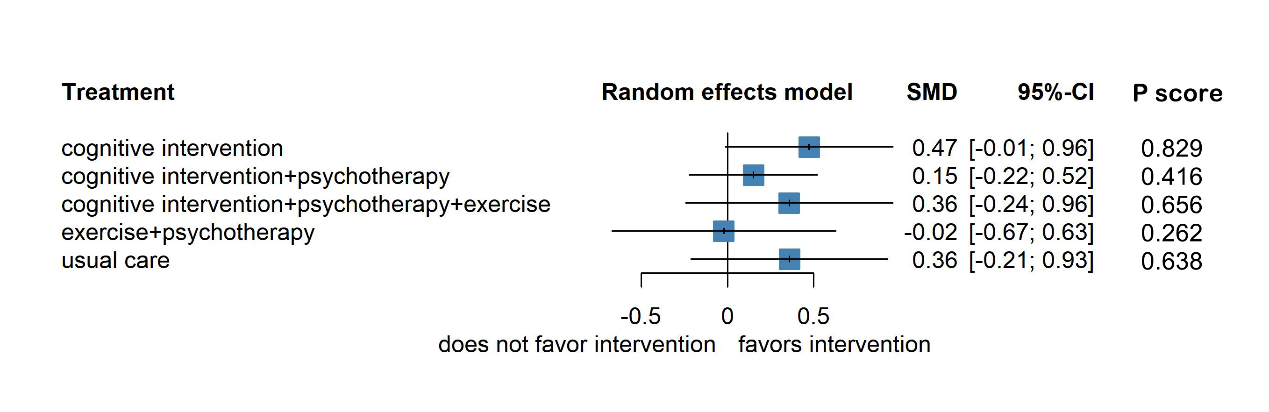


**7.4 Executive function; wl (SMD=0; p-score=0.380)**


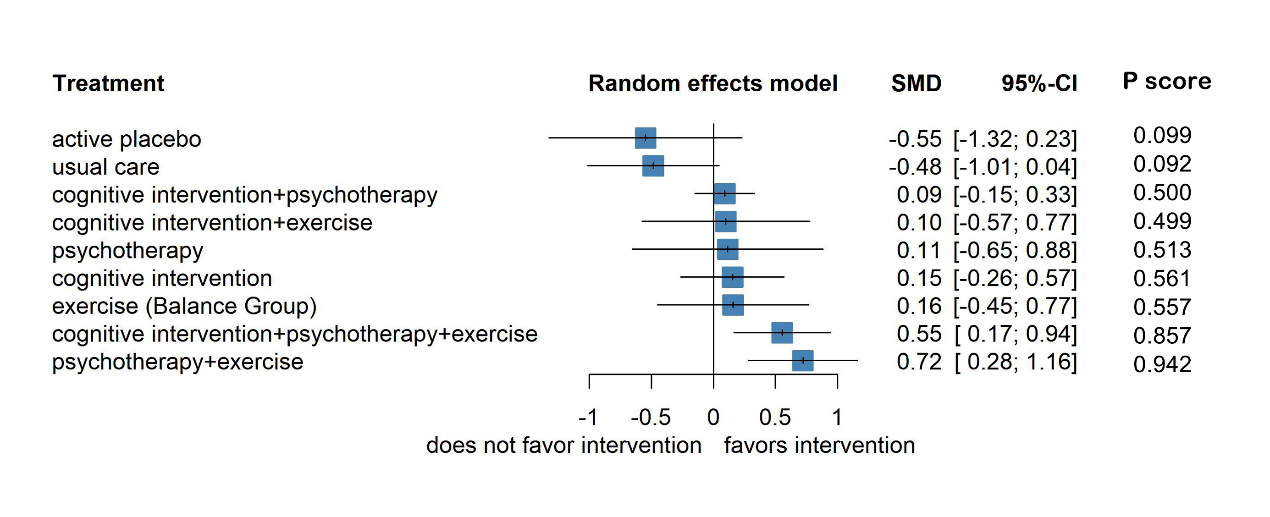


**7.5 Visuospatial ability; wl (SMD=0; p-score=0.229)**


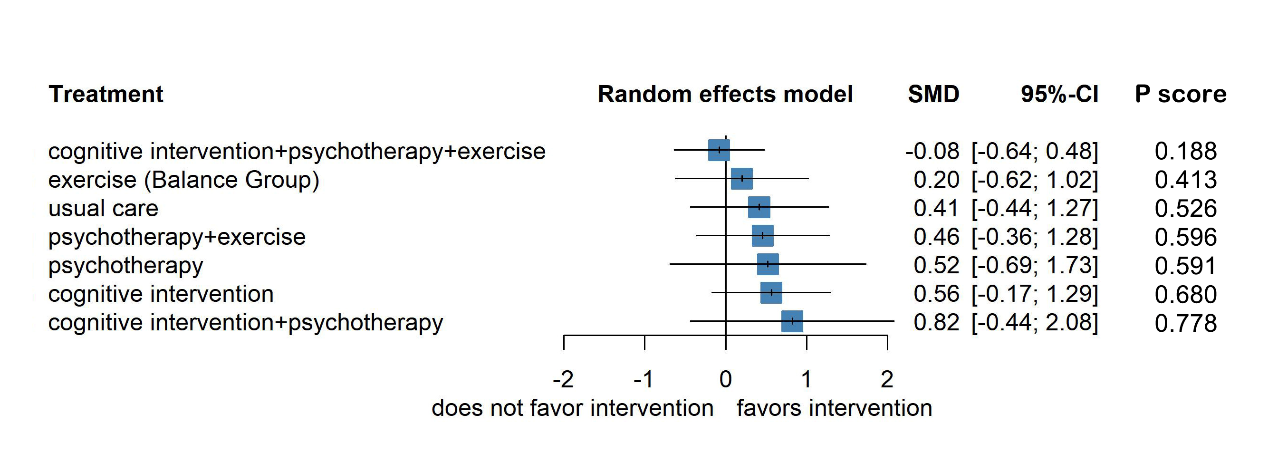


**7.6 Attention; wl (SMD=0; p-score=0.425)**


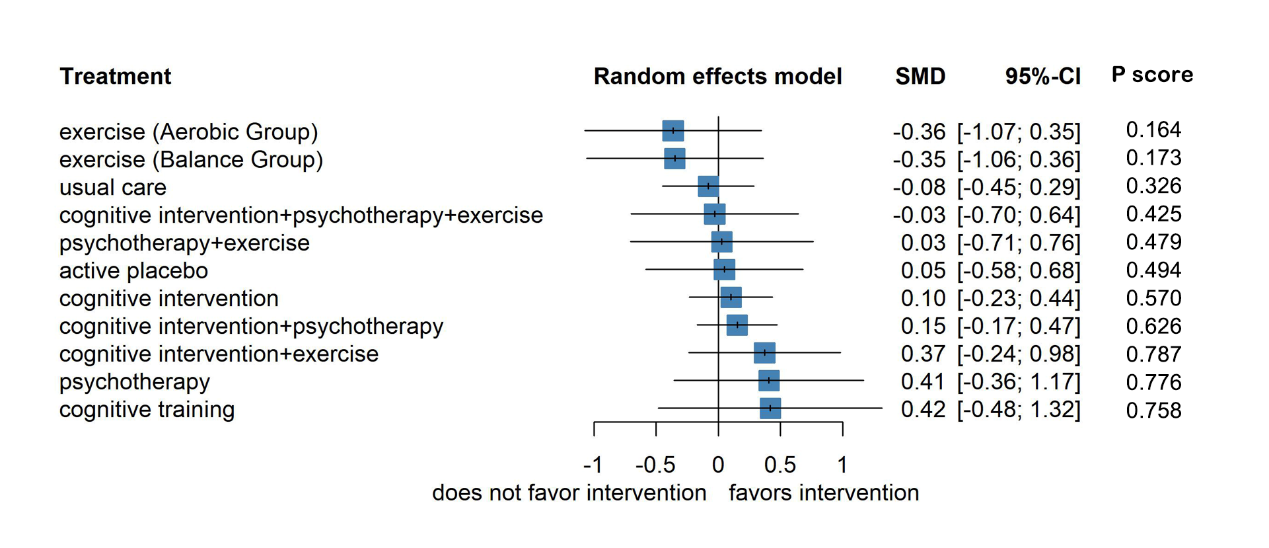


**7.7 Anxiety; wl (SMD=0; p-score=0.698)**


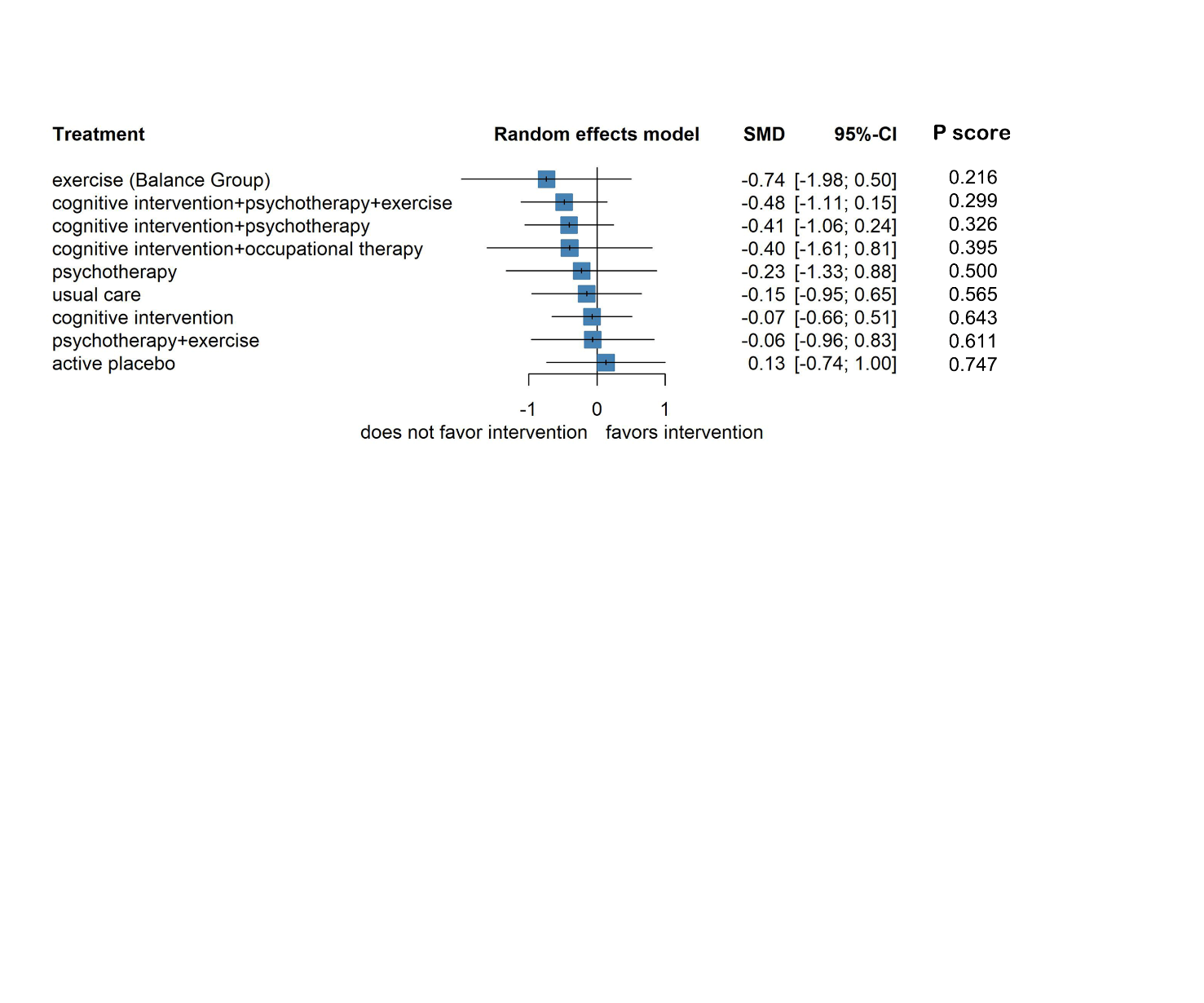


**7.8 Depression; wl (SMD=0; p-score=0.747)**


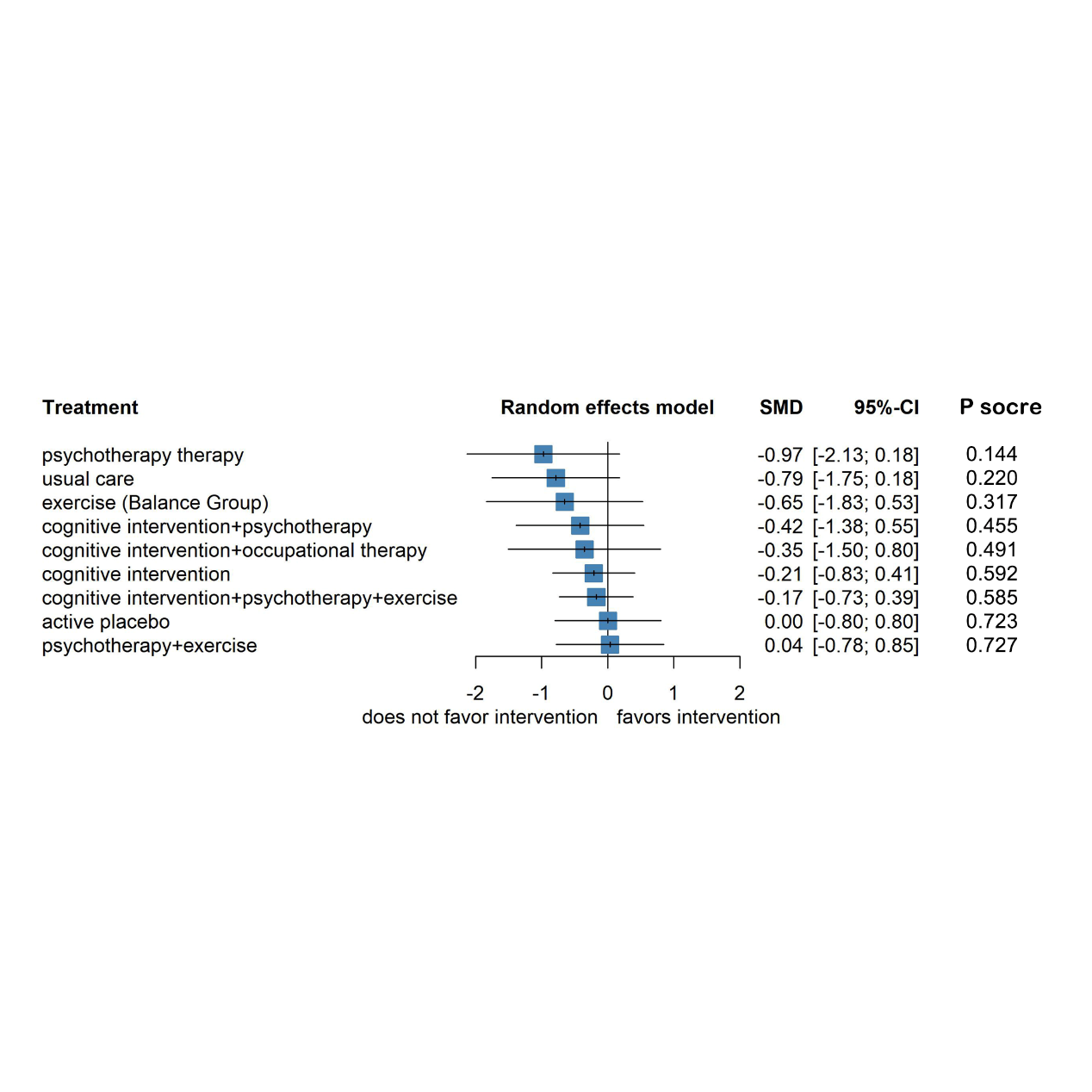


# eAppendix 8. Comparison of network meta-analysis, iSMD (95%CI)

**8.1 Subjective memory complaints**

| Active placebo | 0.15 (-0.27 to 0.57) | NA | NA | 0.45 (-0.04 to 0.94) | NA | NA | NA | -0.09 (-0.77 to 0.59) |
| --- | --- | --- | --- | --- | --- | --- | --- | --- |
| 0.12 (-0.23 to 0.47) | Cognitive intervention | **-0.27 (-0.53 to -0.01)** | NA | 0.74 (-0.13 to 1.61) | NA | **-0.85 (-1.32 to -0.39)** | **-0.39 (-0.62 to -0.16)** | **-0.32 (-0.58 to -0.06)** |
| -0.19 (-0.59 to 0.20) | **-0.31 (-0.53 to -0.10)** | Cognitive intervention  +Psychotherapy | NA | NA | NA | 0.22 (-0.65 to 1.10) | -0.11 (-0.53 to 0.31) | -0.03 (-0.28 to 0.22) |
| 0.50 (-0.14 to 1.14) | 0.38 (-0.23 to 0.99) | **0.69 (0.07 to 1.31)** | Exercise (Aerobic Group) | -0.02 (-0.66 to 0.61) | 0.09 (-0.54 to 0.73) | NA | NA | -0.64 (-1.28 to 0.00) |
| **0.51 (0.12 to 0.91)** | 0.39 (-0.02 to 0.81) | **0.71 (0.27 to 1.15)** | 0.01 (-0.57 to 0.60) | Exercise (Balance Group) | 0.12 (-0.52 to 0.75) | NA | NA | -0.62 (-1.26 to 0.02) |
| 0.59 (-0.05 to 1.24) | 0.47 (-0.14 to 1.09) | **0.79 (0.17 to 1.41)** | 0.10 (-0.54 to 0.73) | 0.08 (-0.51 to 0.67) | Exercise (Resistance Group) | NA | NA | **-0.74 (-1.38 to -0.09)** |
| **-0.65 (-1.17 to -0.13)** | **-0.77 (-1.16 to -0.37)** | **-0.45 (-0.87 to -0.04)** | **-1.15 (-1.86 to -0.44)** | **-1.16 (-1.72 to -0.60)** | **-1.24 (-1.95 to -0.53)** | Psychotherapy | 0.13 (-0.94 to 1.19) | **0.76 (0.18 to 1.35)** |
| -0.28 (-0.69 to 0.13) | **-0.40 (-0.62 to -0.18)** | -0.08 (-0.35 to 0.19) | **-0.78 (-1.42 to -0.14)** | **-0.79 (-1.25 to -0.33)** | **-0.87 (-1.51 to -0.23)** | 0.37 (-0.06 to 0.80) | Usual care | 0.00 (-0.73 to 0.73) |
| -0.18 (-0.56 to 0.19) | **-0.30 (-0.51 to -0.09)** | 0.01 (-0.20 to 0.23) | **-0.68 (-1.27 to -0.09)** | **-0.70 (-1.11 to -0.28)** | **-0.78 (-1.37 to -0.18)** | **0.47 (0.06 to 0.88)** | 0.10 (-0.18 to 0.38) | Wl |

**8.2 General cognitive function**

| Cognitive intervention | -0.35 (-1.58 to 0.88) | -0.22 (-1.59 to 1.15) | 0.11 (-0.81 to 1.03) | NA | NA | NA | 0.02 (-1.34 to 1.39) | NA | 0.34 (-0.49 to 1.17) | NA |
| --- | --- | --- | --- | --- | --- | --- | --- | --- | --- | --- |
| 0.01 (-0.86 to 0.87) | Cognitive intervention  + Exercise | NA | NA | NA | NA | NA | NA | NA | 0.35 (-0.61 to 1.31) | NA |
| -0.22 (-1.59 to 1.15) | -0.23 (-1.85 to 1.39) | Cognitive intervention  + Occupational therapy | NA | NA | NA | NA | NA | NA | NA | NA |
| -0.33 (-1.11 to 0.45) | -0.34 (-1.35 to 0.68) | -0.11 (-1.68 to 1.47) | Cognitive intervention  + Psychotherapy | NA | NA | NA | -0.25 (-1.66 to 1.16) | NA | **1.06 (0.15 to 1.97)** | 0.12 (-1.02 to 1.27) |
| -0.66 (-2.12 to 0.81) | -0.66 (-2.25 to 0.92) | -0.43 (-2.44 to 1.57) | -0.33 (-1.65 to 0.99) | Cognitive intervention  + Psychotherapy  + Exercise | NA | NA | NA | 0.26 (-1.02 to 1.55) | NA | 0.38 (-0.50 to 1.27) |
| 0.14 (-1.21 to 1.48) | 0.13 (-1.29 to 1.55) | 0.36 (-1.56 to 2.28) | 0.46 (-0.87 to 1.79) | 0.79 (-0.88 to 2.46) | Exercise (Aerobic Group) | -0.41 (-1.65 to 0.82) | NA | NA | 0.48 (-0.76 to 1.72) | NA |
| -0.24 (-1.42 to 0.94) | -0.25 (-1.53 to 1.04) | -0.02 (-1.83 to 1.79) | 0.09 (-1.03 to 1.20) | 0.42 (-0.95 to 1.79) | -0.37 (-1.56 to 0.81) | Exercise (Balance Group) | NA | NA | 0.89 (-0.35 to 2.14) | -0.12 (-1.38 to 1.15) |
| -0.26 (-1.51 to 0.99) | -0.27 (-1.74 to 1.20) | -0.04 (-1.89 to 1.81) | 0.07 (-1.20 to 1.33) | 0.39 (-1.40 to 2.19) | -0.40 (-2.15 to 1.35) | -0.02 (-1.63 to 1.59) | Psychotherapy | NA | NA | NA |
| -0.36 (-2.05 to 1.33) | -0.37 (-2.16 to 1.42) | -0.14 (-2.32 to 2.03) | -0.03 (-1.60 to 1.53) | 0.29 (-0.91 to 1.50) | -0.50 (-2.37 to 1.37) | -0.12 (-1.73 to 1.48) | -0.10 (-2.08 to 1.88) | Psychotherapy  + Exercise | NA | 0.06 (-1.25 to 1.36) |
| 0.58 (-0.11 to 1.26) | 0.57 (-0.23 to 1.37) | 0.80 (-0.73 to 2.33) | **0.90 (0.16 to 1.65)** | 1.23 (-0.17 to 2.64) | 0.44 (-0.75 to 1.63) | 0.81 (-0.22 to 1.85) | 0.84 (-0.50 to 2.17) | 0.94 (-0.70 to 2.57) | Usual care | NA |
| -0.27 (-1.44 to 0.90) | -0.28 (-1.59 to 1.03) | -0.05 (-1.85 to 1.75) | 0.06 (-0.93 to 1.04) | 0.38 (-0.50 to 1.27) | -0.41 (-1.83 to 1.01) | -0.03 (-1.08 to 1.01) | -0.01 (-1.58 to 1.56) | 0.09 (-1.13 to 1.31) | -0.85 (-1.94 to 0.25) | Wl |

**8.3 Language function**

| Cognitive intervention | 0.31 (-0.33 to 0.96) | NA | NA | 0.11 (-0.19 to 0.41) | 0.48 (-0.15 to 1.12) |
| --- | --- | --- | --- | --- | --- |
| 0.32 (-0.17 to 0.81) | Cognitive intervention + Psychotherapy | NA | NA | NA | 0.15 (-0.26 to 0.55) |
| 0.12 (-0.66 to 0.89) | -0.21 (-0.92 to 0.50) | Cognitive intervention  + Psychotherapy + Exercise | NA | NA | 0.36 (-0.24 to 0.96) |
| 0.50 (-0.32 to 1.31) | 0.17 (-0.58 to 0.92) | 0.38 (-0.51 to 1.27) | Exercise + Psychotherapy | NA | -0.02 (-0.67 to 0.63) |
| 0.11 (-0.19 to 0.41) | -0.21 (-0.78 to 0.36) | -0.00 (-0.83 to 0.83) | -0.38 (-1.25 to 0.48) | Usual care | NA |
| 0.47 (-0.01 to 0.96) | 0.15 (-0.22 to 0.52) | 0.36 (-0.24 to 0.96) | -0.02 (-0.67 to 0.63) | 0.36 (-0.21 to 0.93) | Wl |

**8.4 Execution function**

| Active placebo | NA | NA | NA | NA | -0.71 (-1.19 to -0.22) | NA | NA | NA | NA |
| --- | --- | --- | --- | --- | --- | --- | --- | --- | --- |
| -0.70 (-1.58 to 0.18) | Cognitive intervention | 0.05 (-0.48 to 0.58) | 0.14 (-0.34 to 0.62) | NA | NA | 0.07 (-0.73 to 0.87) | NA | 0.41 (-0.42 to 1.23) | 0.13 (-0.55 to 0.80) |
| -0.65 (-1.68 to 0.38) | 0.05 (-0.48 to 0.58) | Cognitive intervention + Exercise | NA | NA | NA | NA | NA | NA | NA |
| -0.64 (-1.45 to 0.17) | 0.06 (-0.33 to 0.45) | 0.01 (-0.65 to 0.67) | Cognitive intervention + Psychotherapy | NA | NA | -0.06 (-0.92 to 0.81) | NA | **0.57 (0.08 to 1.07)** | 0.10 (-0.15 to 0.34) |
| -1.10 (-1.97 to -0.23) | -0.40 (-0.97 to 0.17) | -0.45 (-1.23 to 0.32) | -0.46 (-0.92 to -0.01) | Cognitive intervention + Psychotherapy  + Exercise | NA | NA | 0.04 (-0.61 to 0.69) | NA | **0.51 (0.11 to 0.92)** |
| -0.71 (-1.19 to -0.22) | -0.00 (-0.74 to 0.73) | -0.06 (-0.97 to 0.85) | -0.07 (-0.72 to 0.59) | 0.40 (-0.33 to 1.12) | Exercise (Balance Group) | NA | NA | NA | 0.16 (-0.45 to 0.77) |
| -0.66 (-1.75 to 0.43) | 0.04 (-0.69 to 0.77) | -0.01 (-0.92 to 0.89) | -0.02 (-0.77 to 0.72) | 0.44 (-0.42 to 1.30) | 0.04 (-0.94 to 1.02) | Psychotherapy | NA | NA | NA |
| -1.27 (-2.16 to -0.38) | -0.57 (-1.17 to 0.04) | -0.62 (-1.43 to 0.18) | -0.63 (-1.13 to -0.13) | -0.17 (-0.67 to 0.34) | -0.56 (-1.31 to 0.19) | -0.61 (-1.49 to 0.28) | Psychotherapy  + Exercise | NA | **0.78 (0.32 to 1.24)** |
| -0.06 (-1.00 to 0.88) | **0.64 (0.07 to 1.21)** | 0.58 (-0.19 to 1.36) | **0.58 (0.10 to 1.06)** | **1.04 (0.38 to 1.70)** | 0.64 (-0.16 to 1.45) | 0.60 (-0.27 to 1.47) | **1.21 (0.52 to 1.89)** | Usual care | NA |
| -0.55 (-1.32 to 0.23) | 0.15 (-0.26 to 0.57) | 0.10 (-0.57 to 0.77) | 0.09 (-0.15 to 0.33) | **0.55 (0.17 to 0.94)** | 0.16 (-0.45 to 0.77) | 0.11 (-0.65 to 0.88) | **0.72 (0.28 to 1.16)** | -0.48 (-1.01 to 0.04) | Wl |

**8.5 Visuospatial ability**

| Cognitive intervention | -0.26 (-1.28 to 0.77) | NA | NA | 0.04 (-0.93 to 1.01) | NA | 0.15 (-0.29 to 0.59) | 0.56 (-0.17 to 1.29) |
| --- | --- | --- | --- | --- | --- | --- | --- |
| -0.26 (-1.28 to 0.77) | Cognitive intervention  + Psychotherapy | NA | NA | 0.30 (-0.73 to 1.33) | NA | NA | NA |
| 0.64 (-0.27 to 1.56) | 0.90 (-0.47 to 2.28) | Cognitive intervention  + Psychotherapy + Exercise | NA | NA | -0.58 (-1.44 to 0.27) | NA | -0.08 (-0.64 to 0.48) |
| 0.36 (-0.73 to 1.46) | 0.62 (-0.88 to 2.12) | -0.28 (-1.27 to 0.71) | Exercise (Balance Group) | NA | NA | NA | 0.20 (-0.62 to 1.02) |
| 0.04 (-0.93 to 1.01) | 0.30 (-0.73 to 1.33) | -0.60 (-1.94 to 0.73) | -0.32 (-1.79 to 1.14) | Psychotherapy | NA | NA | NA |
| 0.11 (-0.99 to 1.20) | 0.36 (-1.14 to 1.87) | -0.54 (-1.35 to 0.27) | -0.26 (-1.41 to 0.90) | 0.06 (-1.40 to 1.53) | Psychotherapy + Exercise | NA | 0.40 (-0.48 to 1.28) |
| 0.15 (-0.29 to 0.59) | 0.41 (-0.71 to 1.52) | -0.49 (-1.51 to 0.52) | -0.21 (-1.40 to 0.97) | 0.11 (-0.96 to 1.17) | 0.04 (-1.14 to 1.23) | Usual care | NA |
| 0.56 (-0.17 to 1.29) | 0.82 (-0.44 to 2.08) | -0.08 (-0.64 to 0.48) | 0.20 (-0.62 to 1.02) | 0.52 (-0.69 to 1.73) | 0.46 (-0.36 to 1.28) | 0.41 (-0.44 to 1.27) | Wl |

**8.6 Attention**

| Active placebo | 0.01 (-0.69 to 0.71) | NA | NA | NA | NA | NA | NA | NA | NA | NA | -0.02 (-0.74 to 0.70) |
| --- | --- | --- | --- | --- | --- | --- | --- | --- | --- | --- | --- |
| -0.05(-0.68 to 0.57) | Cognitive intervention | -0.28 (-0.87 to 0.31) | -0.25 (-0.82 to 0.31) | NA | NA | NA | NA | NA | NA | 0.15 (-0.08 to 0.38) | 0.24 (-0.18 to 0.65) |
| -0.32(-1.13 to 0.49) | -0.27(-0.79 to 0.25) | Cognitive intervention  + Exercise | NA | NA | NA | NA | NA | NA | NA | 0.43 (-0.59 to 1.44) | NA |
| -0.10(-0.77 to 0.56) | -0.05(-0.38 to 0.28) | 0.22(-0.38 to 0.82) | Cognitive intervention  + Psychotherapy | NA | -0.47 (-1.38 to 0.44) | NA | NA | -0.61 (-1.53 to 0.30) | NA | 0.37 (-0.06 to 0.80) | 0.04 (-0.35 to 0.42) |
| 0.08(-0.84 to 1.00) | 0.13(-0.62 to 0.88) | 0.40(-0.51 to 1.31) | 0.18(-0.56 to 0.93) | Cognitive intervention  + Psychotherapy  + Exercise | NA | NA | NA | NA | -0.06 (-0.76 to 0.65) | NA | -0.03 (-0.70 to 0.64) |
| -0.37(-1.44 to 0.70) | -0.32(-1.21 to 0.57) | -0.05(-1.07 to 0.98) | -0.27(-1.12 to 0.59) | -0.45(-1.57 to 0.68) | Cognitive training | NA | NA | -0.14 (-0.99 to 0.70) | NA | NA | NA |
| 0.41(-0.48 to 1.31) | 0.47(-0.18 to 1.11) | 0.74(-0.07 to 1.55) | 0.52(-0.18 to 1.21) | 0.33(-0.64 to 1.31) | 0.78(-0.29 to 1.85) | Exercise (Aerobic Group) | -0.01 (-0.62 to 0.59) | NA | NA | -0.28 (-0.89 to 0.32) | NA |
| 0.40(-0.50 to 1.29) | 0.45(-0.19 to 1.10) | 0.72(-0.09 to 1.53) | 0.50(-0.19 to 1.19) | 0.32(-0.66 to 1.30) | 0.77(-0.31 to 1.84) | -0.01(-0.62 to 0.59) | Exercise (Balance Group) | NA | NA | -0.27 (-0.88 to 0.34) | NA |
| -0.36(-1.31 to 0.59) | -0.30(-1.05 to 0.44) | -0.03(-0.93 to 0.86) | -0.25(-0.97 to 0.46) | -0.44(-1.45 to 0.58) | 0.01(-0.80 to 0.82) | -0.77(-1.72 to 0.18) | -0.76(-1.70 to 0.19) | Psychotherapy | NA | -0.02 (-1.11 to 1.07) | NA |
| 0.02(-0.95 to 0.99) | 0.07(-0.73 to 0.88) | 0.34(-0.61 to 1.30) | 0.12(-0.68 to 0.93) | -0.06(-0.76 to 0.65) | 0.39(-0.77 to 1.55) | -0.39(-1.41 to 0.63) | -0.38(-1.40 to 0.64) | 0.38(-0.68 to 1.44) | Psychotherapy  + Exercise | NA | 0.03 (-0.71 to 0.76) |
| 0.13(-0.53 to 0.78) | 0.18(-0.04 to 0.40) | 0.45(-0.09 to 0.99) | 0.23(-0.10 to 0.57) | 0.05(-0.71 to 0.82) | 0.50(-0.39 to 1.38) | -0.28(-0.89 to 0.32) | -0.27(-0.88 to 0.34) | 0.49(-0.24 to 1.22) | 0.11(-0.71 to 0.93) | Usual care | NA |
| 0.05(-0.58 to 0.68) | 0.10(-0.23 to 0.44) | 0.37(-0.24 to 0.98) | 0.15(-0.17 to 0.47) | -0.03(-0.70 to 0.64) | 0.42(-0.48 to 1.32) | -0.36(-1.07 to 0.35) | -0.35(-1.06 to 0.36) | 0.41(-0.36 to 1.17) | 0.03(-0.71 to 0.76) | -0.08(-0.45 to 0.29) | Wl |

**8.7 Anxiety**

| Active placebo | 0.24 (-0.70 to 1.18) | NA | NA | NA | NA | NA | NA | NA | 0.09 (-0.86 to 1.05) |
| --- | --- | --- | --- | --- | --- | --- | --- | --- | --- |
| 0.20 (-0.66 to 1.07) | Cognitive intervention | 0.33 (-0.73 to 1.39) | 0.34 (-0.16 to 0.85) | NA | 0.67 (-0.43 to 1.77) | NA | NA | 0.20 (-0.47 to 0.86) | -0.18 (-0.79 to 0.44) |
| 0.53 (-0.83 to 1.90) | 0.33 (-0.73 to 1.39) | Cognitive intervention  + Occupational therapy | NA | NA | NA | NA | NA | NA | NA |
| 0.54 (-0.41 to 1.49) | 0.34 (-0.16 to 0.83) | 0.01 (-1.16 to 1.17) | Cognitive intervention  + Psychotherapy | NA | NA | NA | NA | -0.38 (-1.03 to 0.27) | -0.21 (-1.00 to 0.58) |
| 0.61 (-0.46 to 1.69) | 0.41 (-0.45 to 1.27) | 0.08 (-1.28 to 1.44) | 0.07 (-0.83 to 0.98) | Cognitive intervention  + Psychotherapy  + Exercise | NA | NA | -0.62 (-1.57 to 0.32) | NA | -0.48 (-1.11 to 0.15) |
| 0.87 (-0.52 to 2.27) | 0.67 (-0.43 to 1.77) | 0.34 (-1.18 to 1.86) | 0.33 (-0.87 to 1.53) | 0.26 (-1.13 to 1.65) | Exercise (Balance Group) | NA | NA | NA | NA |
| 0.36 (-0.93 to 1.65) | 0.15 (-0.82 to 1.13) | -0.17 (-1.62 to 1.27) | -0.18 (-1.15 to 0.79) | -0.26 (-1.53 to 1.01) | -0.52 (-1.98 to 0.95) | Psychotherapy | NA | -0.08 (-0.83 to 0.68) | NA |
| 0.19 (-1.06 to 1.44) | -0.01 (-1.08 to 1.06) | -0.34 (-1.84 to 1.17) | -0.34 (-1.45 to 0.77) | -0.42 (-1.31 to 0.47) | -0.68 (-2.21 to 0.86) | -0.16 (-1.58 to 1.26) | Psychotherapy  + Exercise | NA | -0.29 (-1.26 to 0.68) |
| 0.28 (-0.76 to 1.33) | 0.08 (-0.54 to 0.70) | -0.25 (-1.48 to 0.98) | -0.26 (-0.87 to 0.36) | -0.33 (-1.35 to 0.69) | -0.59 (-1.85 to 0.67) | -0.08 (-0.83 to 0.68) | 0.09 (-1.12 to 1.29) | Usual care | NA |
| 0.13 (-0.74 to 1.00) | -0.07 (-0.66 to 0.51) | -0.40 (-1.61 to 0.81) | -0.41 (-1.06 to 0.24) | -0.48 (-1.11 to 0.15) | -0.74 (-1.98 to 0.50) | -0.23 (-1.33 to 0.88) | -0.06 (-0.96 to 0.83) | -0.15 (-0.95 to 0.65) | Wl |

**8.8 Depression**

| Active placebo | 0.16 (-0.69 to 1.01) | NA | NA | NA | NA | NA | NA | NA | 0.06 (-0.80 to 0.92) |
| --- | --- | --- | --- | --- | --- | --- | --- | --- | --- |
| 0.21 (-0.58 to 1.01) | Cognitive intervention | 0.14 (-0.83 to 1.11) | 0.21 (-0.53 to 0.95) | NA | 0.44 (-0.57 to 1.45) | NA | NA | 0.57 (-0.17 to 1.31) | -0.21 (-0.83 to 0.41) |
| 0.35 (-0.90 to 1.61) | 0.14 (-0.83 to 1.11) | Cognitive intervention  + Occupational therapy | NA | NA | NA | NA | NA | NA | NA |
| 0.42 (-0.66 to 1.51) | 0.21 (-0.53 to 0.95) | 0.07 (-1.16 to 1.29) | Cognitive intervention  + Psychotherapy | NA | NA | NA | NA | 0.37 (-0.36 to 1.09) | NA |
| 0.18 (-0.80 to 1.15) | -0.04 (-0.87 to 0.80) | -0.18 (-1.46 to 1.10) | -0.25 (-1.36 to 0.87) | Cognitive intervention  + Psychotherapy  + Exercise | NA | NA | -0.40 (-1.26 to 0.45) |  | -0.17 (-0.73 to 0.39) |
| 0.66 (-0.63 to 1.94) | 0.44 (-0.57 to 1.45) | 0.30 (-1.10 to 1.70) | 0.23 (-1.02 to 1.48) | 0.48 (-0.83 to 1.79) | Exercise (Balance Group) | NA | NA | NA | NA |
| 0.98 (-0.28 to 2.23) | 0.76 (-0.21 to 1.74) | 0.62 (-0.76 to 2.00) | 0.56 (-0.41 to 1.52) | 0.80 (-0.48 to 2.08) | 0.32 (-1.08 to 1.72) | Psychotherapy | NA | -0.19 (-0.82 to 0.45) | NA |
| -0.03 (-1.18 to 1.11) | -0.25 (-1.27 to 0.78) | -0.39 (-1.80 to 1.02) | -0.45 (-1.72 to 0.81) | -0.21 (-1.01 to 0.59) | -0.69 (-2.12 to 0.75) | -1.01 (-2.42 to 0.41) | Psychotherapy  + Exercise | NA | -0.18 (-1.06 to 0.69) |
| 0.79 (-0.30 to 1.87) | 0.57 (-0.17 to 1.31) | 0.43 (-0.79 to 1.66) | 0.37 (-0.36 to 1.09) | 0.61 (-0.50 to 1.73) | 0.13 (-1.12 to 1.38) | -0.19 (-0.82 to 0.45) | 0.82 (-0.44 to 2.08) | Usual care | NA |
| 0.00 (-0.80 to 0.80) | -0.21 (-0.83 to 0.41) | -0.35 (-1.50 to 0.80) | -0.42 (-1.38 to 0.55) | -0.17 (-0.73 to 0.39) | -0.65 (-1.83 to 0.53) | -0.97 (-2.13 to 0.18) | 0.04 (-0.78 to 0.85) | -0.79 (-1.75 to 0.18) | Wl |

# eAppendix 9. Results of minimally contextualised framework

The minimally contextualised framework is the GRADE Working Group's guide for concluding a network meta-analysis [1]. The guidance uses a minimally contextualised approach that avoids value judgments regarding the magnitude of intervention effects. When grouping categories, it simultaneously considers the estimates of effect, the certainty of the evidence, and the rankings. As the first step, choosing reference intervention and decision threshold directly impact the minimally contextualised framework results. In this study, we used the waitlist intervention as the reference intervention and the 95% interval not intersecting the zero value as the decision threshold. The results are shown as follows:

**9.1. Subjective memory complaints**

| **Certainty of the evidence, and classification of intervention** | **Intervention*** | **Intervention VS Waitlist**  **(incremental standardized mean differences (95% credible interval)** | **Surface under the cumulative ranking curve** |
| --- | --- | --- | --- |
| **Low certainty (low to very low certainty evidence)** | | | |
| Category 1: might be inferior to the most effective or superior than the least effective | Exercise (Resistance Group) (L) | -0.78 (-1.37 to -0.18) | 0.888 |
|  | Exercise (Balance Group) (L) | -0.70 (-1.11 to -0.28) | 0.859 |
|  | Exercise (Aerobic Group) (L) | -0.68 (-1.27 to -0.09) | 0.832 |
|  | Cognitive intervention (VL) | -0.30 (-0.51 to -0.09) | 0.618 |
| Category 0: might be among the least effective | Active placebo (VL) | -0.18 (-0.56 to 0.19) | 0.490 |
|  | Waitlist | - | 0.310 |
|  | Cognitive intervention + psychotherapy (L) | 0.01 (-0.20 to 0.23) | 0.295 |
|  | Usual care (L) | 0.10 (-0.18 to 0.38) | 0.197 |
| Category -1: might be less effective | Psychotherapy (L) | 0.47 (0.06 to 0.88) | 0.011 |

*Letters in brackets represent the certainty of evidence for each intervention when compared with the reference: L=low; VL=very low.

**9.2. Global cognitive function**

| **Certainty of the evidence, and classification of intervention** | **Intervention*** | **Intervention VS Waitlist**  **(mean difference (95% credible interval)** | **Surface under the cumulative ranking curve** |
| --- | --- | --- | --- |
| **Low certainty (low to very low certainty evidence)** | | | |
| Category 0: might be among the least effective | Cognitive intervention + Psychotherapy + Exercise (L) | 0.38 (-0.50 to 1.27) | 0.761 |
|  | Psychotherapy + Exercise (L) | 0.09 (-1.13 to 1.31) | 0.593 |
|  | Cognitive intervention + Psychotherapy (L) | 0.06 (-0.93 to 1.04) | 0.628 |
|  | Waitlist | - | 0.563 |
|  | Psychotherapy (L) | -0.01 (-1.58 to 1.56) | 0.563 |
|  | Exercise (Balance Group) (L) | -0.03 (-1.08 to 1.01) | 0.560 |
|  | Cognitive intervention + Occupational therapy (L) | -0.05 (-1.85 to 1.75) | 0.541 |
|  | Cognitive intervention (L) | -0.27 (-1.44 to 0.90) | 0.416 |
|  | Cognitive intervention + Exercise (VL) | -0.28 (-1.59 to 1.03) | 0.424 |
|  | Exercise (Aerobic Group) (L) | -0.41 (-1.83 to 1.01) | 0.358 |
|  | Usual care (L) | -0.85 (-1.94 to 0.25) | 0.094 |

*Letters in brackets represent the certainty of evidence for each intervention when compared with the reference: L=low; VL=very low.

**9.3. Language function**

| **Certainty of the evidence, and classification of intervention** | **Intervention*** | **Intervention VS Waitlist**  **(mean difference (95% credible interval)** | **Surface under the cumulative ranking curve** |
| --- | --- | --- | --- |
| **Low certainty (low to very low certainty evidence)** | | | |
| Category 0: might be among the least effective | Cognitive intervention (L) | 0.47 (-0.01 to 0.96) | 0.829 |
|  | Cognitive intervention + Psychotherapy + Exercise (L) | 0.36 (-0.24 to 0.96) | 0.656 |
|  | Usual care (L) | 0.36 (-0.21 to 0.93) | 0.638 |
|  | Cognitive intervention + Psychotherapy (L) | 0.15 (-0.22 to 0.52) | 0.416 |
|  | Waitlist | - | 0.200 |
|  | Exercise + Psychotherapy (VL) | -0.02 (-0.67 to 0.63) | 0.262 |

*Letters in brackets represent the certainty of evidence for each intervention when compared with the reference: L=low; VL=very low.

**9.4. Executive function**

| **Certainty of the evidence, and classification of intervention** | **Intervention*** | **Intervention VS Waitlist**  **(mean difference (95% credible interval)** | **Surface under the cumulative ranking curve** |
| --- | --- | --- | --- |
| **Low certainty (low to very low certainty evidence)** | | | |
| Category 1: might be inferior to the most effective or superior than the least effective | Psychotherapy + Exercise (VL) | 0.72 (0.28 to 1.16) | 0.942 |
|  | Cognitive intervention + Psychotherapy + Exercise (L) | 0.55 (0.17 to 0.94) | 0.857 |
| Category 0: might be among the least effective | Exercise (Balance Group) (VL) | 0.16 (-0.45 to 0.77) | 0.557 |
|  | Cognitive intervention (VL) | 0.15 (-0.26 to 0.57) | 0.561 |
|  | Psychotherapy (VL) | 0.11 (-0.65 to 0.88) | 0.513 |
|  | Cognitive intervention + Exercise (VL) | 0.10 (-0.57 to 0.77) | 0.499 |
|  | Cognitive intervention + Psychotherapy (L) | 0.09 (-0.15 to 0.33) | 0.500 |
|  | Waitlist | - | 0.380 |
|  | Usual care (VL) | -0.48 (-1.01 to 0.04) | 0.092 |
|  | Active placebo (VL) | -0.55 (-1.32 to 0.23) | 0.099 |

*Letters in brackets represent the certainty of evidence for each intervention when compared with the reference: L=low; VL=very low.

**9.5. Visuospatial ability**

| **Certainty of the evidence, and classification of intervention** | **Intervention*** | **Intervention VS Waitlist**  **(mean difference (95% credible interval)** | **Surface under the cumulative ranking curve** |
| --- | --- | --- | --- |
| **Low certainty (low to very low certainty evidence)** | | | |
| Category 0: might be among the least effective | Cognitive intervention + Psychotherapy (VL) | 0.82 (-0.44 to 2.08) | 0.778 |
|  | Cognitive intervention (VL) | 0.56 (-0.17 to 1.29) | 0.680 |
|  | Psychotherapy (VL) | 0.52 (-0.69 to 1.73) | 0.591 |
|  | Psychotherapy + Exercise (L) | 0.46 (-0.36 to 1.28) | 0.596 |
|  | Usual care (VL) | 0.41 (-0.44 to 1.27) | 0.526 |
|  | Exercise (Balance Group) (L) | 0.20 (-0.62 to 1.02) | 0.413 |
|  | Waitlist | - | 0.229 |
|  | Cognitive intervention + Psychotherapy + Exercise (L) | -0.08 (-0.64 to 0.48) | 0.188 |

*Letters in brackets represent the certainty of evidence for each intervention when compared with the reference: L=low; VL=very low.

**9.6. Attention**

| **Certainty of the evidence, and classification of intervention** | **Intervention*** | **Intervention VS Waitlist**  **(mean difference (95% credible interval)** | **Surface under the cumulative ranking curve** |
| --- | --- | --- | --- |
| **High certainty (moderate to high certainty evidence)** | | | |
|  | Cognitive intervention + Psychotherapy (M) | 0.15(-0.17 to 0.47) | 0.626 |
| **Low certainty (low to very low certainty evidence)** | | | |
| Category 0: might be among the least effective | Cognitive training (L) | 0.42(-0.48 to 1.32) | 0.570 |
|  | Psychotherapy (VL) | 0.41(-0.36 to 1.17) | 0.776 |
|  | Cognitive intervention + Exercise (L) | 0.37(-0.24 to 0.98) | 0.787 |
|  | Cognitive intervention | 0.10(-0.23 to 0.44) | 0.570 |
|  | Active placebo (VL) | 0.05(-0.58 to 0.68) | 0.494 |
|  | Psychotherapy + Exercise (L) | 0.03(-0.71 to 0.76) | 0.479 |
|  | Waitlist | - | 0.425 |
|  | Cognitive intervention + Psychotherapy + Exercise (L) | -0.03(-0.70 to 0.64) | 0.425 |
|  | Usual care (VL) | -0.08(-0.45 to 0.29) | 0.326 |
|  | Exercise (Balance Group) (VL) | -0.35(-1.06 to 0.36) | 0.173 |
|  | Exercise (Aerobic Group) (VL) | -0.36(-1.07 to 0.35) | 0.164 |

*Letters in brackets represent the certainty of evidence for each intervention when compared with the reference: M=moderate; L=low; VL=very low.

**9.7. Anxiety**

| **Certainty of the evidence, and classification of intervention** | **Intervention*** | **Intervention VS Waitlist**  **(mean difference (95% credible interval)** | **Surface under the cumulative ranking curve** |
| --- | --- | --- | --- |
| **Low certainty (low to very low certainty evidence)** | | | |
| Category 0: might be among the least effective | Active placebo (VL) | 0.13 (-0.74 to 1.00) | 0.747 |
|  | Waitlist | - | 0.698 |
|  | Psychotherapy + Exercise (L) | -0.06 (-0.96 to 0.83) | 0.611 |
|  | Cognitive intervention (L) | -0.07 (-0.66 to 0.51) | 0.643 |
|  | Usual care (L) | -0.15 (-0.95 to 0.65) | 0.565 |
|  | Psychotherapy (L) | -0.23 (-1.33 to 0.88) | 0.500 |
|  | Cognitive intervention + Occupational therapy (L) | -0.40 (-1.61 to 0.81) | 0.395 |
|  | Cognitive intervention + Psychotherapy (L) | -0.41 (-1.06 to 0.24) | 0.326 |
|  | Cognitive intervention + Psychotherapy + Exercise (L) | -0.48 (-1.11 to 0.15) | 0.299 |
|  | Exercise (Balance Group) (L) | -0.74 (-1.98 to 0.50) | 0.216 |

*Letters in brackets represent the certainty of evidence for each intervention when compared with the reference: L=low; VL=very low.

**9.8. Depression**

| **Certainty of the evidence, and classification of intervention** | **Intervention*** | **Intervention VS Waitlist**  **(mean difference (95% credible interval)** | **Surface under the cumulative ranking curve** |
| --- | --- | --- | --- |
| **Low certainty (low to very low certainty evidence)** | | | |
| Category 0: might be among the least effective | Psychotherapy + Exercise (L) | 0.04 (-0.78 to 0.85) | 0.727 |
|  | Waitlist | - | 0.747 |
|  | Active placebo (VL) | 0.00 (-0.80 to 0.80) | 0.723 |
|  | Cognitive intervention + Psychotherapy + Exercise (L) | -0.17 (-0.73 to 0.39) | 0.585 |
|  | Cognitive intervention (VL) | -0.21 (-0.83 to 0.41) | 0.592 |
|  | Cognitive intervention + Occupational therapy (VL) | -0.35 (-1.50 to 0.80) | 0.491 |
|  | Cognitive intervention + Psychotherapy (L) | -0.42 (-1.38 to 0.55) | 0.455 |
|  | Exercise (Balance Group) (VL) | -0.65 (-1.83 to 0.53) | 0.317 |
|  | Usual care (L) | -0.79 (-1.75 to 0.18) | 0.220 |
|  | Psychotherapy (L) | -0.97 (-2.13 to 0.18) | 0.144 |

*Letters in brackets represent the certainty of evidence for each intervention when compared with the reference: L=low; VL=very low.

Reference:

1. Brignardello-Petersen R, Florez ID, Izcovich A, Santesso N, Hazlewood G, Alhazanni W, et al. GRADE approach to drawing conclusions from a network meta-analysis using a minimally contextualised framework. Bmj. 2020;371:m3900.

# eAppendix 10. Assessment of transitivity: distribution of effect modifiers

Transitivity is a crucial assumption for the robustness of indirect comparisons that consist of direct comparisons. Thus, we compared the distributions of the characteristics across arms grouped by the corresponding interventions. We considered the following factors: age, gender, and treatment duration. The transmissibility analysis results suggested that the intervention duration was higher in the exercise (Aerobic Group) + exercise (Resistance Group) group than in the other intervention subgroups, which may influence the effect size of indirect evidence to be biased. The other moderators, including age and gender, appeared to be evenly distributed across the subgroups.

**10.1 Age**

**
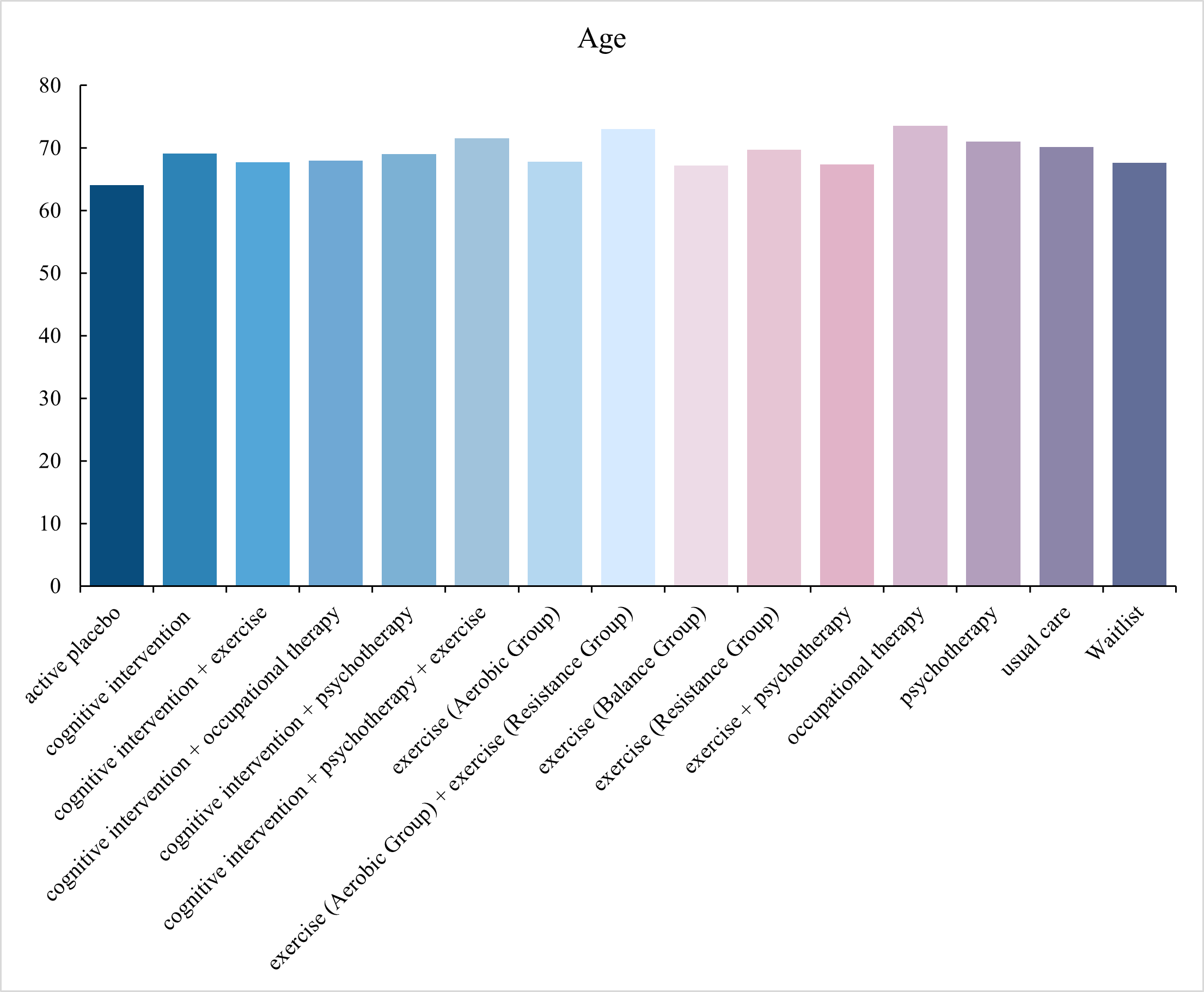
**

**10.2 Gender**

**
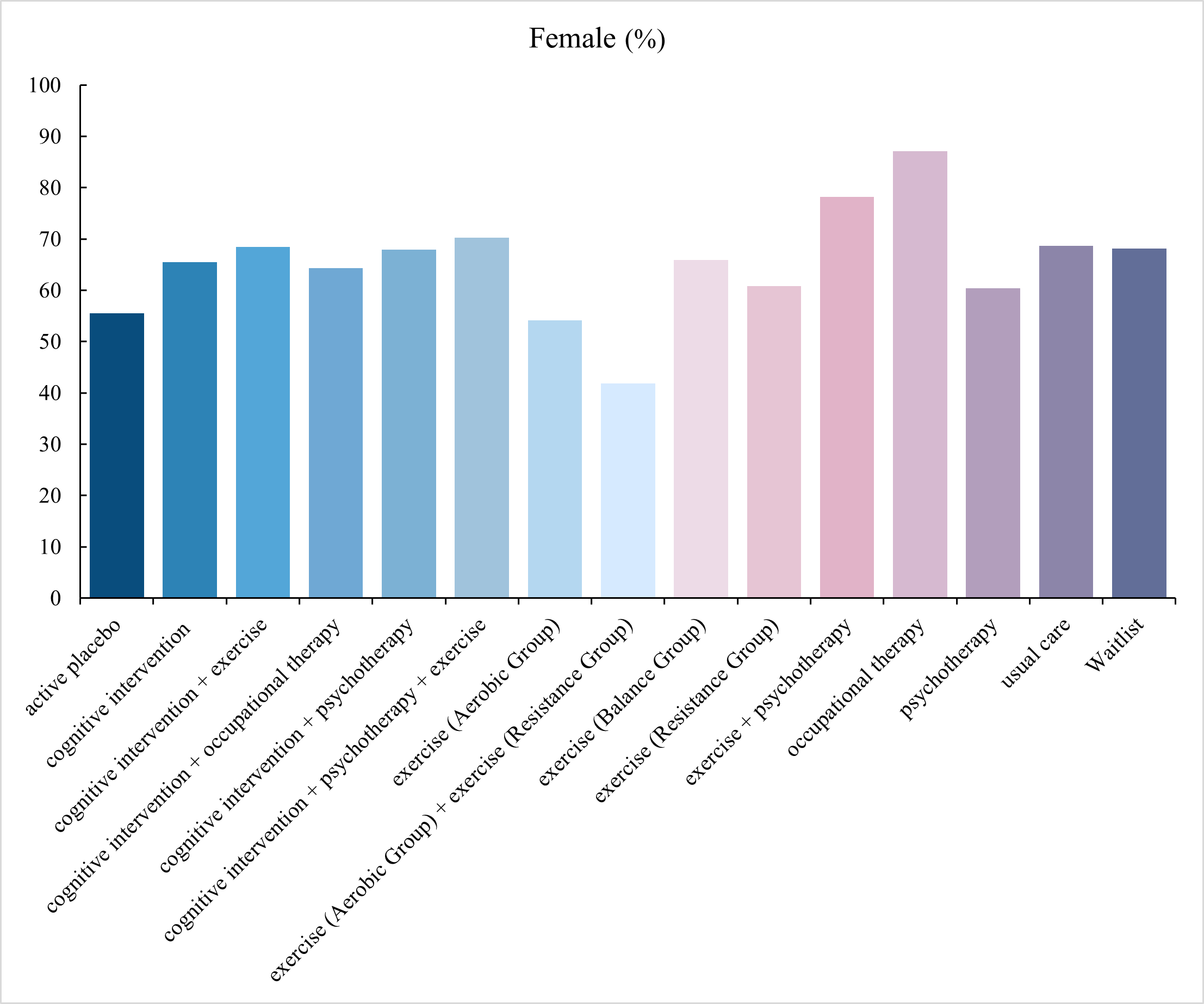
**

**10.3 Treatment duration**

**
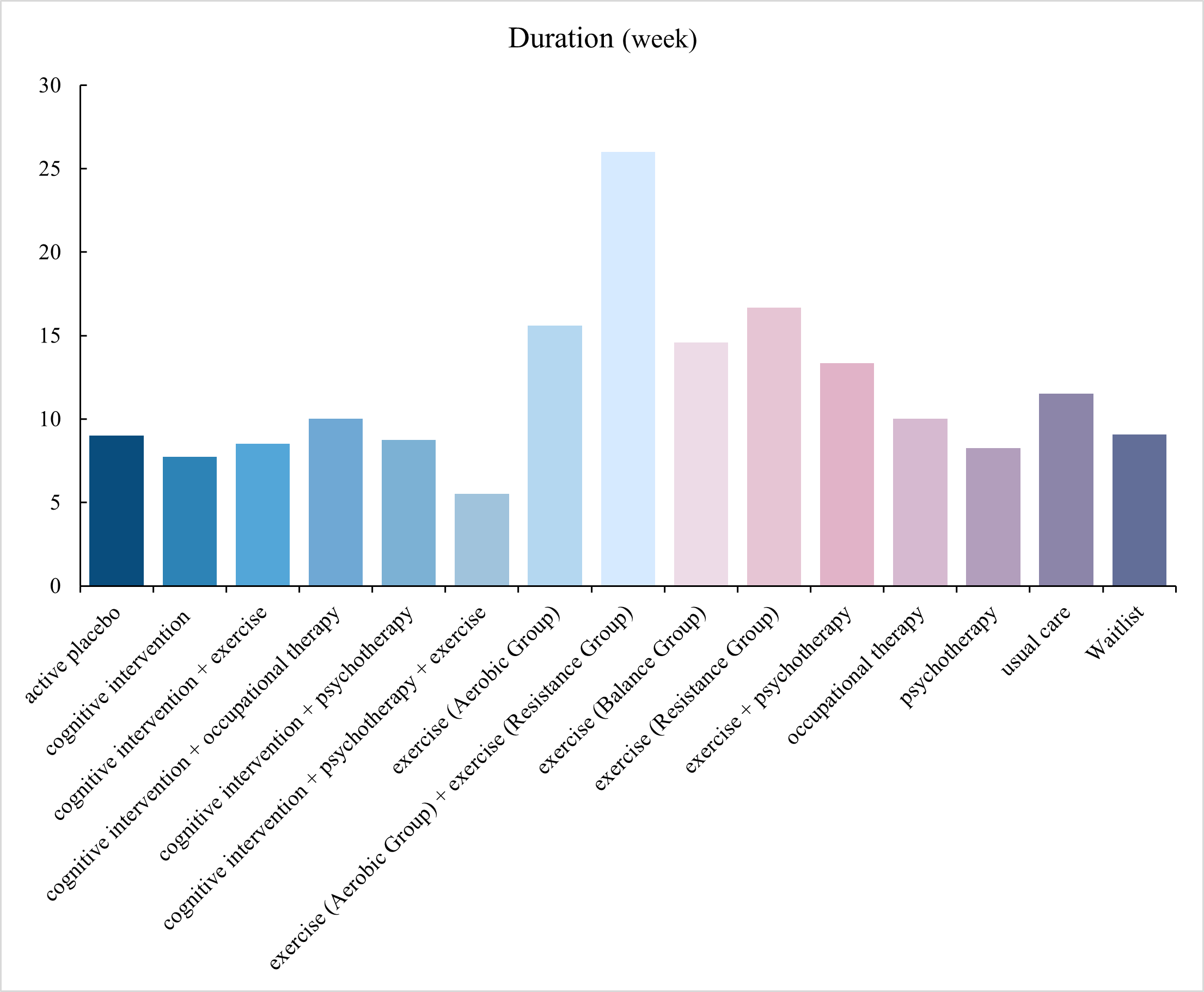
**

# eAppendix 11. Inconsistency test for direct and indirect evidence


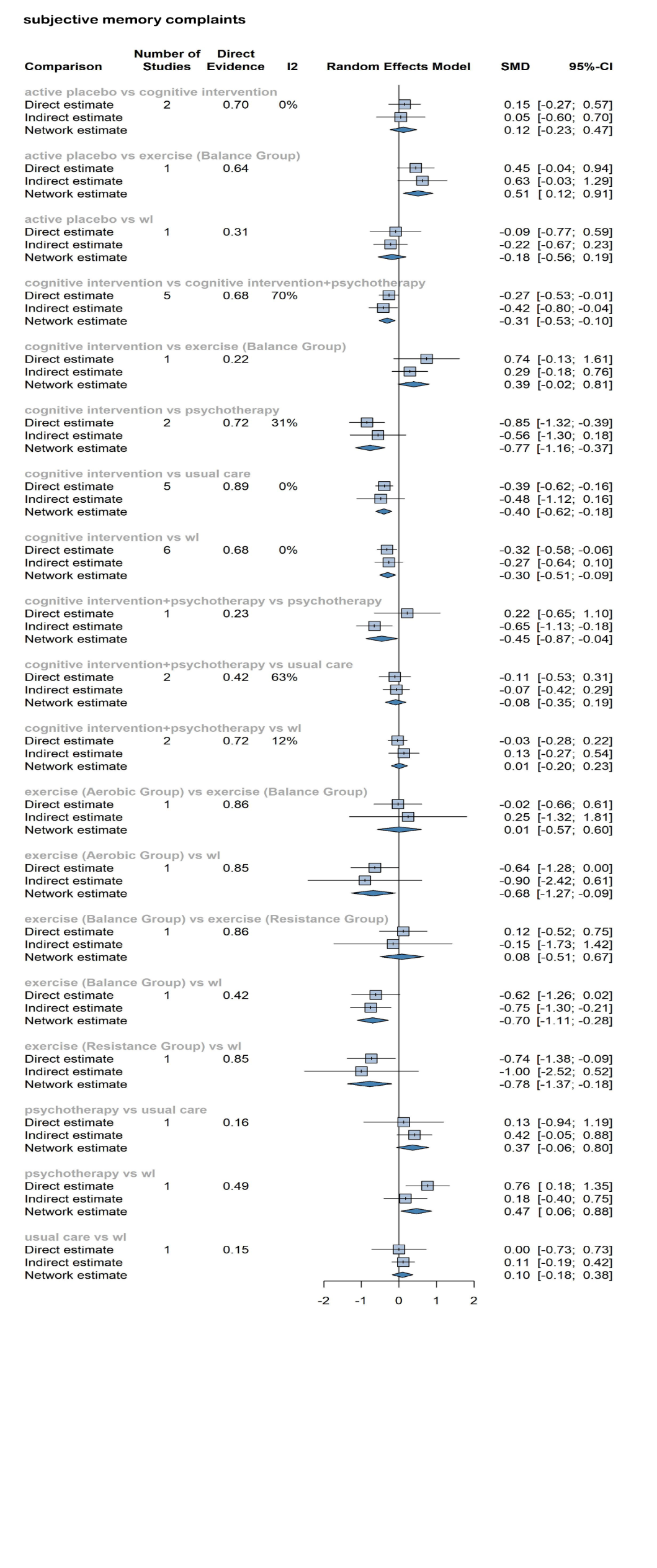


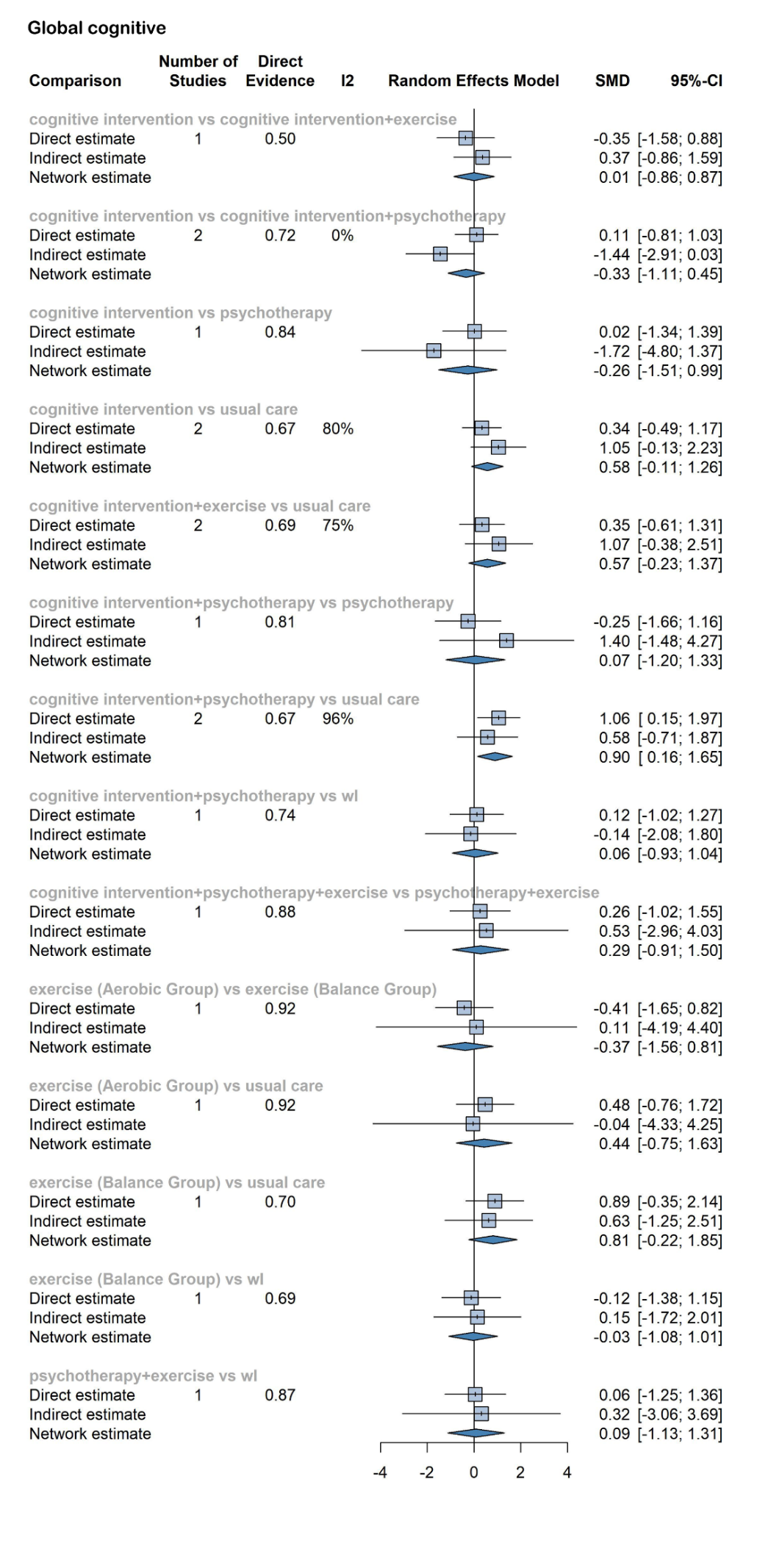


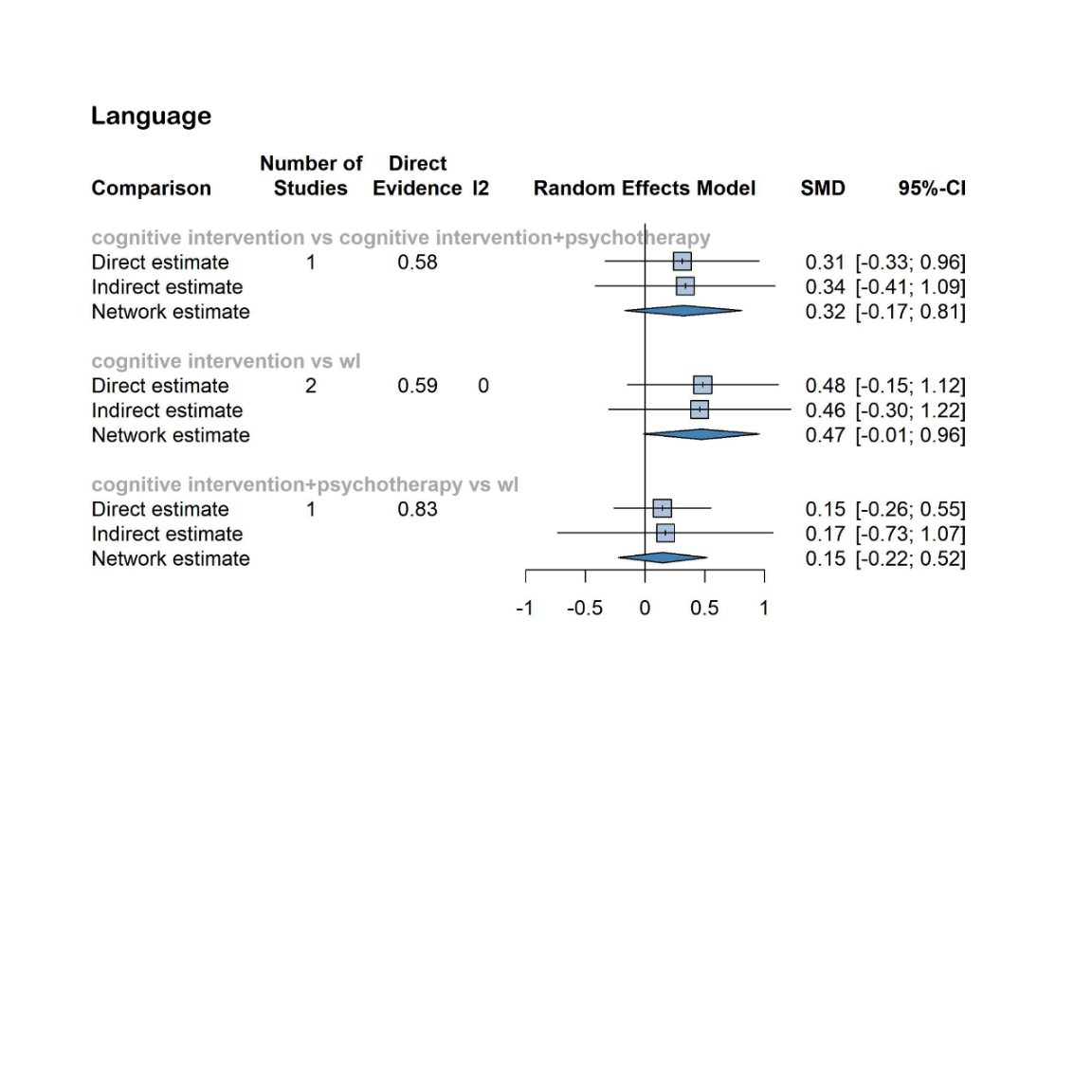


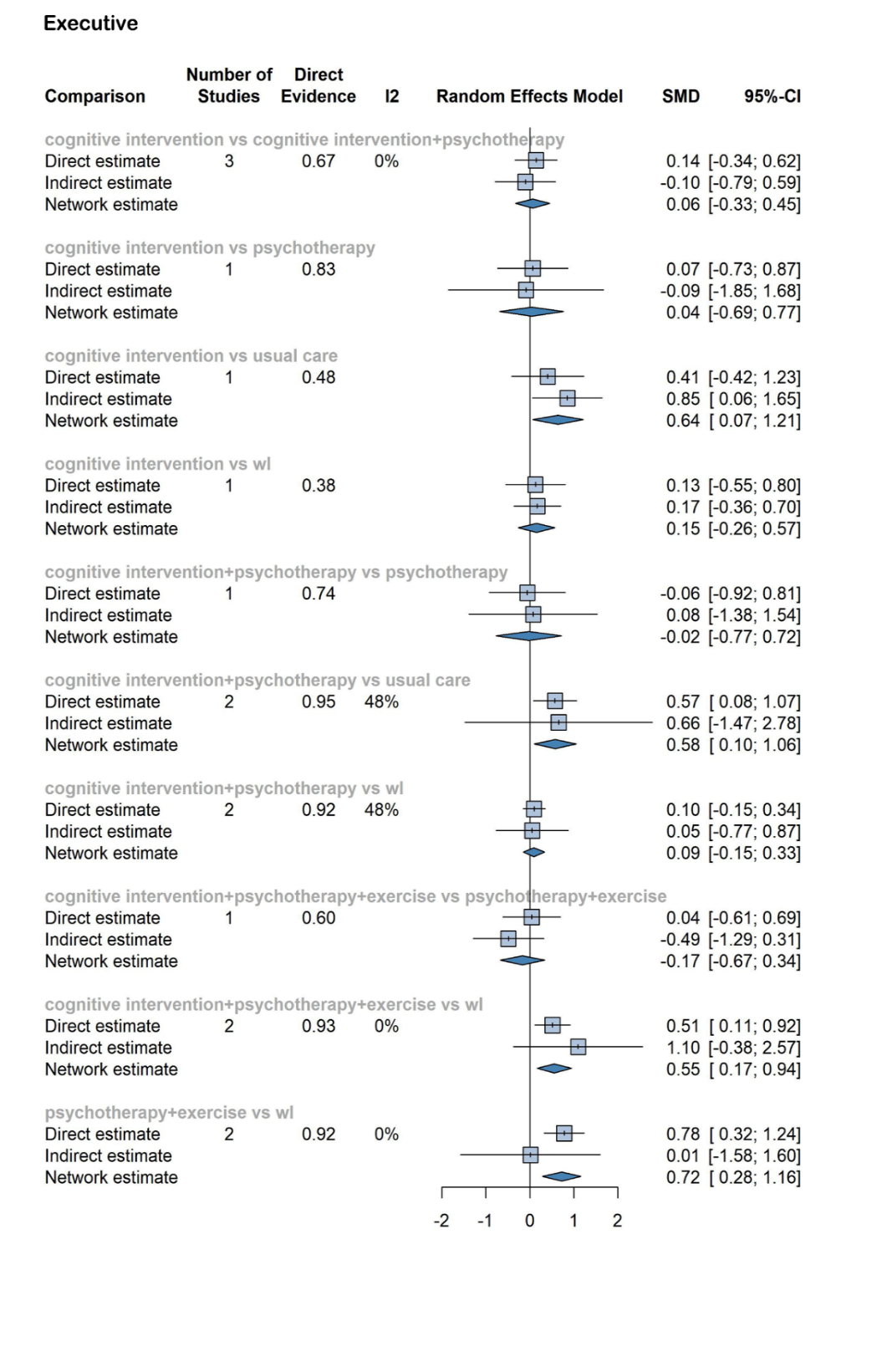


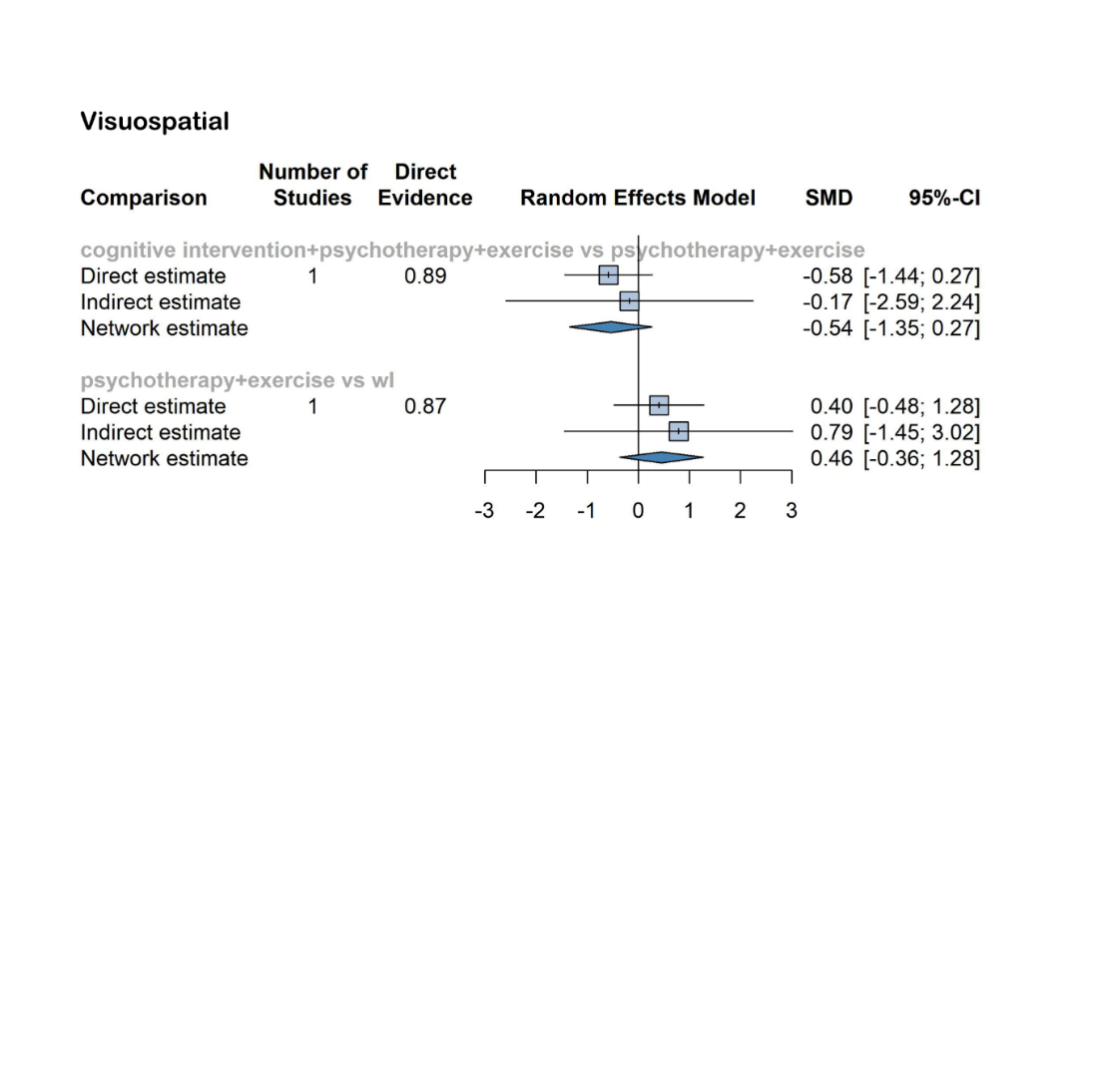


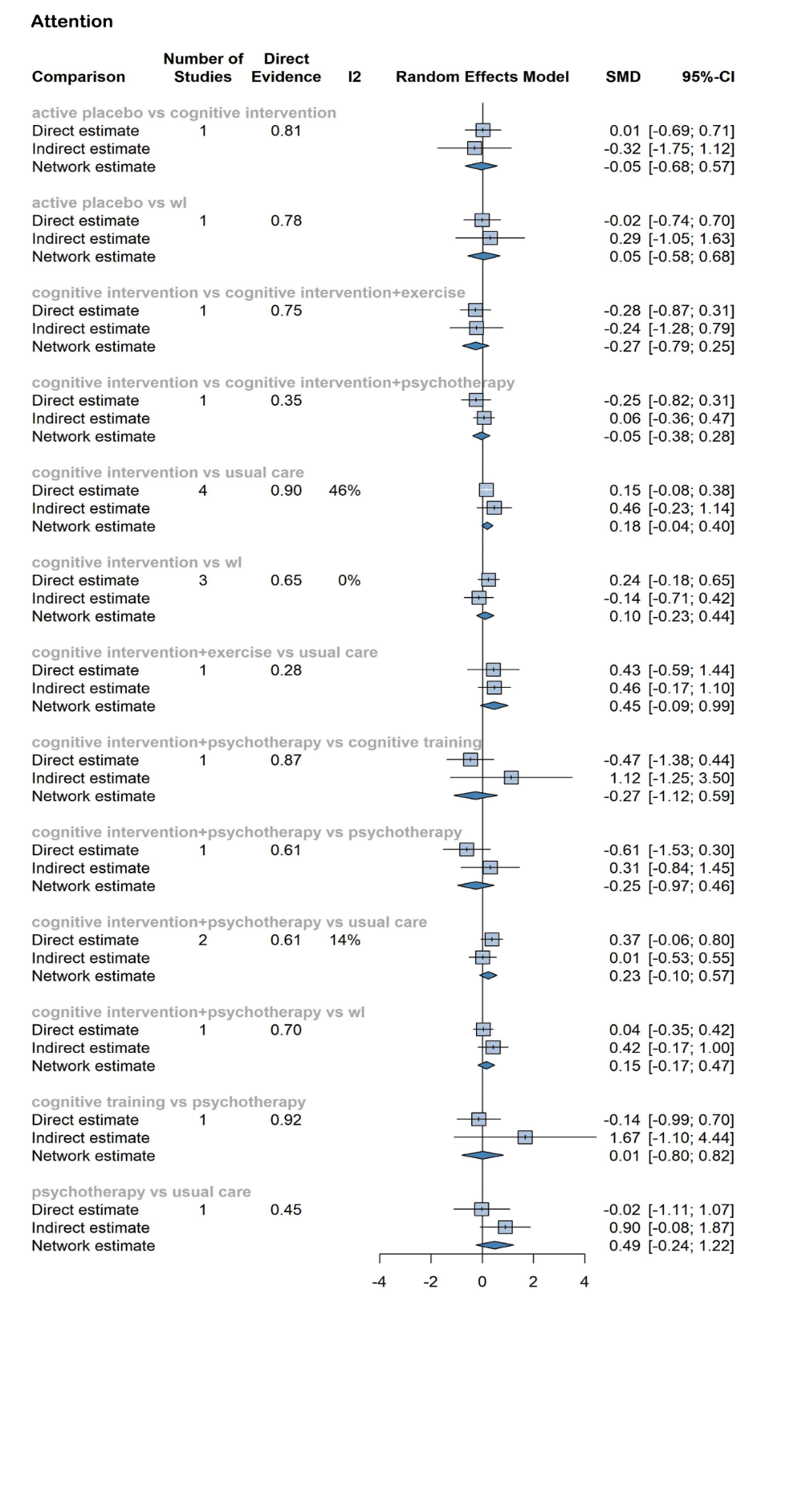


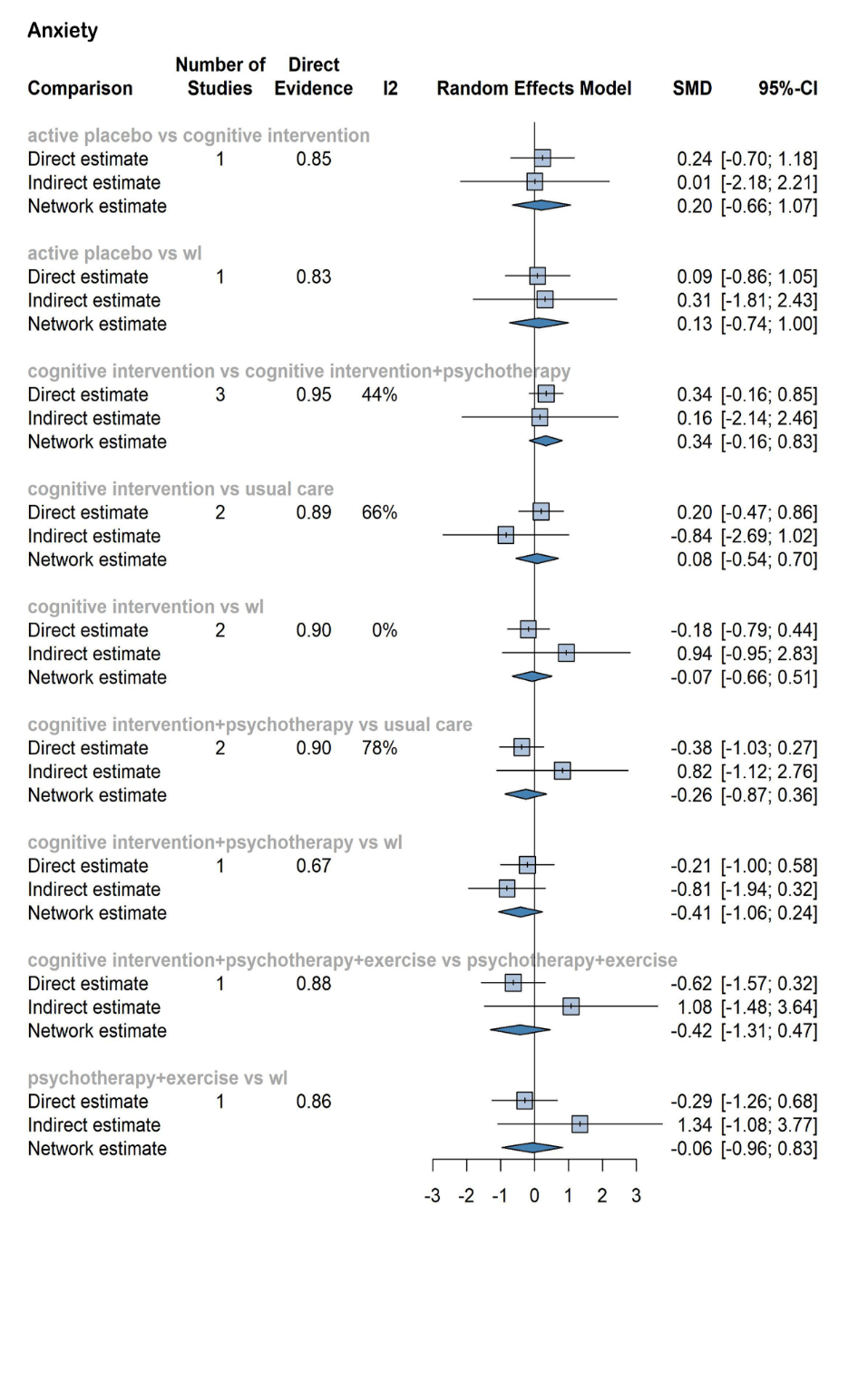


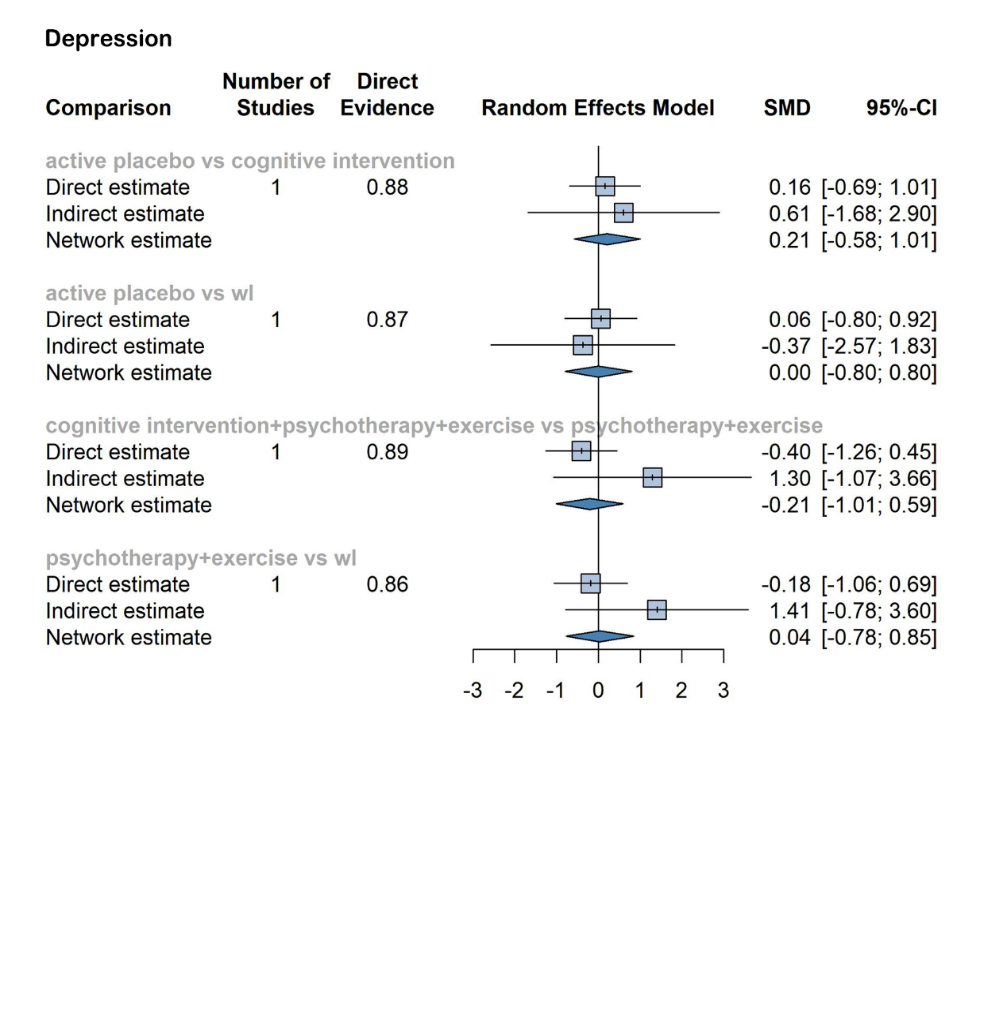


# eAppendix 12. Grading the evidence of the network meta-analysis using CINeMA

## 12.1 CINeMA quality assessment of the comparisons in the network

We evaluated the certainty of evidence for all the outcomes by using the Confidence in Network Meta-Analysis Software CINeMA, which covers 6 domains: (i) within-study bias, (ii) reporting bias, (iii) indirectness, (iv) imprecision, (v) heterogeneity, and (vi) incoherence and was considered to be a transparent, rigorous, and comprehensive system. We assessed each network estimate according to the following criteria [1-2].

1.within-study bias:

We assigned to each trial an overall risk of bias judgement according to the following criteria: a study with a judgement of high risk of bias in one or more domain has been considered as a ‘high risk’ study, a judgement of low risk of bias in most domains lead to a ‘low risk’ study at least four out of seven, and a study with judgements of unclear risk of bias in most domains has been considered as a ‘moderate risk’ study at least four out of seven. We rated each pairwise comparison based on the average bias judgment across direct estimate contribution from individual studies to the contribution matrix. The table below shows how the global rating is coded base on the individual items of the risk of bias tool:

| Global rating | Items with high risk | Items with unclear risk |
| --- | --- | --- |
| low | 0 | ≤ 3 |
| moderate | 0 | ＞3 |
| moderate | 1 | any |
| High risk | ≥ 1 | any |

2. Reporting bias:

We systematically searched electronic databases and contacted investigators and relevant experimenters by mail to obtain detailed literature information for incomplete reports and information on unpublished experiments. Also, we conducted various manual and non-database Internet searches to find additional papers that met the criteria. Therefore, although our study took a lot of time to complete the review, for which recently published literature was not included, our search was systematic and comprehensive for the dates included in the search. Furthermore, in pairwise comparisons with at least ten trials, the presence of small-study effects was examined graphically by generating comparison adjusted funnel plots. Egger’s test was carried out to evaluate the funnel plot symmetry. A p-value less than 0.05 was considered significant [3]. We did not find that there was a significant publication bias.

3. Indirectness:

In order to control for transmission studies in adults with subjective memory complaints, studies in cognitively impaired subjects (MCI, AD), psychiatric disorders, neurologic disorders, or substance abuse were excluded a priori. Because their degree of cognitive impairment and performance is known to be different from that of SCD adults. Therefore, no indirectness was assumed and no comparison was downgraded for this reason.

4. Imprecision:

We could not find any published value for clinically important relative effect estimate in the intervention study of SCD. Drawing from Cohen et al. effect sizes were categorized as small when below 0.2, moderate between 0.2 and 0.5, and large above 0.5 [4]. Through group discussions, for comparisons of two non-pharmacological interventions the clinically meaningful threshold was set at standardized mean differences of -0.5 and 0.5 for continuous outcomes and at odds ratio of 0.8 and 1.25 for dichotomous outcomes. If the confidence interval crossed one threshold the comparison was downgraded one level. Crossing both thresholds resulted in a downgrading of two levels.

5. Heterogeneity:

We made use of prediction intervals to prepopulate judgments on heterogeneity and its implications on the quality of the network treatment effects. We judged the agreement of conclusions based on confidence and prediction intervals in relation to the clinically important effect size. The recommendations automatically provided by CINeMA were followed [2].

6. Incoherence:

To evaluate the presence of inconsistency locally, we compared direct and indirect treatment estimates of each treatment comparisons as this can be used to inspect the consistency of a network meta-analysis [5]. For this purpose, we used the netsplit command in the R package netmeta 1.0-1, which enables the splitting of the network evidence into direct and indirect contributions [6]. For each treatment comparison, direct and indirect treatment estimates and the network are presented as forest plots. We did not downgrade comparisons that had only direct evidence with respect to incoherence.

**Reference:**

1. Nikolakopoulou A, Higgins JPT, Papakonstantinou T, Chaimani A, Del Giovane C, Egger M, et al. CINeMA: An approach for assessing confidence in the results of a network meta-analysis. PLoS Med. 2020;17(4):e1003082.
2. De Crescenzo F, D'Alò GL, Ostinelli EG, Ciabattini M, Di Franco V, Watanabe N, et al. Comparative effects of pharmacological interventions for the acute and long-term management of insomnia disorder in adults: a systematic review and network meta-analysis. Lancet. 2022;400(10347):170-84.
3. Sterne JAC, Egger M, Moher D. Addressing Reporting Biases. In: Higgins JPT, editor. Cochrane handbook for systematic reviews of interventions. Chichester: Wiley-Blackwell; 2008. p. 297–333.
4. Warsi A, LaValley MP, Wang PS, Avorn J, Solomon DH. Arthritis self-management education programs: a meta-analysis of the effect on pain and disability. Arthritis Rheum. 2003;48(8):2207-13.
5. Dias S, Welton NJ, Caldwell DM, Ades AE. Checking consistency in mixed treatment comparison meta-analysis. Stat Med. 2010;29(7-8):932-44.
6. Roheger M, Hennersdorf XS, Riemann S, Flöel A, Meinzer M. A systematic review and network meta-analysis of interventions for subjective cognitive decline. Alzheimers Dement (N Y). 2021;7(1):e12180.

## 12.2 Results of the comparison-adjusted funnel plot

**12.2.1 Subjective memory complaints**


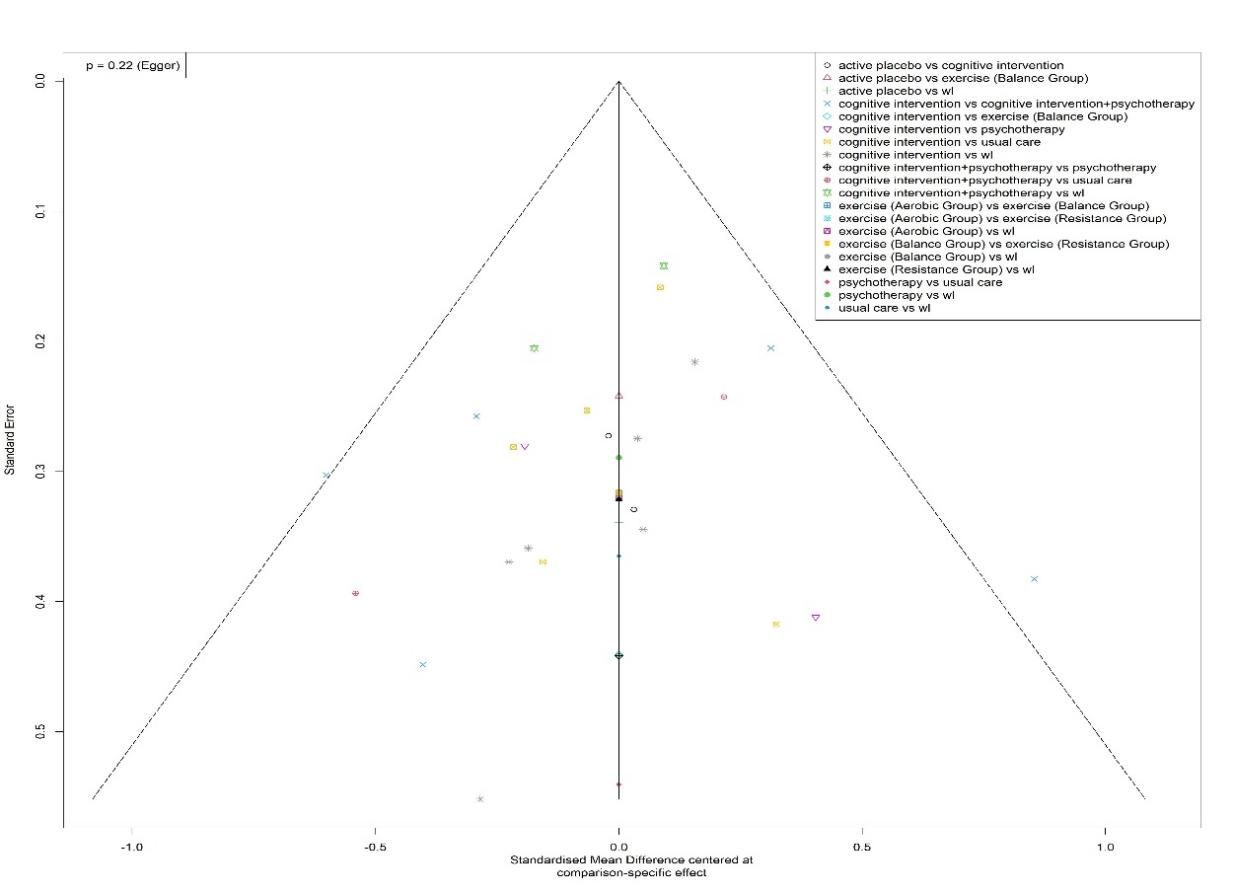


**12.2.2 General Cognitive Functioning**


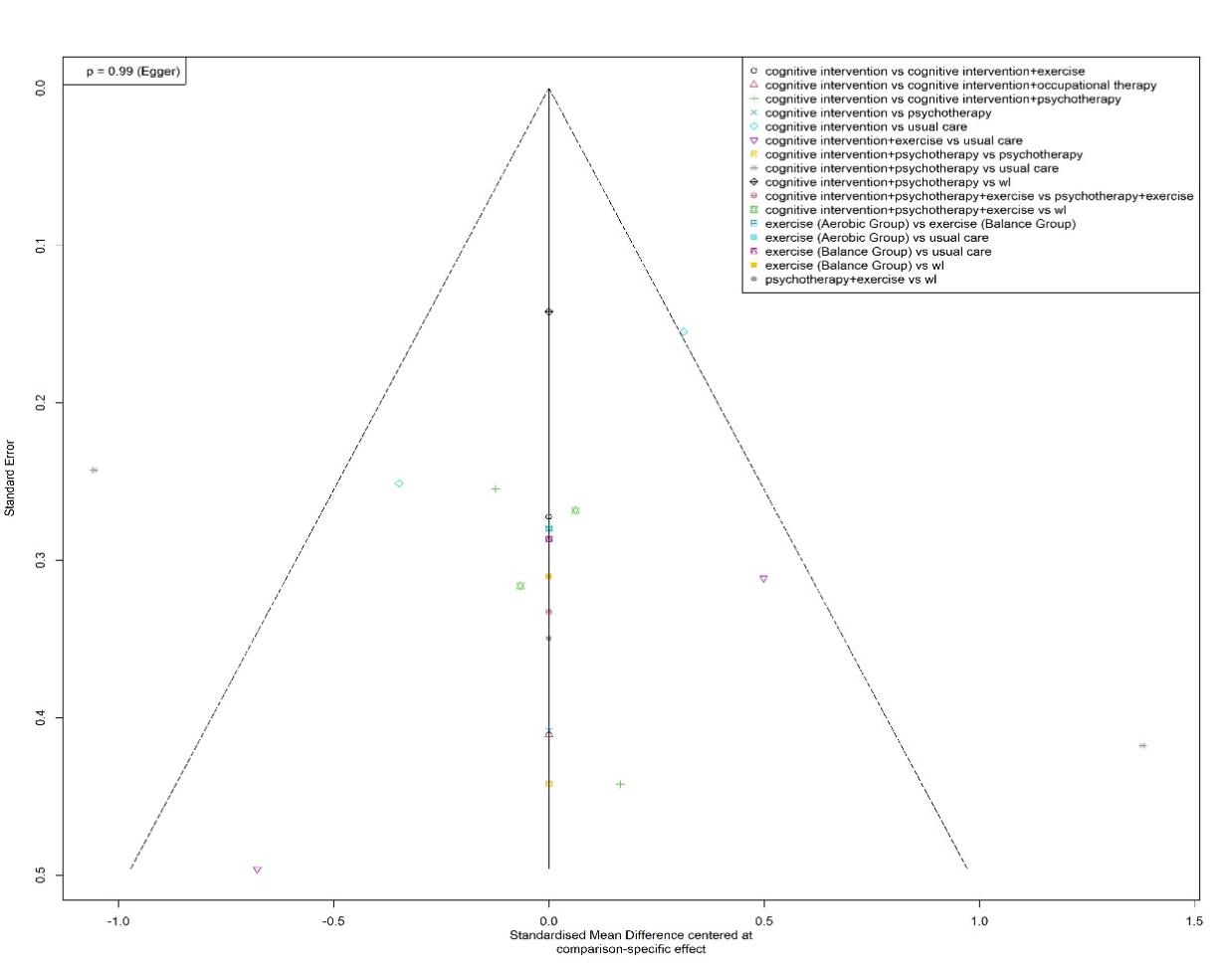


**12.2.3 Language function: less than ten studies, not suitable for publication bias test**

**12.2.4 Executive function**


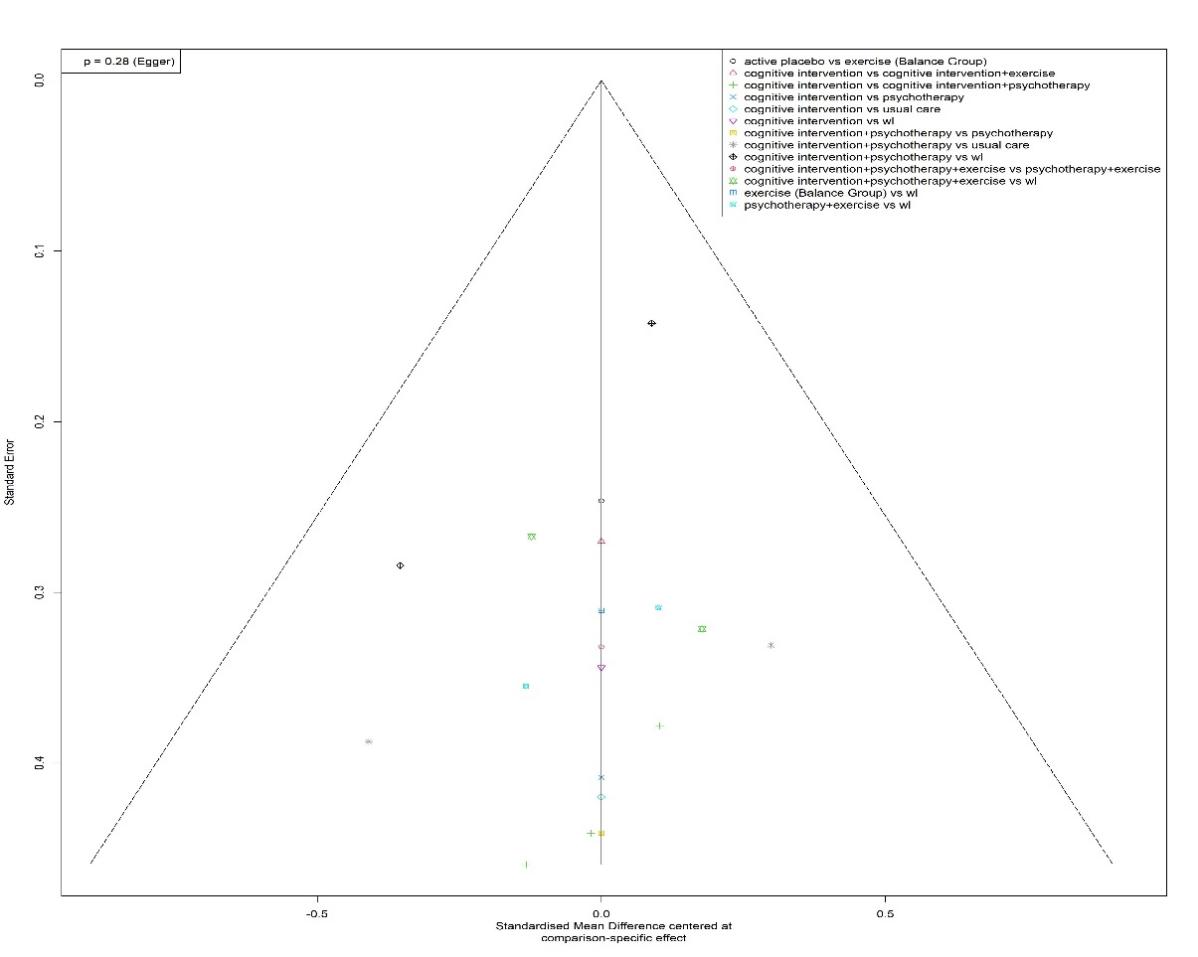


**12.2.5 Visuospatial ability**


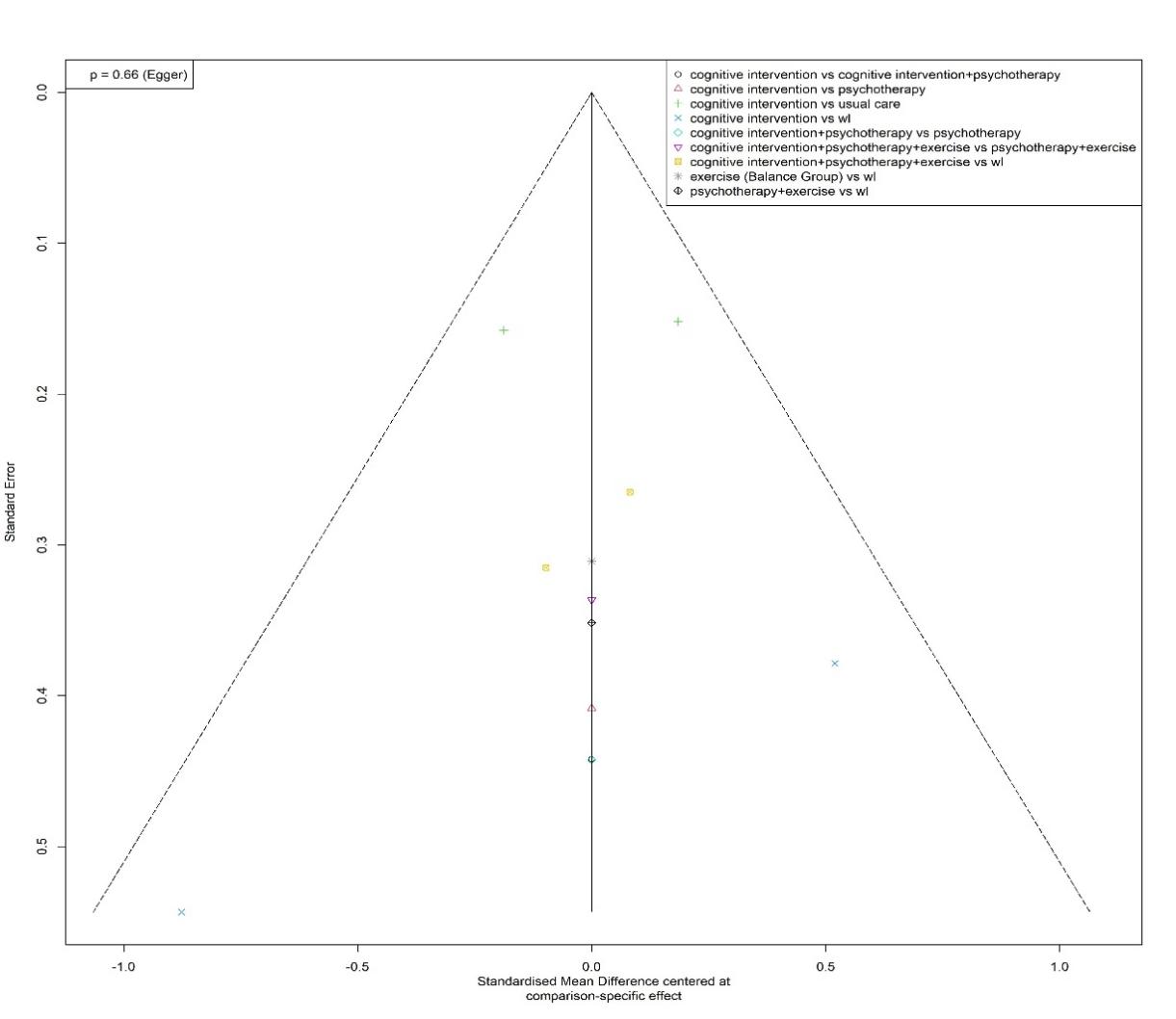


**12.2.6 Attention**


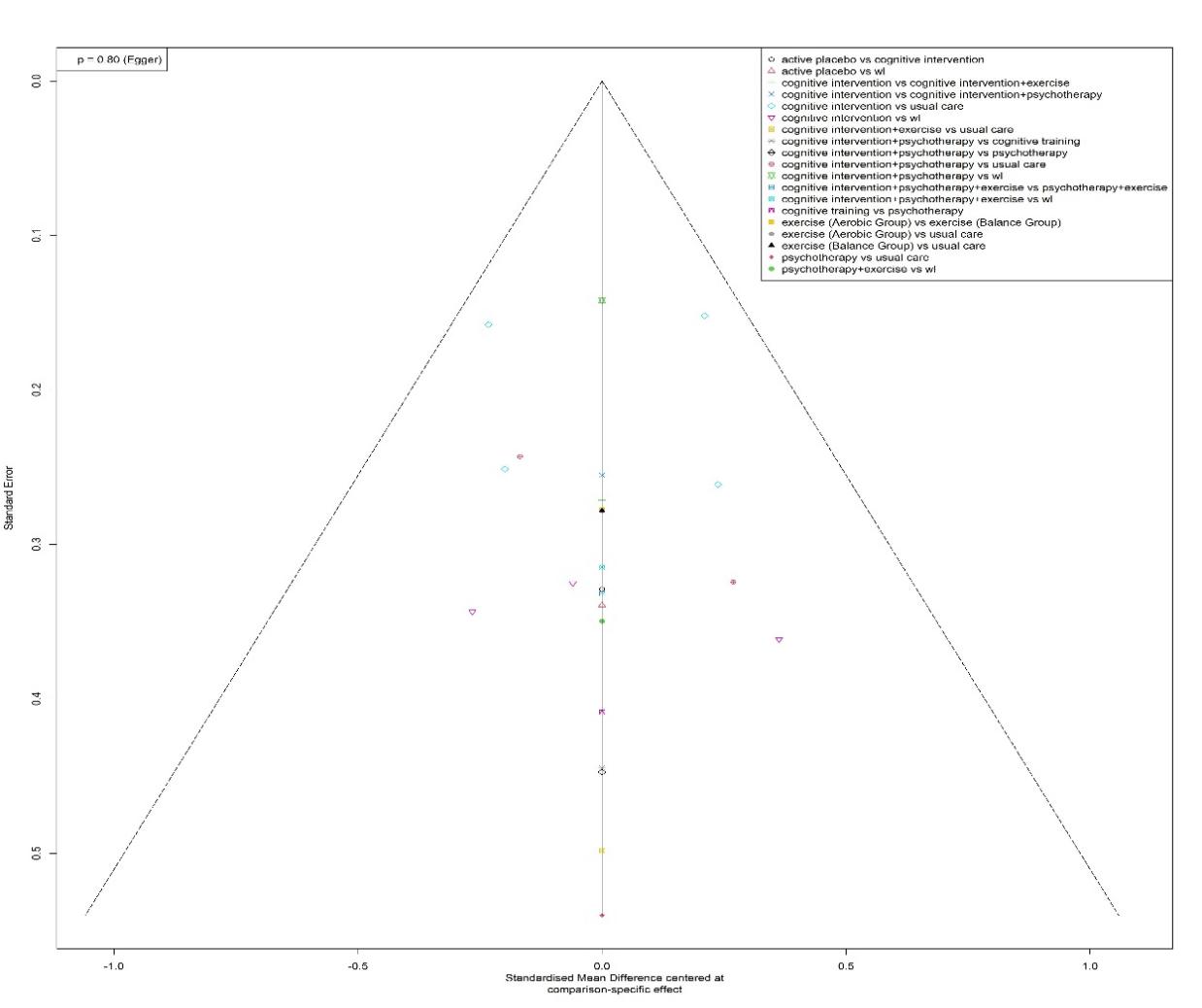


**12.2.7 Anxiety**


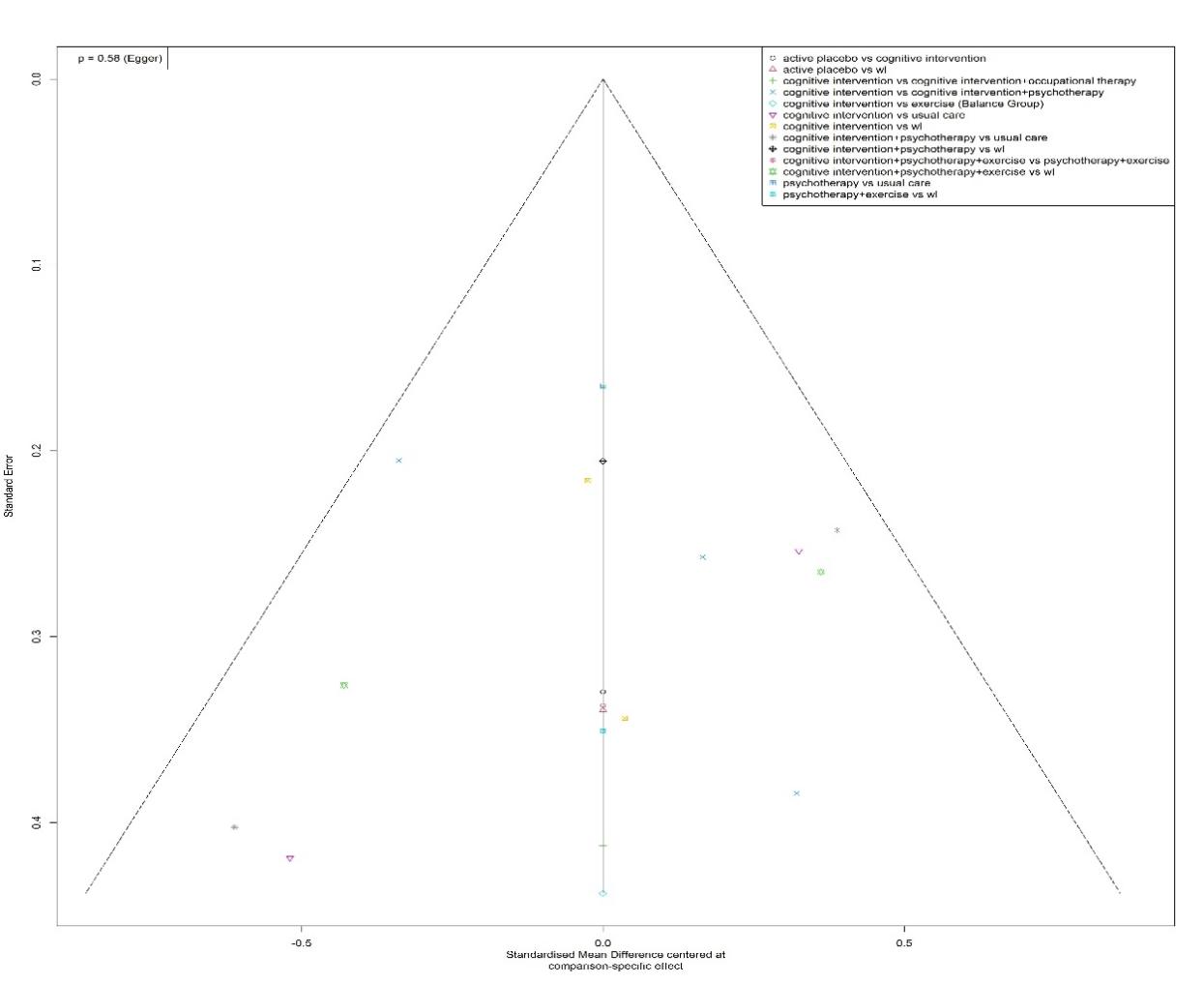


**12.2.8 Depression**


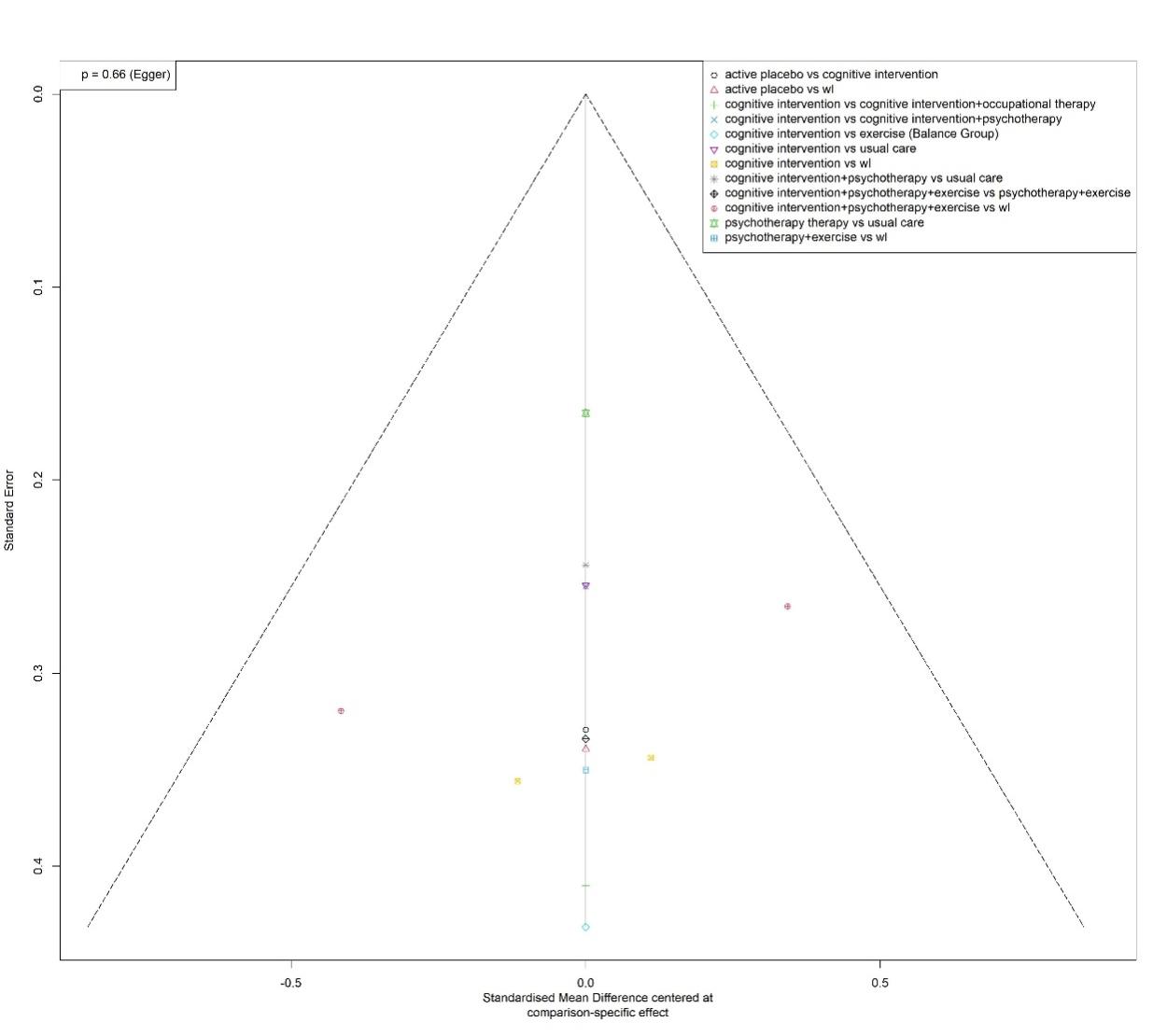


## 12.3 Risk of Bias Chart showing the contribution of low, moderate or high RoB comparisons to each network estimates.

**12.3.1 Subjective memory complaints**


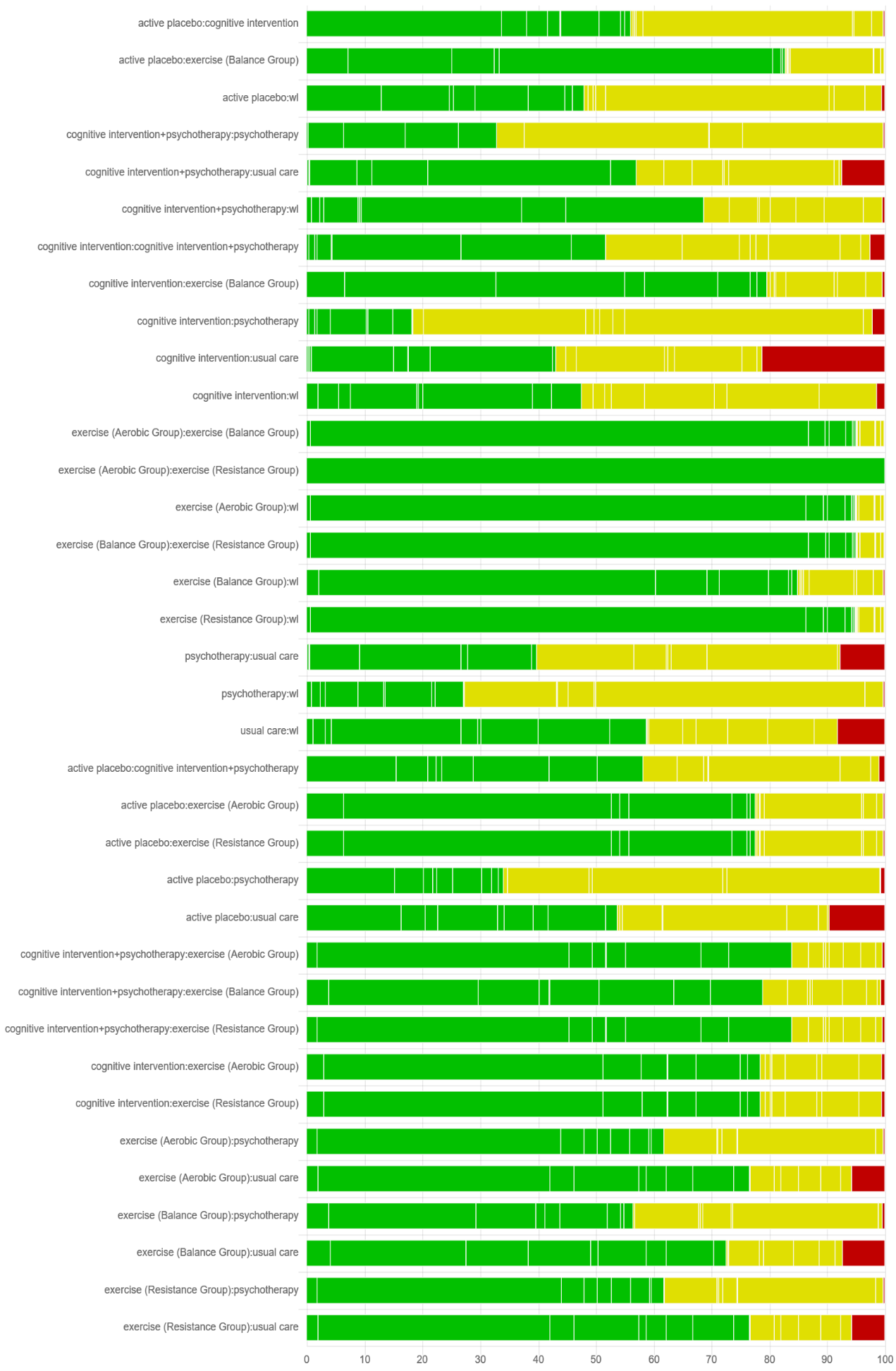


**12.3.2 Global cognitive function**


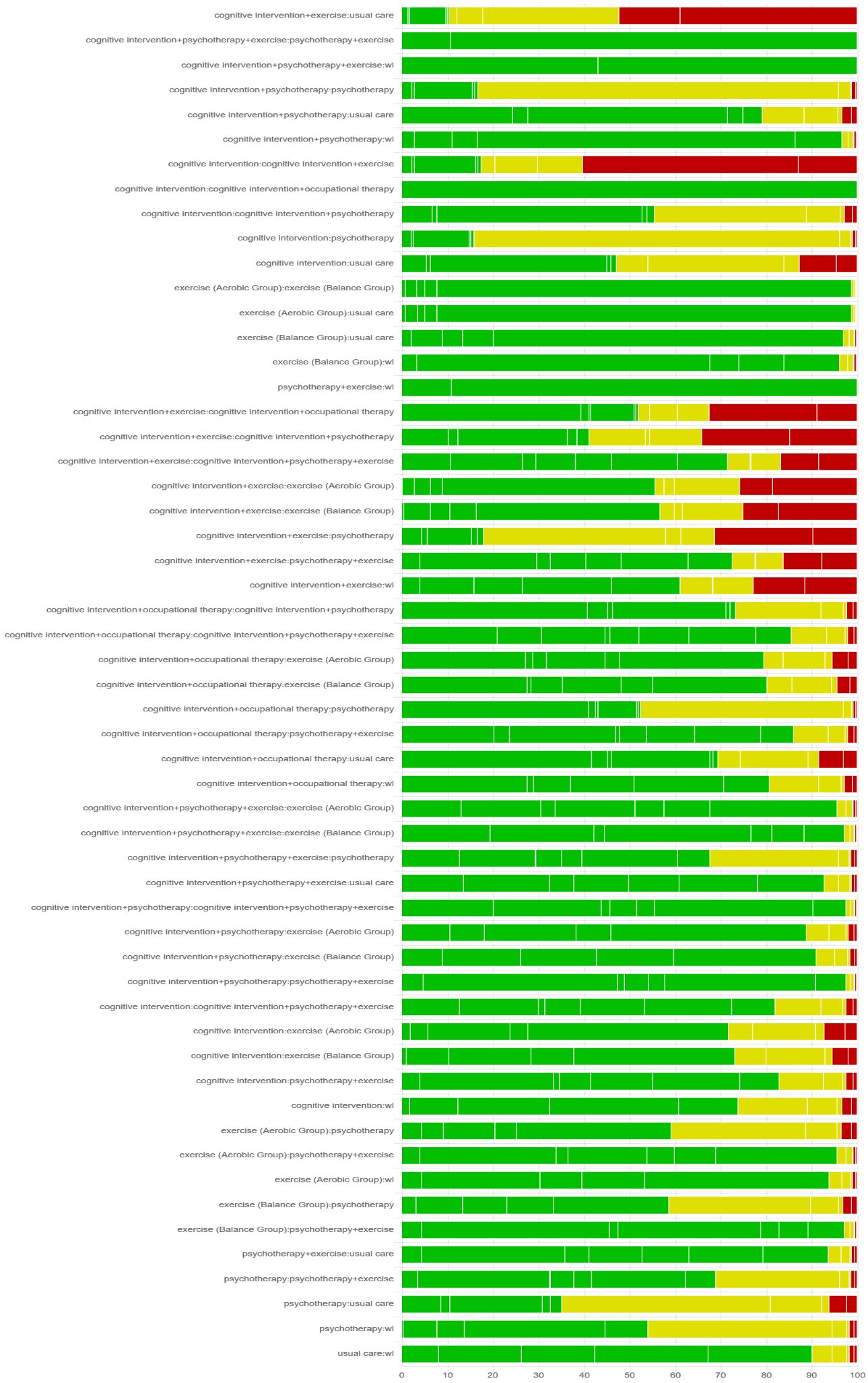


**12.3.3 Language function**


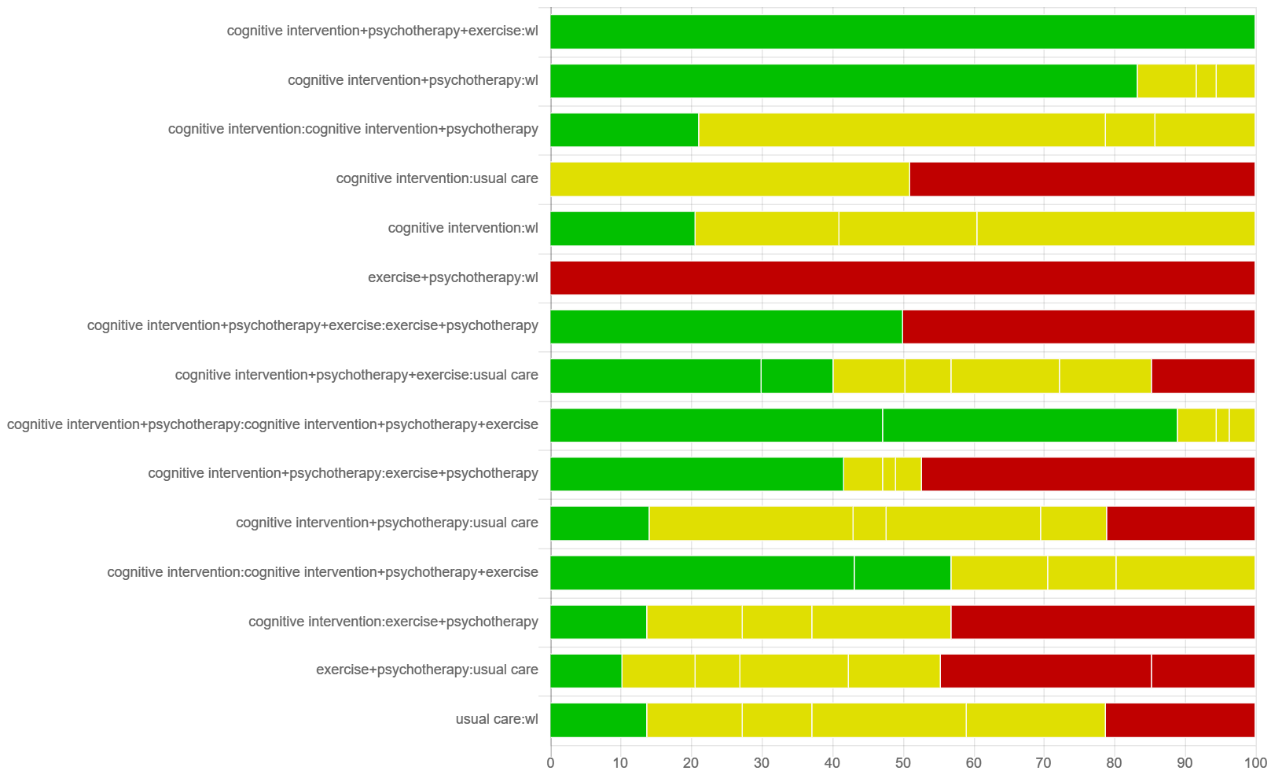


**12.3.4 Visuospatial ability**


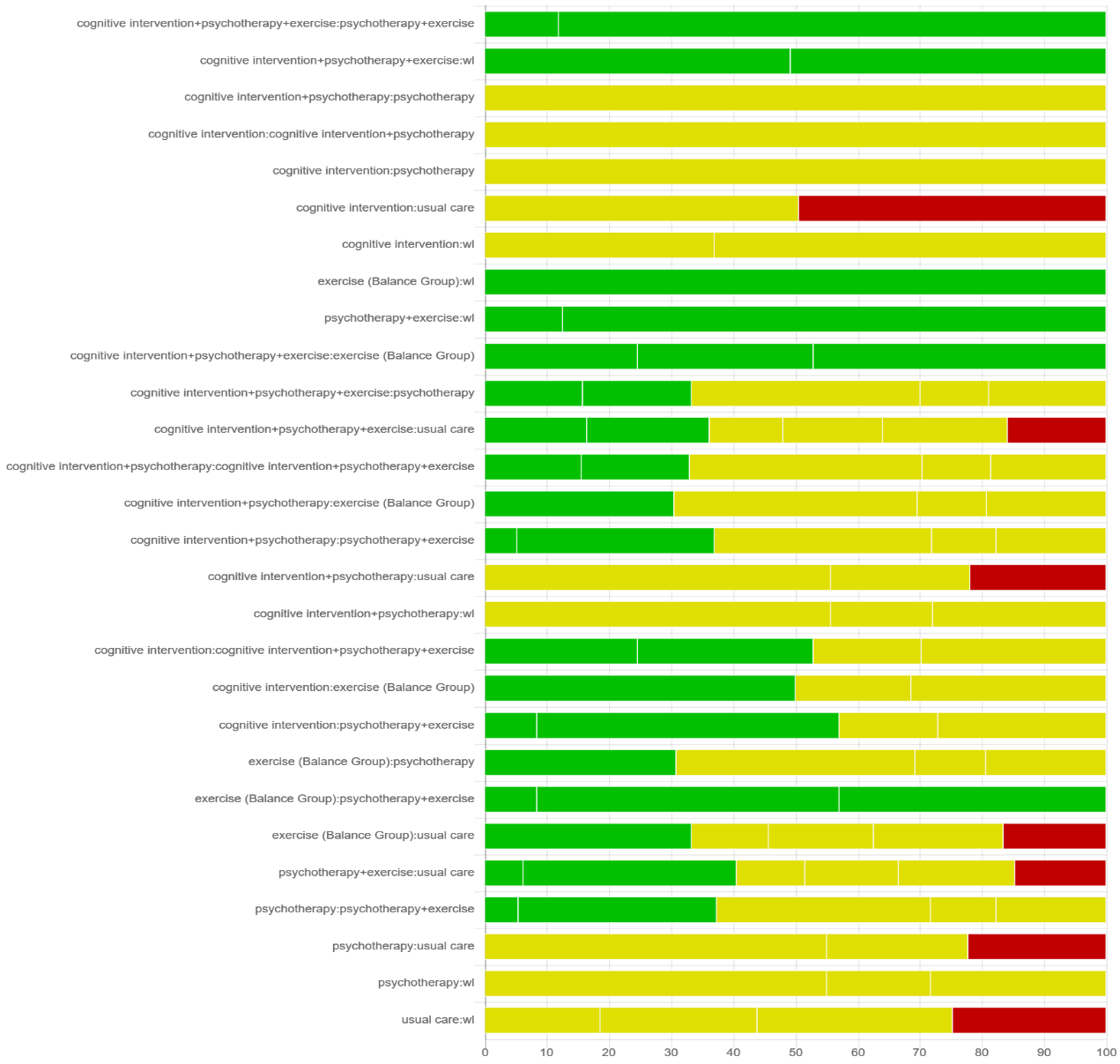


**12.3.5 Executive function**


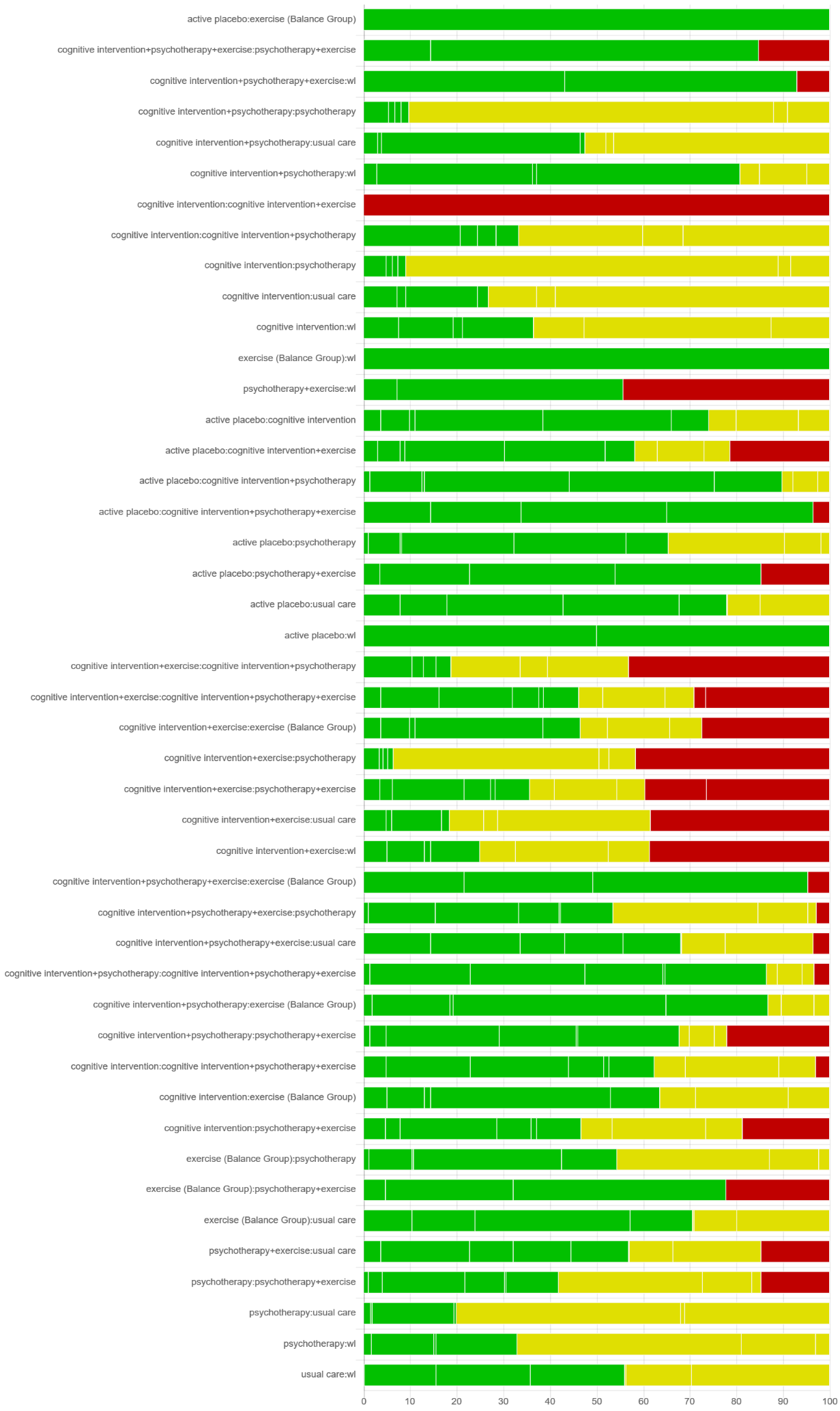


**12.3.6 Attention**


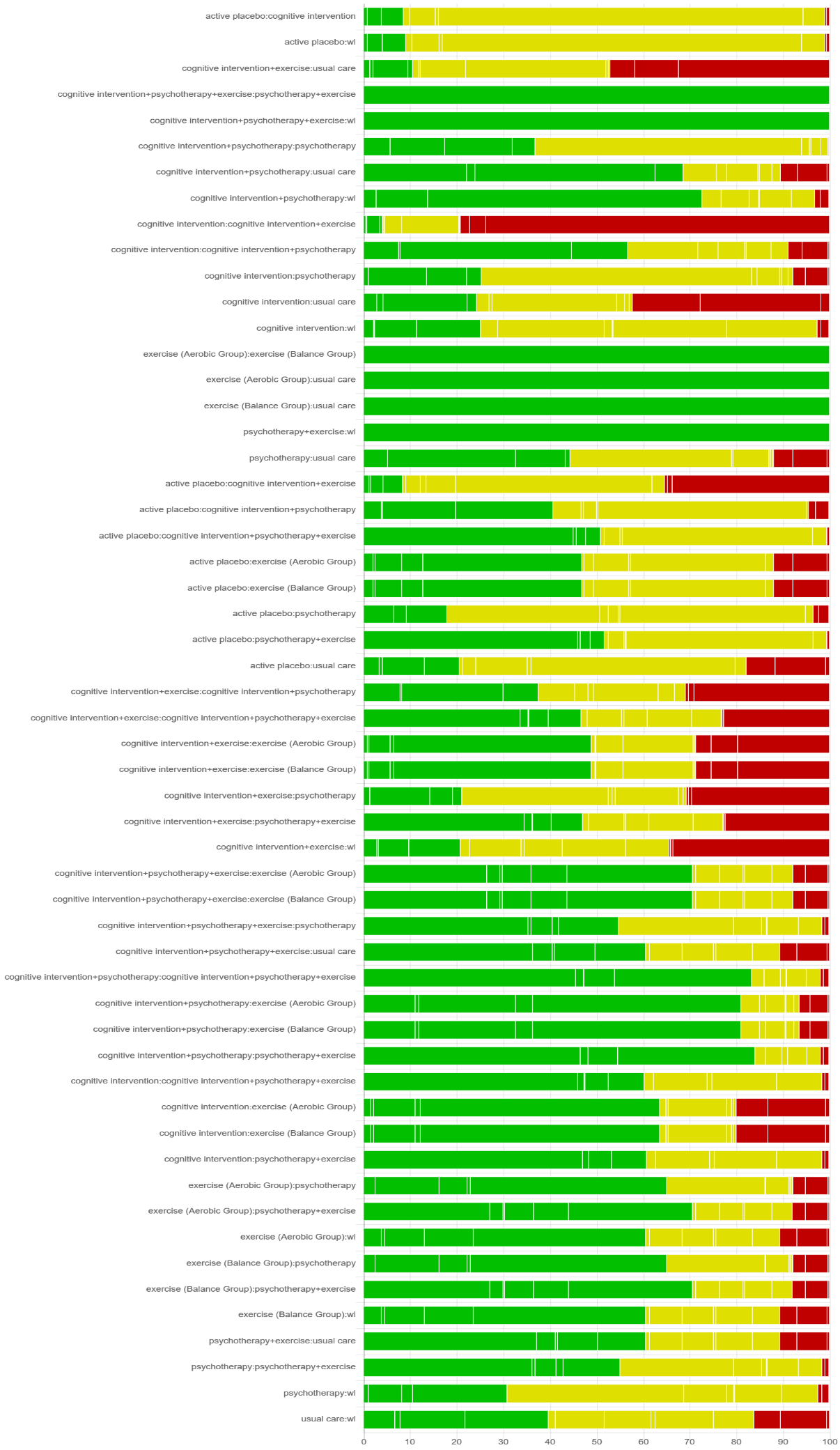


**12.3.7 Anxiety**


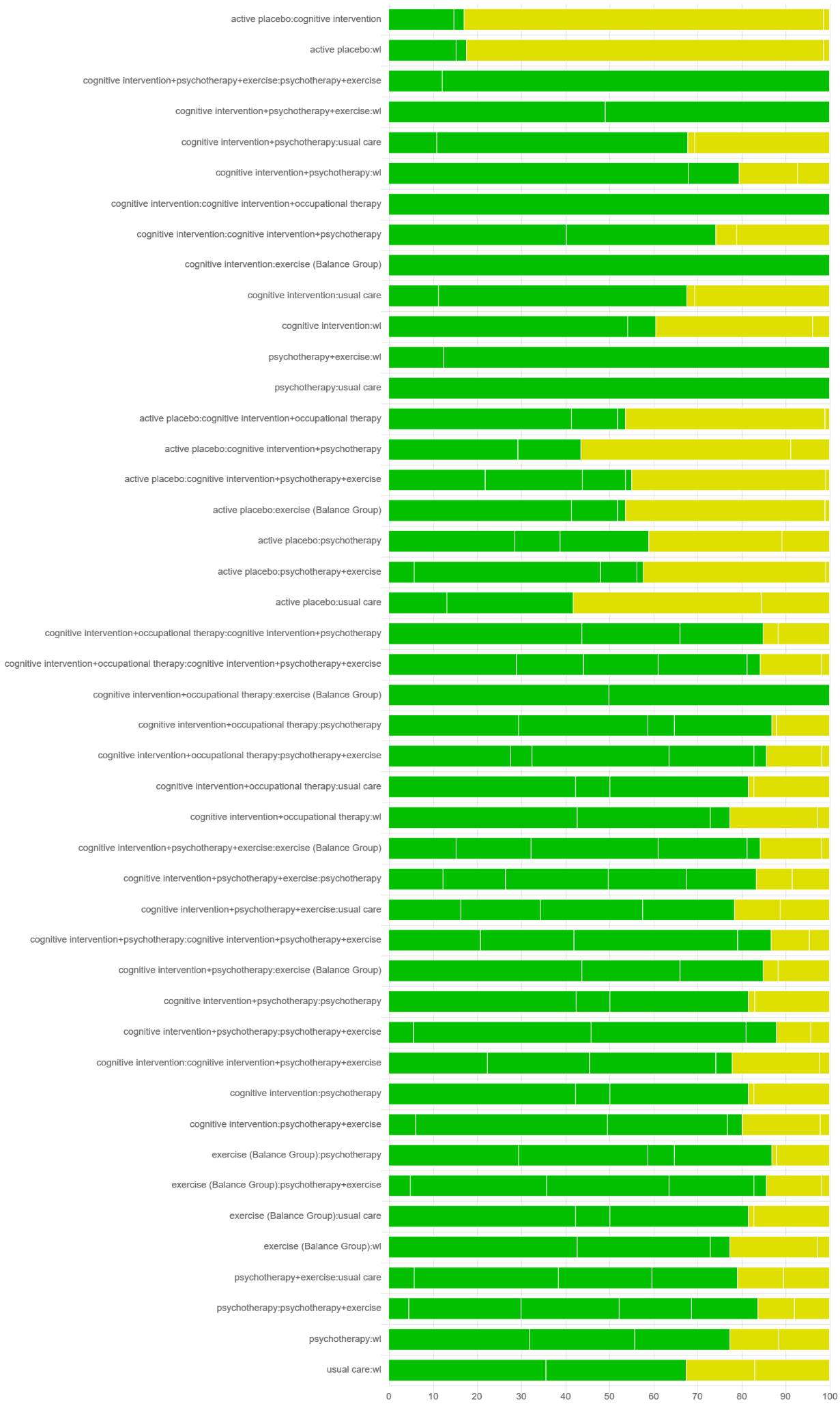


**12.3.8 Depression**


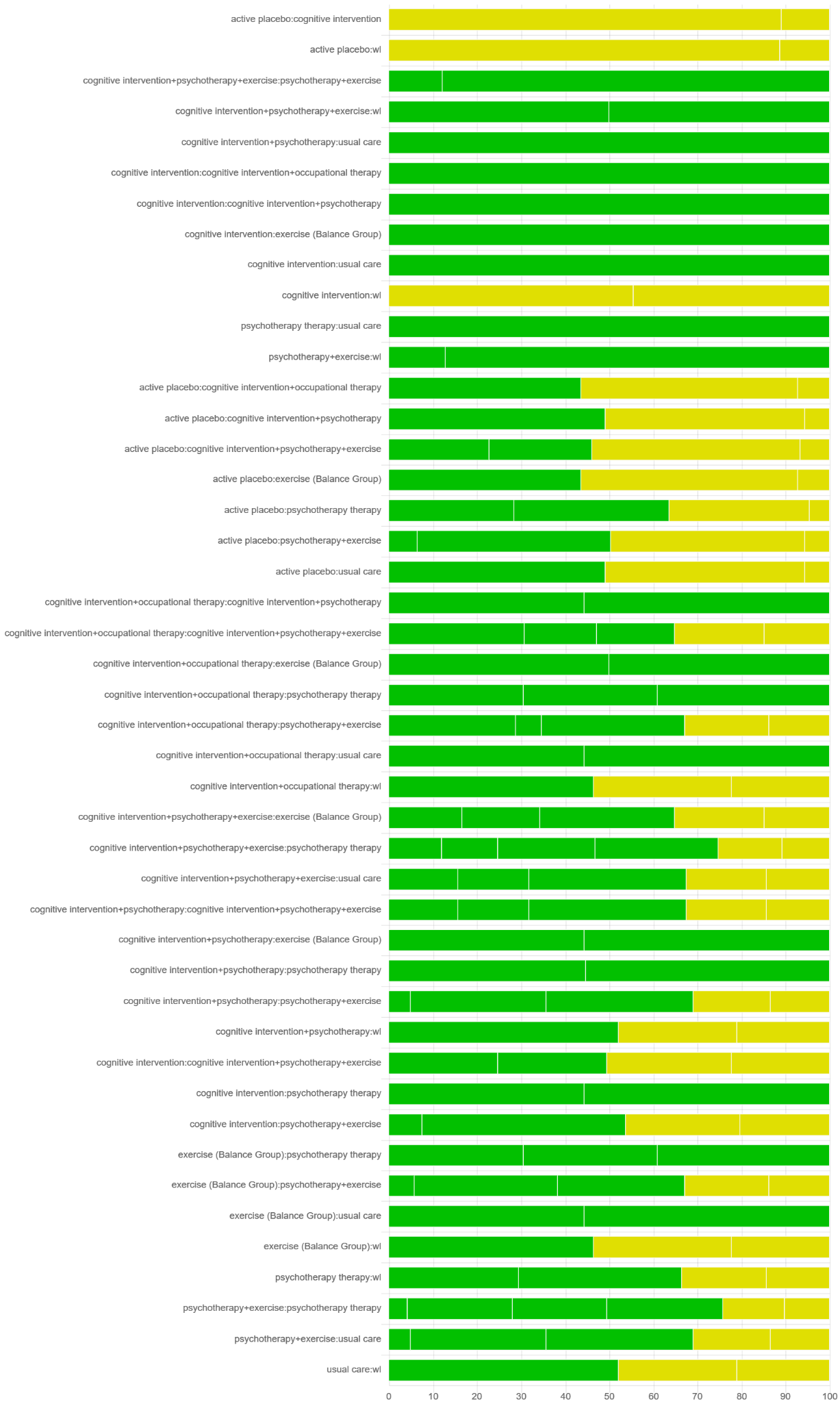


## 12.4 Results of the CINeMA quality assessment

**12.4.1 Subjective memory complaints**

| **Comparison** | **N. of studies** | **Within-study bias** | **Reporting bias** | **Indirectness** | **Imprecision** | **Heterogeneity** | **Incoherence** | **Confidence**  **rating** |
| --- | --- | --- | --- | --- | --- | --- | --- | --- |
| active placebo:cognitive intervention | 2 | No concerns | Low risk | No concerns | Some concerns | Some concerns | No concerns | Low |
| active placebo:exercise (Balance Group) | 1 | No concerns | Low risk | No concerns | Some concerns | Some concerns | No concerns | Low |
| active placebo:wl | 1 | Some concerns | Low risk | No concerns | Major concerns | No concerns | No concerns | Very Low |
| cognitive intervention:cognitive intervention+psychotherapy | 5 | Some concerns | Some concerns | No concerns | No concerns | Major concerns | No concerns | Very Low |
| cognitive intervention:exercise (Balance Group) | 1 | No concerns | Low risk | No concerns | Major concerns | No concerns | Major concerns | Very Low |
| cognitive intervention:psychotherapy | 2 | Some concerns | Low risk | No concerns | Some concerns | Some concerns | No concerns | Very Low |
| cognitive intervention:usual care | 5 | Some concerns | Low risk | No concerns | No concerns | Major concerns | No concerns | Very Low |
| cognitive intervention:wl | 6 | Some concerns | Low risk | No concerns | No concerns | Major concerns | No concerns | Very Low |
| cognitive intervention+psychotherapy:psychotherapy | 1 | Some concerns | Low risk | No concerns | Some concerns | Some concerns | No concerns | Very Low |
| cognitive intervention+psychotherapy:usual care | 2 | Some concerns | Low risk | No concerns | No concerns | Major concerns | No concerns | Very Low |
| cognitive intervention+psychotherapy:wl | 2 | No concerns | Low risk | No concerns | Some concerns | Some concerns | No concerns | Low |
| exercise (Aerobic Group):exercise (Balance Group) | 1 | No concerns | Low risk | No concerns | Major concerns | No concerns | No concerns | Low |
| exercise (Aerobic Group):exercise (Resistance Group) | 1 | No concerns | Low risk | No concerns | Major concerns | No concerns | No concerns | Low |
| exercise (Aerobic Group):wl | 1 | No concerns | Low risk | No concerns | Some concerns | Some concerns | No concerns | Low |
| exercise (Balance Group):exercise (Resistance Group) | 1 | No concerns | Low risk | No concerns | Major concerns | No concerns | No concerns | Low |
| exercise (Balance Group):wl | 1 | No concerns | Low risk | No concerns | Some concerns | Some concerns | No concerns | Low |
| exercise (Resistance Group):wl | 1 | No concerns | Low risk | No concerns | Some concerns | Some concerns | No concerns | Low |
| psychotherapy:usual care | 1 | Some concerns | Low risk | No concerns | Some concerns | Some concerns | No concerns | Very Low |
| psychotherapy:wl | 1 | Some concerns | Low risk | No concerns | Some concerns | No concerns | No concerns | Low |
| usual care:wl | 1 | No concerns | Low risk | No concerns | Some concerns | Some concerns | No concerns | Low |
| active placebo:cognitive intervention+psychotherapy | 0 | No concerns | Low risk | No concerns | Some concerns | Some concerns | No concerns | Low |
| active placebo:exercise (Aerobic Group) | 0 | No concerns | Low risk | No concerns | Some concerns | Some concerns | No concerns | Low |
| active placebo:exercise (Resistance Group) | 0 | No concerns | Low risk | No concerns | Some concerns | Some concerns | No concerns | Low |
| active placebo:psychotherapy | 0 | Some concerns | Low risk | No concerns | Some concerns | Some concerns | No concerns | Very Low |
| active placebo:usual care | 0 | Some concerns | Low risk | No concerns | Some concerns | Some concerns | No concerns | Very Low |
| cognitive intervention:exercise (Aerobic Group) | 0 | No concerns | Low risk | No concerns | Major concerns | No concerns | No concerns | Low |
| cognitive intervention:exercise (Resistance Group) | 0 | No concerns | Low risk | No concerns | Major concerns | No concerns | No concerns | Low |
| cognitive intervention+psychotherapy:exercise (Aerobic Group) | 0 | No concerns | Low risk | No concerns | Major concerns | No concerns | No concerns | Low |
| cognitive intervention+psychotherapy:exercise (Balance Group) | 0 | No concerns | Low risk | No concerns | Major concerns | No concerns | No concerns | Low |
| cognitive intervention+psychotherapy:exercise (Resistance Group) | 0 | No concerns | Low risk | No concerns | Major concerns | No concerns | No concerns | Low |
| exercise (Aerobic Group):psychotherapy | 0 | No concerns | Low risk | No concerns | Major concerns | No concerns | No concerns | Low |
| exercise (Aerobic Group):usual care | 0 | No concerns | Low risk | No concerns | Major concerns | No concerns | No concerns | Low |
| exercise (Balance Group):psychotherapy | 0 | No concerns | Low risk | No concerns | Major concerns | No concerns | No concerns | Low |
| exercise (Balance Group):usual care | 0 | No concerns | Low risk | No concerns | Major concerns | No concerns | No concerns | Low |
| exercise (Resistance Group):psychotherapy | 0 | No concerns | Low risk | No concerns | Major concerns | No concerns | No concerns | Low |
| exercise (Resistance Group):usual care | 0 | No concerns | Low risk | No concerns | Major concerns | No concerns | No concerns | Low |

**12.4.2 General cognitive functioning**

| **Comparison** | **N. of studies** | **Within-study bias** | **Reporting bias** | **Indirectness** | **Imprecision** | **Heterogeneity** | **Incoherence** | **Confidence**  **rating** |
| --- | --- | --- | --- | --- | --- | --- | --- | --- |
| cognitive intervention:cognitive intervention+exercise | 1 | Some concerns | Low risk | No concerns | Major concerns | No concerns | No concerns | Very Low |
| cognitive intervention:cognitive intervention+occupational therapy | 1 | No concerns | Low risk | No concerns | Major concerns | No concerns | No concerns | Low |
| cognitive intervention:cognitive intervention+psychotherapy | 2 | No concerns | Low risk | No concerns | Major concerns | No concerns | No concerns | Low |
| cognitive intervention:psychotherapy | 1 | Some concerns | Low risk | No concerns | Major concerns | No concerns | No concerns | Very Low |
| cognitive intervention:usual care | 2 | Some concerns | Low risk | No concerns | Some concerns | Some concerns | No concerns | Very Low |
| cognitive intervention+exercise:usual care | 2 | Some concerns | Low risk | No concerns | Some concerns | Some concerns | No concerns | Very Low |
| cognitive intervention+psychotherapy:psychotherapy | 1 | Some concerns | Low risk | No concerns | Major concerns | No concerns | No concerns | Very Low |
| cognitive intervention+psychotherapy:usual care | 2 | No concerns | Low risk | No concerns | Some concerns | Some concerns | No concerns | Low |
| cognitive intervention+psychotherapy:wl | 1 | No concerns | Low risk | No concerns | Major concerns | No concerns | No concerns | Low |
| cognitive intervention+psychotherapy+exercise:psychotherapy+exercise | 1 | No concerns | Low risk | No concerns | Major concerns | No concerns | No concerns | Low |
| cognitive intervention+psychotherapy+exercise:wl | 2 | No concerns | Low risk | No concerns | Major concerns | No concerns | No concerns | Low |
| exercise (Aerobic Group):exercise (Balance Group) | 1 | No concerns | Low risk | No concerns | Major concerns | No concerns | No concerns | Low |
| exercise (Aerobic Group):usual care | 1 | No concerns | Low risk | No concerns | Major concerns | No concerns | No concerns | Low |
| exercise (Balance Group):usual care | 1 | No concerns | Low risk | No concerns | Major concerns | No concerns | No concerns | Low |
| exercise (Balance Group):wl | 1 | No concerns | Low risk | No concerns | Major concerns | No concerns | No concerns | Low |
| psychotherapy+exercise:wl | 1 | No concerns | Some concerns | No concerns | Major concerns | No concerns | No concerns | Low |
| cognitive intervention:cognitive intervention+psychotherapy+exercise | 0 | No concerns | Low risk | No concerns | Major concerns | No concerns | No concerns | Low |
| cognitive intervention:exercise (Aerobic Group) | 0 | No concerns | Low risk | No concerns | Major concerns | No concerns | No concerns | Low |
| cognitive intervention:exercise (Balance Group) | 0 | No concerns | Low risk | No concerns | Some concerns | Some concerns | No concerns | Low |
| cognitive intervention:psychotherapy+exercise | 0 | No concerns | Low risk | No concerns | Major concerns | No concerns | No concerns | Low |
| cognitive intervention:wl | 0 | No concerns | Low risk | No concerns | Major concerns | No concerns | No concerns | Low |
| cognitive intervention+exercise:cognitive intervention+occupational therapy | 0 | Some concerns | Low risk | No concerns | Major concerns | No concerns | No concerns | Very Low |
| cognitive intervention+exercise:cognitive intervention+psychotherapy | 0 | Some concerns | Low risk | No concerns | Major concerns | No concerns | No concerns | Very Low |
| cognitive intervention+exercise:cognitive intervention+psychotherapy+exercise | 0 | No concerns | Low risk | No concerns | Major concerns | No concerns | No concerns | Low |
| cognitive intervention+exercise:exercise (Aerobic Group) | 0 | Some concerns | Low risk | No concerns | Major concerns | No concerns | No concerns | Very Low |
| cognitive intervention+exercise:exercise (Balance Group) | 0 | Some concerns | Low risk | No concerns | Major concerns | No concerns | No concerns | Very Low |
| cognitive intervention+exercise:psychotherapy | 0 | Some concerns | Low risk | No concerns | Major concerns | No concerns | No concerns | Very Low |
| cognitive intervention+exercise:psychotherapy+exercise | 0 | No concerns | Low risk | No concerns | Major concerns | No concerns | No concerns | Low |
| cognitive intervention+exercise:wl | 0 | Some concerns | Low risk | No concerns | Major concerns | No concerns | No concerns | Very Low |
| cognitive intervention+occupational therapy:cognitive intervention+psychotherapy | 0 | No concerns | Low risk | No concerns | Major concerns | No concerns | No concerns | Low |
| cognitive intervention+occupational therapy:cognitive intervention+psychotherapy+exercise | 0 | No concerns | Low risk | No concerns | Major concerns | No concerns | No concerns | Low |
| cognitive intervention+occupational therapy:exercise (Aerobic Group) | 0 | No concerns | Low risk | No concerns | Major concerns | No concerns | No concerns | Low |
| cognitive intervention+occupational therapy:exercise (Balance Group) | 0 | No concerns | Low risk | No concerns | Major concerns | No concerns | No concerns | Low |
| cognitive intervention+occupational therapy:psychotherapy | 0 | No concerns | Low risk | No concerns | Major concerns | No concerns | No concerns | Low |
| cognitive intervention+occupational therapy:psychotherapy+exercise | 0 | No concerns | Low risk | No concerns | Major concerns | No concerns | No concerns | Low |
| cognitive intervention+occupational therapy:usual care | 0 | No concerns | Low risk | No concerns | Major concerns | No concerns | No concerns | Low |
| cognitive intervention+occupational therapy:wl | 0 | No concerns | Low risk | No concerns | Major concerns | No concerns | No concerns | Low |
| cognitive intervention+psychotherapy:cognitive intervention+psychotherapy+exercise | 0 | No concerns | Low risk | No concerns | Some concerns | Some concerns | No concerns | Low |
| cognitive intervention+psychotherapy:exercise (Aerobic Group) | 0 | No concerns | Low risk | No concerns | Major concerns | No concerns | No concerns | Low |
| cognitive intervention+psychotherapy:exercise (Balance Group) | 0 | No concerns | Low risk | No concerns | Some concerns | Some concerns | No concerns | Low |
| cognitive intervention+psychotherapy:psychotherapy+exercise | 0 | No concerns | Low risk | No concerns | Major concerns | No concerns | No concerns | Low |
| cognitive intervention+psychotherapy+exercise:exercise (Aerobic Group) | 0 | No concerns | Low risk | No concerns | Major concerns | No concerns | No concerns | Low |
| cognitive intervention+psychotherapy+exercise:exercise (Balance Group) | 0 | No concerns | Low risk | No concerns | Major concerns | No concerns | No concerns | Low |
| cognitive intervention+psychotherapy+exercise:psychotherapy | 0 | No concerns | Low risk | No concerns | Major concerns | No concerns | No concerns | Low |
| cognitive intervention+psychotherapy+exercise:usual care | 0 | No concerns | Low risk | No concerns | Major concerns | No concerns | No concerns | Low |
| exercise (Aerobic Group):psychotherapy | 0 | No concerns | Low risk | No concerns | Major concerns | No concerns | No concerns | Low |
| exercise (Aerobic Group):psychotherapy+exercise | 0 | No concerns | Low risk | No concerns | Major concerns | No concerns | No concerns | Low |
| exercise (Aerobic Group):wl | 0 | No concerns | Low risk | No concerns | Major concerns | No concerns | No concerns | Low |
| exercise (Balance Group):psychotherapy | 0 | No concerns | Low risk | No concerns | Major concerns | No concerns | No concerns | Low |
| exercise (Balance Group):psychotherapy+exercise | 0 | No concerns | Low risk | No concerns | Major concerns | No concerns | No concerns | Low |
| psychotherapy:psychotherapy+exercise | 0 | No concerns | Low risk | No concerns | Major concerns | No concerns | No concerns | Low |
| psychotherapy:usual care | 0 | Some concerns | Low risk | No concerns | Major concerns | No concerns | No concerns | Very Low |
| psychotherapy:wl | 0 | No concerns | Low risk | No concerns | Major concerns | No concerns | No concerns | Low |
| psychotherapy+exercise:usual care | 0 | No concerns | Low risk | No concerns | Major concerns | No concerns | No concerns | Low |
| usual care:wl | 0 | No concerns | Low risk | No concerns | Major concerns | No concerns | No concerns | Low |

**12.4.3 Language function**

| **Comparison** | **N. of studies** | **Within-study bias** | **Reporting bias** | **Indirectness** | **Imprecision** | **Heterogeneity** | **Incoherence** | **Confidence**  **rating** |
| --- | --- | --- | --- | --- | --- | --- | --- | --- |
| cognitive intervention:cognitive intervention+psychotherapy | 2 | No concerns | Low risk | No concerns | Major concerns | No concerns | No concerns | Low |
| cognitive intervention:usual care | 2 | Some concerns | Low risk | No concerns | Some concerns | Some concerns | No concerns | Very Low |
| cognitive intervention+psychotherapy:usual care | 2 | No concerns | Low risk | No concerns | Some concerns | Some concerns | No concerns | Low |
| cognitive intervention+psychotherapy:wl | 1 | No concerns | Low risk | No concerns | Major concerns | No concerns | No concerns | Low |
| cognitive intervention+psychotherapy+exercise:psychotherapy+exercise | 1 | No concerns | Low risk | No concerns | Major concerns | No concerns | No concerns | Low |
| cognitive intervention+psychotherapy+exercise:wl | 2 | No concerns | Low risk | No concerns | Major concerns | No concerns | No concerns | Low |
| psychotherapy+exercise:wl | 1 | No concerns | Some concerns | No concerns | Major concerns | No concerns | No concerns | Very Low |
| cognitive intervention:cognitive intervention+psychotherapy+exercise | 0 | No concerns | Low risk | No concerns | Major concerns | No concerns | No concerns | Low |
| cognitive intervention:psychotherapy+exercise | 0 | No concerns | Low risk | No concerns | Major concerns | No concerns | No concerns | Low |
| cognitive intervention:wl | 0 | No concerns | Low risk | No concerns | Major concerns | No concerns | No concerns | Low |
| cognitive intervention+psychotherapy:cognitive intervention+psychotherapy+exercise | 0 | No concerns | Low risk | No concerns | Some concerns | Some concerns | No concerns | Low |
| cognitive intervention+psychotherapy:psychotherapy+exercise | 0 | No concerns | Low risk | No concerns | Major concerns | No concerns | No concerns | Low |
| cognitive intervention+psychotherapy+exercise:usual care | 0 | No concerns | Low risk | No concerns | Major concerns | No concerns | No concerns | Low |
| psychotherapy+exercise:usual care | 0 | No concerns | Low risk | No concerns | Major concerns | No concerns | No concerns | Low |
| usual care:wl | 0 | No concerns | Low risk | No concerns | Major concerns | No concerns | No concerns | Low |

**12.4.4 Executive function**

| **Comparison** | **N. of studies** | **Within-study bias** | **Reporting bias** | **Indirectness** | **Imprecision** | **Heterogeneity** | **Incoherence** | **Confidence**  **rating** |
| --- | --- | --- | --- | --- | --- | --- | --- | --- |
| active placebo:exercise (Balance Group) | 1 | No concerns | Low risk | No concerns | Some concerns | Some concerns | Some concerns | Very Low |
| cognitive intervention:cognitive intervention+exercise | 1 | Major concerns | Low risk | No concerns | Major concerns | No concerns | Some concerns | Very Low |
| cognitive intervention:cognitive intervention+psychotherapy | 3 | Some concerns | Low risk | No concerns | Some concerns | Some concerns | No concerns | Very Low |
| cognitive intervention:psychotherapy | 1 | Some concerns | Low risk | No concerns | Major concerns | No concerns | No concerns | Very Low |
| cognitive intervention:usual care | 1 | Some concerns | Low risk | No concerns | Major concerns | No concerns | No concerns | Very Low |
| cognitive intervention:wl | 1 | Some concerns | Low risk | No concerns | Major concerns | No concerns | No concerns | Very Low |
| cognitive intervention+psychotherapy:psychotherapy | 1 | Some concerns | Low risk | No concerns | Major concerns | No concerns | No concerns | Very Low |
| cognitive intervention+psychotherapy:usual care | 2 | Some concerns | Low risk | No concerns | Some concerns | Some concerns | No concerns | Very Low |
| cognitive intervention+psychotherapy:wl | 2 | No concerns | Low risk | No concerns | Major concerns | No concerns | No concerns | Low |
| cognitive intervention+psychotherapy+exercise:psychotherapy+exercise | 1 | No concerns | Low risk | No concerns | Major concerns | No concerns | No concerns | Low |
| cognitive intervention+psychotherapy+exercise:wl | 2 | No concerns | Low risk | No concerns | Major concerns | No concerns | No concerns | Low |
| exercise (Balance Group):wl | 1 | No concerns | Low risk | No concerns | Major concerns | No concerns | Some concerns | Very Low |
| psychotherapy+exercise:wl | 2 | Some concerns | Low risk | No concerns | Some concerns | Some concerns | No concerns | Very Low |
| active placebo:cognitive intervention | 0 | No concerns | Low risk | No concerns | Major concerns | No concerns | Some concerns | Very Low |
| active placebo:cognitive intervention+exercise | 0 | Some concerns | Low risk | No concerns | Major concerns | No concerns | Some concerns | Very Low |
| active placebo:cognitive intervention+psychotherapy | 0 | No concerns | Low risk | No concerns | Major concerns | No concerns | Some concerns | Very Low |
| active placebo:cognitive intervention+psychotherapy+exercise | 0 | No concerns | Low risk | No concerns | Major concerns | No concerns | Some concerns | Very Low |
| active placebo:psychotherapy | 0 | No concerns | Low risk | No concerns | Major concerns | No concerns | Some concerns | Very Low |
| active placebo:psychotherapy+exercise | 0 | No concerns | Low risk | No concerns | Major concerns | No concerns | Some concerns | Very Low |
| active placebo:usual care | 0 | No concerns | Low risk | No concerns | Major concerns | No concerns | Some concerns | Very Low |
| active placebo:wl | 0 | No concerns | Low risk | No concerns | Major concerns | No concerns | Some concerns | Very Low |
| cognitive intervention:cognitive intervention+psychotherapy+exercise | 0 | No concerns | Low risk | No concerns | Major concerns | No concerns | Some concerns | Very Low |
| cognitive intervention:exercise (Balance Group) | 0 | No concerns | Low risk | No concerns | Major concerns | No concerns | Some concerns | Very Low |
| cognitive intervention:psychotherapy+exercise | 0 | Some concerns | Low risk | No concerns | Major concerns | No concerns | Some concerns | Very Low |
| cognitive intervention+exercise:cognitive intervention+psychotherapy | 0 | Some concerns | Low risk | No concerns | Major concerns | No concerns | Some concerns | Very Low |
| cognitive intervention+exercise:cognitive intervention+psychotherapy+exercise | 0 | Some concerns | Low risk | No concerns | Major concerns | No concerns | Some concerns | Very Low |
| cognitive intervention+exercise:exercise (Balance Group) | 0 | Some concerns | Low risk | No concerns | Major concerns | No concerns | Some concerns | Very Low |
| cognitive intervention+exercise:psychotherapy | 0 | Some concerns | Low risk | No concerns | Major concerns | No concerns | Some concerns | Very Low |
| cognitive intervention+exercise:psychotherapy+exercise | 0 | Some concerns | Low risk | No concerns | Major concerns | No concerns | Some concerns | Very Low |
| cognitive intervention+exercise:usual care | 0 | Some concerns | Low risk | No concerns | Major concerns | No concerns | Some concerns | Very Low |
| cognitive intervention+exercise:wl | 0 | Some concerns | Low risk | No concerns | Major concerns | No concerns | Some concerns | Very Low |
| cognitive intervention+psychotherapy:cognitive intervention+psychotherapy+exercise | 0 | No concerns | Low risk | No concerns | Major concerns | No concerns | Some concerns | Very Low |
| cognitive intervention+psychotherapy:exercise (Balance Group) | 0 | No concerns | Low risk | No concerns | Major concerns | No concerns | Some concerns | Very Low |
| cognitive intervention+psychotherapy:psychotherapy+exercise | 0 | Some concerns | Low risk | No concerns | Major concerns | No concerns | Some concerns | Very Low |
| cognitive intervention+psychotherapy+exercise:exercise (Balance Group) | 0 | No concerns | Low risk | No concerns | Major concerns | No concerns | Some concerns | Very Low |
| cognitive intervention+psychotherapy+exercise:psychotherapy | 0 | No concerns | Low risk | No concerns | Major concerns | No concerns | Some concerns | Very Low |
| cognitive intervention+psychotherapy+exercise:usual care | 0 | No concerns | Low risk | No concerns | Major concerns | No concerns | Some concerns | Very Low |
| exercise (Balance Group):psychotherapy | 0 | No concerns | Low risk | No concerns | Major concerns | No concerns | Some concerns | Very Low |
| exercise (Balance Group):psychotherapy+exercise | 0 | No concerns | Low risk | No concerns | Major concerns | No concerns | Some concerns | Very Low |
| exercise (Balance Group):usual care | 0 | No concerns | Low risk | No concerns | Major concerns | No concerns | Some concerns | Very Low |
| psychotherapy:psychotherapy+exercise | 0 | Some concerns | Low risk | No concerns | Major concerns | No concerns | Some concerns | Very Low |
| psychotherapy:usual care | 0 | Some concerns | Low risk | No concerns | Major concerns | No concerns | Some concerns | Very Low |
| psychotherapy:wl | 0 | Some concerns | Low risk | No concerns | Major concerns | No concerns | Some concerns | Very Low |
| psychotherapy+exercise:usual care | 0 | Some concerns | Low risk | No concerns | Major concerns | No concerns | Some concerns | Very Low |
| usual care:wl | 0 | No concerns | Low risk | No concerns | Major concerns | No concerns | Some concerns | Very Low |

**12.4.5 Visuospatial ability**

| **Comparison** | **N. of studies** | **Within-study bias** | **Reporting bias** | **Indirectness** | **Imprecision** | **Heterogeneity** | **Incoherence** | **Confidence**  **rating** |
| --- | --- | --- | --- | --- | --- | --- | --- | --- |
| cognitive intervention:cognitive intervention+psychotherapy | 1 | Some concerns | Low risk | No concerns | Major concerns | No concerns | No concerns | Very Low |
| cognitive intervention:psychotherapy | 1 | Some concerns | Low risk | No concerns | Major concerns | No concerns | No concerns | Very Low |
| cognitive intervention:usual care | 2 | Some concerns | Low risk | No concerns | Some concerns | Some concerns | No concerns | Very Low |
| cognitive intervention:wl | 2 | Some concerns | Low risk | No concerns | Some concerns | Some concerns | No concerns | Very Low |
| cognitive intervention+psychotherapy:psychotherapy | 1 | Some concerns | Low risk | No concerns | Major concerns | No concerns | No concerns | Very Low |
| cognitive intervention+psychotherapy+exercise:psychotherapy+exercise | 1 | No concerns | Low risk | No concerns | Some concerns | Some concerns | No concerns | Low |
| cognitive intervention+psychotherapy+exercise:wl | 2 | No concerns | Low risk | No concerns | Some concerns | Some concerns | No concerns | Low |
| exercise (Balance Group):wl | 1 | No concerns | Low risk | No concerns | Major concerns | No concerns | No concerns | Low |
| psychotherapy+exercise:wl | 1 | No concerns | Low risk | No concerns | Some concerns | Some concerns | No concerns | Low |
| cognitive intervention:cognitive intervention+psychotherapy+exercise | 0 | No concerns | Low risk | No concerns | Some concerns | Some concerns | No concerns | Low |
| cognitive intervention:exercise (Balance Group) | 0 | Some concerns | Low risk | No concerns | Some concerns | Some concerns | No concerns | Very Low |
| cognitive intervention:psychotherapy+exercise | 0 | No concerns | Low risk | No concerns | Some concerns | Some concerns | No concerns | Low |
| cognitive intervention+psychotherapy:cognitive intervention+psychotherapy+exercise | 0 | Some concerns | Low risk | No concerns | Major concerns | No concerns | No concerns | Very Low |
| cognitive intervention+psychotherapy:exercise (Balance Group) | 0 | Some concerns | Low risk | No concerns | Some concerns | Some concerns | No concerns | Very Low |
| cognitive intervention+psychotherapy:psychotherapy+exercise | 0 | Some concerns | Low risk | No concerns | Some concerns | Some concerns | No concerns | Very Low |
| cognitive intervention+psychotherapy:usual care | 0 | Some concerns | Low risk | No concerns | Major concerns | No concerns | No concerns | Very Low |
| cognitive intervention+psychotherapy:wl | 0 | Some concerns | Low risk | No concerns | Some concerns | Some concerns | No concerns | Very Low |
| cognitive intervention+psychotherapy+exercise:exercise (Balance Group) | 0 | No concerns | Low risk | No concerns | Major concerns | No concerns | No concerns | Low |
| cognitive intervention+psychotherapy+exercise:psychotherapy | 0 | Some concerns | Low risk | No concerns | Major concerns | No concerns | No concerns | Very Low |
| cognitive intervention+psychotherapy+exercise:usual care | 0 | Some concerns | Low risk | No concerns | Major concerns | No concerns | No concerns | Very Low |
| exercise (Balance Group):psychotherapy | 0 | Some concerns | Low risk | No concerns | Major concerns | No concerns | No concerns | Very Low |
| exercise (Balance Group):psychotherapy+exercise | 0 | No concerns | Low risk | No concerns | Major concerns | No concerns | No concerns | Low |
| exercise (Balance Group):usual care | 0 | Some concerns | Low risk | No concerns | Major concerns | No concerns | No concerns | Very Low |
| psychotherapy:psychotherapy+exercise | 0 | Some concerns | Low risk | No concerns | Some concerns | Some concerns | No concerns | Very Low |
| psychotherapy:usual care | 0 | Some concerns | Low risk | No concerns | Major concerns | No concerns | No concerns | Very Low |
| psychotherapy:wl | 0 | Some concerns | Low risk | No concerns | Major concerns | No concerns | No concerns | Very Low |
| psychotherapy+exercise:usual care | 0 | Some concerns | Low risk | No concerns | Some concerns | Some concerns | No concerns | Very Low |
| usual care:wl | 0 | Some concerns | Low risk | No concerns | Some concerns | Some concerns | No concerns | Very Low |

**12.4.6 Attention**

| **Comparison** | **N. of studies** | **Within-study bias** | **Reporting bias** | **Indirectness** | **Imprecision** | **Heterogeneity** | **Incoherence** | **Confidence**  **rating** |
| --- | --- | --- | --- | --- | --- | --- | --- | --- |
| active placebo:cognitive intervention | 1 | Some concerns | Low risk | No concerns | Major concerns | No concerns | No concerns | Very Low |
| active placebo:wl | 1 | Some concerns | Low risk | No concerns | Major concerns | No concerns | No concerns | Very Low |
| cognitive intervention:cognitive intervention+exercise | 1 | Major concerns | Low risk | No concerns | Some concerns | No concerns | No concerns | Very Low |
| cognitive intervention:cognitive intervention+psychotherapy | 2 | Some concerns | Low risk | No concerns | No concerns | Major concerns | No concerns | Very Low |
| cognitive intervention:psychotherapy | 1 | Some concerns | Low risk | No concerns | Some concerns | Some concerns | No concerns | Very Low |
| cognitive intervention:usual care | 4 | Some concerns | Low risk | No concerns | No concerns | Some concerns | No concerns | Low |
| cognitive intervention:wl | 3 | Some concerns | Low risk | No concerns | No concerns | Some concerns | No concerns | Low |
| cognitive intervention+exercise:usual care | 1 | Some concerns | Low risk | No concerns | Some concerns | No concerns | No concerns | Low |
| cognitive intervention+psychotherapy:psychotherapy | 1 | Some concerns | Low risk | No concerns | Major concerns | No concerns | No concerns | Very Low |
| cognitive intervention+psychotherapy:usual care | 2 | No concerns | Low risk | No concerns | No concerns | Some concerns | No concerns | Moderate |
| cognitive intervention+psychotherapy:wl | 1 | No concerns | Low risk | No concerns | Some concerns | No concerns | No concerns | Moderate |
| cognitive intervention+psychotherapy+exercise:psychotherapy+exercise | 1 | No concerns | Low risk | No concerns | Major concerns | No concerns | No concerns | Low |
| cognitive intervention+psychotherapy+exercise:wl | 1 | No concerns | Low risk | No concerns | Major concerns | No concerns | No concerns | Low |
| exercise (Aerobic Group):exercise (Balance Group) | 1 | No concerns | Low risk | No concerns | Major concerns | No concerns | No concerns | Low |
| exercise (Aerobic Group):usual care | 1 | No concerns | Low risk | No concerns | Some concerns | Some concerns | No concerns | Low |
| exercise (Balance Group):usual care | 1 | No concerns | Low risk | No concerns | Some concerns | Some concerns | No concerns | Low |
| psychotherapy:usual care | 1 | Some concerns | Low risk | No concerns | Some concerns | Some concerns | No concerns | Very Low |
| psychotherapy+exercise:wl | 1 | No concerns | Low risk | No concerns | Major concerns | No concerns | No concerns | Low |
| active placebo:cognitive intervention+exercise | 0 | Some concerns | Low risk | No concerns | Major concerns | No concerns | No concerns | Very Low |
| active placebo:cognitive intervention+psychotherapy | 0 | Some concerns | Low risk | No concerns | Major concerns | No concerns | No concerns | Very Low |
| active placebo:cognitive intervention+psychotherapy+exercise | 0 | No concerns | Low risk | No concerns | Major concerns | No concerns | No concerns | Low |
| active placebo:exercise (Aerobic Group) | 0 | Some concerns | Low risk | No concerns | Major concerns | No concerns | No concerns | Very Low |
| active placebo:exercise (Balance Group) | 0 | Some concerns | Low risk | No concerns | Major concerns | No concerns | No concerns | Very Low |
| active placebo:psychotherapy | 0 | Some concerns | Low risk | No concerns | Major concerns | No concerns | No concerns | Very Low |
| active placebo:psychotherapy+exercise | 0 | No concerns | Low risk | No concerns | Major concerns | No concerns | No concerns | Low |
| active placebo:usual care | 0 | Some concerns | Low risk | No concerns | Major concerns | No concerns | No concerns | Very Low |
| cognitive intervention:cognitive intervention+psychotherapy+exercise | 0 | No concerns | Low risk | No concerns | Major concerns | No concerns | No concerns | Low |
| cognitive intervention:exercise (Aerobic Group) | 0 | Some concerns | Low risk | No concerns | Some concerns | Some concerns | No concerns | Very Low |
| cognitive intervention:exercise (Balance Group) | 0 | Some concerns | Low risk | No concerns | Major concerns | No concerns | No concerns | Very Low |
| cognitive intervention:psychotherapy+exercise | 0 | No concerns | Low risk | No concerns | Major concerns | No concerns | No concerns | Low |
| cognitive intervention+exercise:cognitive intervention+psychotherapy | 0 | Some concerns | Low risk | No concerns | Some concerns | Some concerns | No concerns | Very Low |
| cognitive intervention+exercise:cognitive intervention+psychotherapy+exercise | 0 | Some concerns | Low risk | No concerns | Major concerns | No concerns | No concerns | Very Low |
| cognitive intervention+exercise:exercise (Aerobic Group) | 0 | Some concerns | Low risk | No concerns | Major concerns | No concerns | No concerns | Very Low |
| cognitive intervention+exercise:exercise (Balance Group) | 0 | Some concerns | Low risk | No concerns | Major concerns | No concerns | No concerns | Very Low |
| cognitive intervention+exercise:psychotherapy | 0 | Some concerns | Low risk | No concerns | Major concerns | No concerns | No concerns | Very Low |
| cognitive intervention+exercise:psychotherapy+exercise | 0 | Some concerns | Low risk | No concerns | Major concerns | No concerns | No concerns | Very Low |
| cognitive intervention+exercise:wl | 0 | Some concerns | Low risk | No concerns | Some concerns | No concerns | No concerns | Low |
| cognitive intervention+psychotherapy:cognitive intervention+psychotherapy+exercise | 0 | No concerns | Low risk | No concerns | Major concerns | No concerns | No concerns | Low |
| cognitive intervention+psychotherapy:exercise (Aerobic Group) | 0 | No concerns | Low risk | No concerns | Major concerns | No concerns | No concerns | Low |
| cognitive intervention+psychotherapy:exercise (Balance Group) | 0 | No concerns | Low risk | No concerns | Major concerns | No concerns | No concerns | Low |
| cognitive intervention+psychotherapy:psychotherapy+exercise | 0 | No concerns | Low risk | No concerns | Major concerns | No concerns | No concerns | Low |
| cognitive intervention+psychotherapy+exercise:exercise (Aerobic Group) | 0 | No concerns | Low risk | No concerns | Major concerns | No concerns | No concerns | Low |
| cognitive intervention+psychotherapy+exercise:exercise (Balance Group) | 0 | No concerns | Low risk | No concerns | Major concerns | No concerns | No concerns | Low |
| cognitive intervention+psychotherapy+exercise:psychotherapy | 0 | No concerns | Low risk | No concerns | Major concerns | No concerns | No concerns | Low |
| cognitive intervention+psychotherapy+exercise:usual care | 0 | Some concerns | Low risk | No concerns | Major concerns | No concerns | No concerns | Very Low |
| exercise (Aerobic Group):psychotherapy | 0 | No concerns | Low risk | No concerns | Major concerns | No concerns | No concerns | Low |
| exercise (Aerobic Group):psychotherapy+exercise | 0 | No concerns | Low risk | No concerns | Major concerns | No concerns | No concerns | Low |
| exercise (Aerobic Group):wl | 0 | Some concerns | Low risk | No concerns | Some concerns | Some concerns | No concerns | Very Low |
| exercise (Balance Group):psychotherapy | 0 | No concerns | Low risk | No concerns | Major concerns | No concerns | No concerns | Low |
| exercise (Balance Group):psychotherapy+exercise | 0 | No concerns | Low risk | No concerns | Major concerns | No concerns | No concerns | Low |
| exercise (Balance Group):wl | 0 | Some concerns | Low risk | No concerns | Some concerns | Some concerns | No concerns | Very Low |
| psychotherapy:psychotherapy+exercise | 0 | No concerns | Low risk | No concerns | Major concerns | No concerns | No concerns | Low |
| psychotherapy:wl | 0 | Some concerns | Low risk | No concerns | Some concerns | Some concerns | No concerns | Very Low |
| psychotherapy+exercise:usual care | 0 | Some concerns | Low risk | No concerns | Major concerns | No concerns | No concerns | Very Low |
| usual care:wl | 0 | Some concerns | Low risk | No concerns | No concerns | Major concerns | No concerns | Very Low |

**12.4.7 Anxiety**

| **Comparison** | **N. of studies** | **Within-study bias** | **Reporting bias** | **Indirectness** | **Imprecision** | **Heterogeneity** | **Incoherence** | **Confidence**  **rating** |
| --- | --- | --- | --- | --- | --- | --- | --- | --- |
| active placebo:cognitive intervention | 1 | Some concerns | Low risk | No concerns | Major concerns | No concerns | No concerns | Very Low |
| active placebo:wl | 1 | Some concerns | Low risk | No concerns | Major concerns | No concerns | No concerns | Very Low |
| cognitive intervention:cognitive intervention+occupational therapy | 1 | No concerns | Low risk | No concerns | Major concerns | No concerns | No concerns | Low |
| cognitive intervention:cognitive intervention+psychotherapy | 3 | No concerns | Low risk | No concerns | Some concerns | Some concerns | No concerns | Low |
| cognitive intervention:exercise (Balance Group) | 1 | No concerns | Low risk | No concerns | Some concerns | Some concerns | No concerns | Low |
| cognitive intervention:usual care | 2 | No concerns | Low risk | No concerns | Major concerns | No concerns | No concerns | Low |
| cognitive intervention:wl | 2 | No concerns | Low risk | No concerns | Some concerns | Some concerns | No concerns | Low |
| cognitive intervention+psychotherapy:usual care | 2 | No concerns | Low risk | No concerns | Some concerns | Some concerns | No concerns | Low |
| cognitive intervention+psychotherapy:wl | 1 | No concerns | Low risk | No concerns | Some concerns | Some concerns | No concerns | Low |
| cognitive intervention+psychotherapy+exercise:psychotherapy+exercise | 1 | No concerns | Low risk | No concerns | Some concerns | Some concerns | No concerns | Low |
| cognitive intervention+psychotherapy+exercise:wl | 2 | No concerns | Low risk | No concerns | Some concerns | Some concerns | No concerns | Low |
| psychotherapy:usual care | 1 | No concerns | Low risk | No concerns | Major concerns | No concerns | No concerns | Low |
| psychotherapy+exercise:wl | 1 | No concerns | Low risk | No concerns | Major concerns | No concerns | No concerns | Low |
| active placebo:cognitive intervention+occupational therapy | 0 | No concerns | Low risk | No concerns | Major concerns | No concerns | No concerns | Low |
| active placebo:cognitive intervention+psychotherapy | 0 | Some concerns | Low risk | No concerns | Some concerns | Some concerns | No concerns | Very Low |
| active placebo:cognitive intervention+psychotherapy+exercise | 0 | No concerns | Low risk | No concerns | Some concerns | Some concerns | No concerns | Low |
| active placebo:exercise (Balance Group) | 0 | No concerns | Low risk | No concerns | Major concerns | No concerns | No concerns | Low |
| active placebo:psychotherapy | 0 | No concerns | Low risk | No concerns | Major concerns | No concerns | No concerns | Low |
| active placebo:psychotherapy+exercise | 0 | No concerns | Low risk | No concerns | Major concerns | No concerns | No concerns | Low |
| active placebo:usual care | 0 | Some concerns | Low risk | No concerns | Major concerns | No concerns | No concerns | Very Low |
| cognitive intervention:cognitive intervention+psychotherapy+exercise | 0 | No concerns | Low risk | No concerns | Some concerns | Some concerns | No concerns | Low |
| cognitive intervention:psychotherapy | 0 | No concerns | Low risk | No concerns | Major concerns | No concerns | No concerns | Low |
| cognitive intervention:psychotherapy+exercise | 0 | No concerns | Low risk | No concerns | Major concerns | No concerns | No concerns | Low |
| cognitive intervention+occupational therapy:cognitive intervention+psychotherapy | 0 | No concerns | Low risk | No concerns | Major concerns | No concerns | No concerns | Low |
| cognitive intervention+occupational therapy:cognitive intervention+psychotherapy+exercise | 0 | No concerns | Low risk | No concerns | Major concerns | No concerns | No concerns | Low |
| cognitive intervention+occupational therapy:exercise (Balance Group) | 0 | No concerns | Low risk | No concerns | Major concerns | No concerns | No concerns | Low |
| cognitive intervention+occupational therapy:psychotherapy | 0 | No concerns | Low risk | No concerns | Major concerns | No concerns | No concerns | Low |
| cognitive intervention+occupational therapy:psychotherapy+exercise | 0 | No concerns | Low risk | No concerns | Major concerns | No concerns | No concerns | Low |
| cognitive intervention+occupational therapy:usual care | 0 | No concerns | Low risk | No concerns | Major concerns | No concerns | No concerns | Low |
| cognitive intervention+occupational therapy:wl | 0 | No concerns | Low risk | No concerns | Major concerns | No concerns | No concerns | Low |
| cognitive intervention+psychotherapy:cognitive intervention+psychotherapy+exercise | 0 | No concerns | Low risk | No concerns | Major concerns | No concerns | No concerns | Low |
| cognitive intervention+psychotherapy:exercise (Balance Group) | 0 | No concerns | Low risk | No concerns | Major concerns | No concerns | No concerns | Low |
| cognitive intervention+psychotherapy:psychotherapy | 0 | No concerns | Low risk | No concerns | Major concerns | No concerns | No concerns | Low |
| cognitive intervention+psychotherapy:psychotherapy+exercise | 0 | No concerns | Low risk | No concerns | Major concerns | No concerns | No concerns | Low |
| cognitive intervention+psychotherapy+exercise:exercise (Balance Group) | 0 | No concerns | Low risk | No concerns | Major concerns | No concerns | No concerns | Low |
| cognitive intervention+psychotherapy+exercise:psychotherapy | 0 | No concerns | Low risk | No concerns | Major concerns | No concerns | No concerns | Low |
| cognitive intervention+psychotherapy+exercise:usual care | 0 | No concerns | Low risk | No concerns | Major concerns | No concerns | No concerns | Low |
| exercise (Balance Group):psychotherapy | 0 | No concerns | Low risk | No concerns | Major concerns | No concerns | No concerns | Low |
| exercise (Balance Group):psychotherapy+exercise | 0 | No concerns | Low risk | No concerns | Major concerns | No concerns | No concerns | Low |
| exercise (Balance Group):usual care | 0 | No concerns | Low risk | No concerns | Major concerns | No concerns | No concerns | Low |
| exercise (Balance Group):wl | 0 | No concerns | Low risk | No concerns | Some concerns | Some concerns | No concerns | Low |
| psychotherapy:psychotherapy+exercise | 0 | No concerns | Low risk | No concerns | Major concerns | No concerns | No concerns | Low |
| psychotherapy:wl | 0 | No concerns | Low risk | No concerns | Major concerns | No concerns | No concerns | Low |
| psychotherapy+exercise:usual care | 0 | No concerns | Low risk | No concerns | Major concerns | No concerns | No concerns | Low |
| usual care:wl | 0 | No concerns | Low risk | No concerns | Major concerns | No concerns | No concerns | Low |

**12.4.8 Depression**

| **Comparison** | **N. of studies** | **Within-study bias** | **Reporting bias** | **Indirectness** | **Imprecision** | **Heterogeneity** | **Incoherence** | **Confidence**  **rating** |
| --- | --- | --- | --- | --- | --- | --- | --- | --- |
| active placebo:cognitive intervention | 1 | Some concerns | Low risk | No concerns | Major concerns | No concerns | No concerns | Very Low |
| active placebo:wl | 1 | Some concerns | Low risk | No concerns | Major concerns | No concerns | No concerns | Very Low |
| cognitive intervention:cognitive intervention+occupational therapy | 1 | No concerns | Low risk | No concerns | Major concerns | No concerns | No concerns | Low |
| cognitive intervention:cognitive intervention+psychotherapy | 1 | No concerns | Low risk | No concerns | Major concerns | No concerns | No concerns | Low |
| cognitive intervention:exercise (Balance Group) | 1 | No concerns | Low risk | No concerns | Major concerns | No concerns | No concerns | Low |
| cognitive intervention:usual care | 1 | No concerns | Low risk | No concerns | Some concerns | Some concerns | No concerns | Low |
| cognitive intervention:wl | 2 | Some concerns | Low risk | No concerns | Some concerns | Some concerns | No concerns | Very Low |
| cognitive intervention+psychotherapy:usual care | 1 | No concerns | Low risk | No concerns | Some concerns | Some concerns | No concerns | Low |
| cognitive intervention+psychotherapy+exercise:psychotherapy+exercise | 1 | No concerns | Low risk | No concerns | Major concerns | No concerns | No concerns | Low |
| cognitive intervention+psychotherapy+exercise:wl | 2 | No concerns | Low risk | No concerns | Some concerns | Some concerns | No concerns | Low |
| psychotherapy therapy:usual care | 1 | No concerns | Low risk | No concerns | Some concerns | Some concerns | No concerns | Low |
| psychotherapy+exercise:wl | 1 | No concerns | Low risk | No concerns | Major concerns | No concerns | No concerns | Low |
| active placebo:cognitive intervention+occupational therapy | 0 | Some concerns | Low risk | No concerns | Major concerns | No concerns | No concerns | Very Low |
| active placebo:cognitive intervention+psychotherapy | 0 | Some concerns | Low risk | No concerns | Major concerns | No concerns | No concerns | Very Low |
| active placebo:cognitive intervention+psychotherapy+exercise | 0 | Some concerns | Low risk | No concerns | Major concerns | No concerns | No concerns | Very Low |
| active placebo:exercise (Balance Group) | 0 | Some concerns | Low risk | No concerns | Major concerns | No concerns | No concerns | Very Low |
| active placebo:psychotherapy therapy | 0 | No concerns | Low risk | No concerns | Some concerns | Some concerns | No concerns | Low |
| active placebo:psychotherapy+exercise | 0 | No concerns | Low risk | No concerns | Major concerns | No concerns | No concerns | Low |
| active placebo:usual care | 0 | Some concerns | Low risk | No concerns | Some concerns | Some concerns | No concerns | Very Low |
| cognitive intervention:cognitive intervention+psychotherapy+exercise | 0 | Some concerns | Low risk | No concerns | Major concerns | No concerns | No concerns | Very Low |
| cognitive intervention:psychotherapy therapy | 0 | No concerns | Low risk | No concerns | Some concerns | Some concerns | No concerns | Low |
| cognitive intervention:psychotherapy+exercise | 0 | No concerns | Low risk | No concerns | Major concerns | No concerns | No concerns | Low |
| cognitive intervention+occupational therapy:cognitive intervention+psychotherapy | 0 | No concerns | Low risk | No concerns | Major concerns | No concerns | No concerns | Low |
| cognitive intervention+occupational therapy:cognitive intervention+psychotherapy+exercise | 0 | No concerns | Low risk | No concerns | Major concerns | No concerns | No concerns | Low |
| cognitive intervention+occupational therapy:exercise (Balance Group) | 0 | No concerns | Low risk | No concerns | Major concerns | No concerns | No concerns | Low |
| cognitive intervention+occupational therapy:psychotherapy therapy | 0 | No concerns | Low risk | No concerns | Major concerns | No concerns | No concerns | Low |
| cognitive intervention+occupational therapy:psychotherapy+exercise | 0 | No concerns | Low risk | No concerns | Major concerns | No concerns | No concerns | Low |
| cognitive intervention+occupational therapy:usual care | 0 | No concerns | Low risk | No concerns | Major concerns | No concerns | No concerns | Low |
| cognitive intervention+occupational therapy:wl | 0 | Some concerns | Low risk | No concerns | Major concerns | No concerns | No concerns | Very Low |
| cognitive intervention+psychotherapy:cognitive intervention+psychotherapy+exercise | 0 | No concerns | Low risk | No concerns | Major concerns | No concerns | No concerns | Low |
| cognitive intervention+psychotherapy:exercise (Balance Group) | 0 | No concerns | Low risk | No concerns | Major concerns | No concerns | No concerns | Low |
| cognitive intervention+psychotherapy:psychotherapy therapy | 0 | No concerns | Low risk | No concerns | Some concerns | Some concerns | No concerns | Low |
| cognitive intervention+psychotherapy:psychotherapy+exercise | 0 | No concerns | Low risk | No concerns | Major concerns | No concerns | No concerns | Low |
| cognitive intervention+psychotherapy:wl | 0 | No concerns | Low risk | No concerns | Major concerns | No concerns | No concerns | Low |
| cognitive intervention+psychotherapy+exercise:exercise (Balance Group) | 0 | No concerns | Low risk | No concerns | Major concerns | No concerns | No concerns | Low |
| cognitive intervention+psychotherapy+exercise:psychotherapy therapy | 0 | No concerns | Low risk | No concerns | Some concerns | Some concerns | No concerns | Low |
| cognitive intervention+psychotherapy+exercise:usual care | 0 | No concerns | Low risk | No concerns | Some concerns | Some concerns | No concerns | Low |
| exercise (Balance Group):psychotherapy therapy | 0 | No concerns | Low risk | No concerns | Major concerns | No concerns | No concerns | Low |
| exercise (Balance Group):psychotherapy+exercise | 0 | No concerns | Low risk | No concerns | Major concerns | No concerns | No concerns | Low |
| exercise (Balance Group):usual care | 0 | No concerns | Low risk | No concerns | Major concerns | No concerns | No concerns | Low |
| exercise (Balance Group):wl | 0 | Some concerns | Low risk | No concerns | Major concerns | No concerns | No concerns | Very Low |
| psychotherapy+exercise:psychotherapy therapy | 0 | No concerns | Low risk | No concerns | Some concerns | Some concerns | No concerns | Low |
| psychotherapy therapy:wl | 0 | No concerns | Low risk | No concerns | Some concerns | Some concerns | No concerns | Low |
| psychotherapy+exercise:usual care | 0 | No concerns | Low risk | No concerns | Some concerns | Some concerns | No concerns | Low |
| usual care:wl | 0 | No concerns | Low risk | No concerns | Some concerns | Some concerns | No concerns | Low |

# eAppendix 13. Sensitivity analyses

**13.1 Results of** **leave-one-out**

**13.1.1 Subjective memory complaints—cognitive intervention vs usual care**


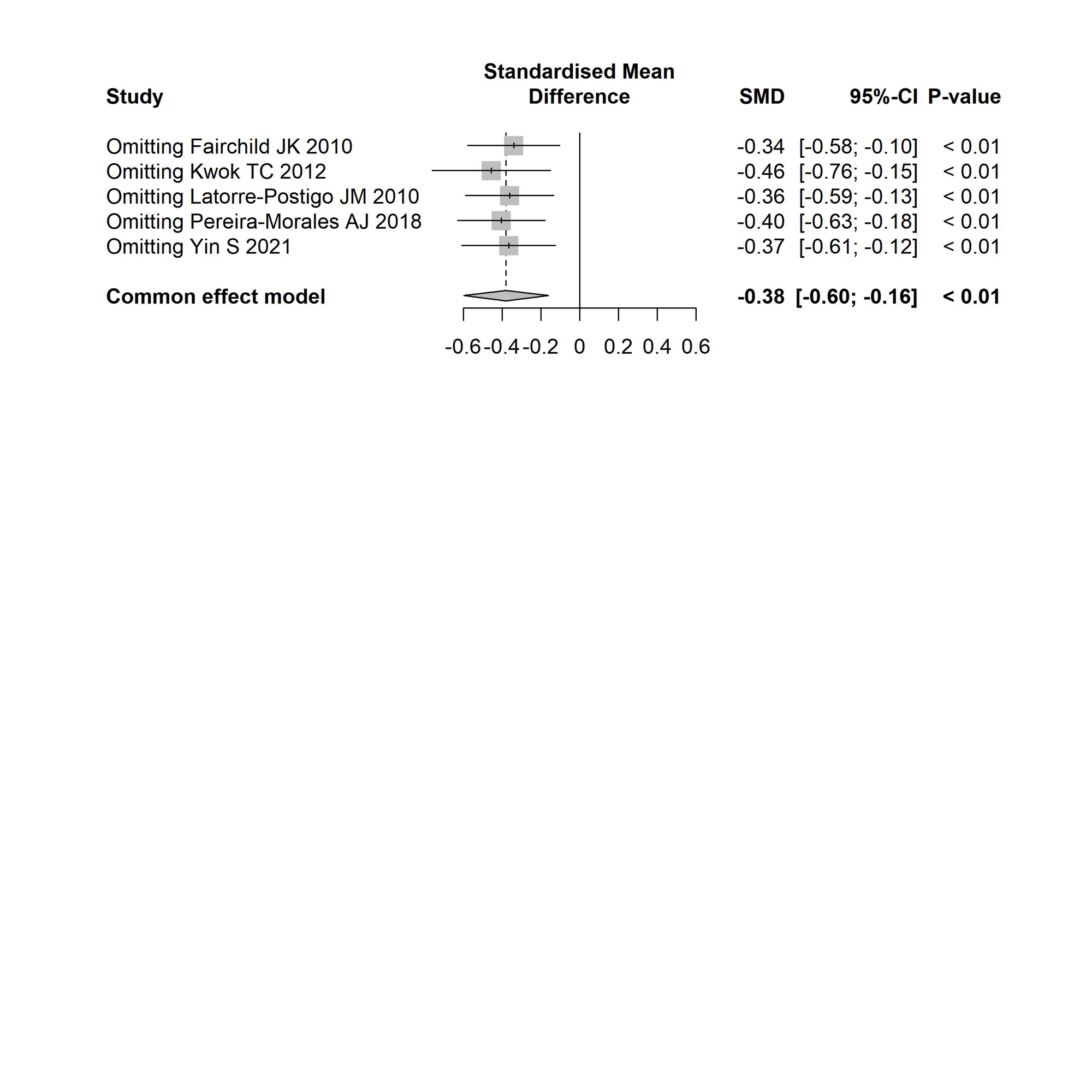


**13.1.2 Subjective memory complaints—cognitive intervention vs waitlist**


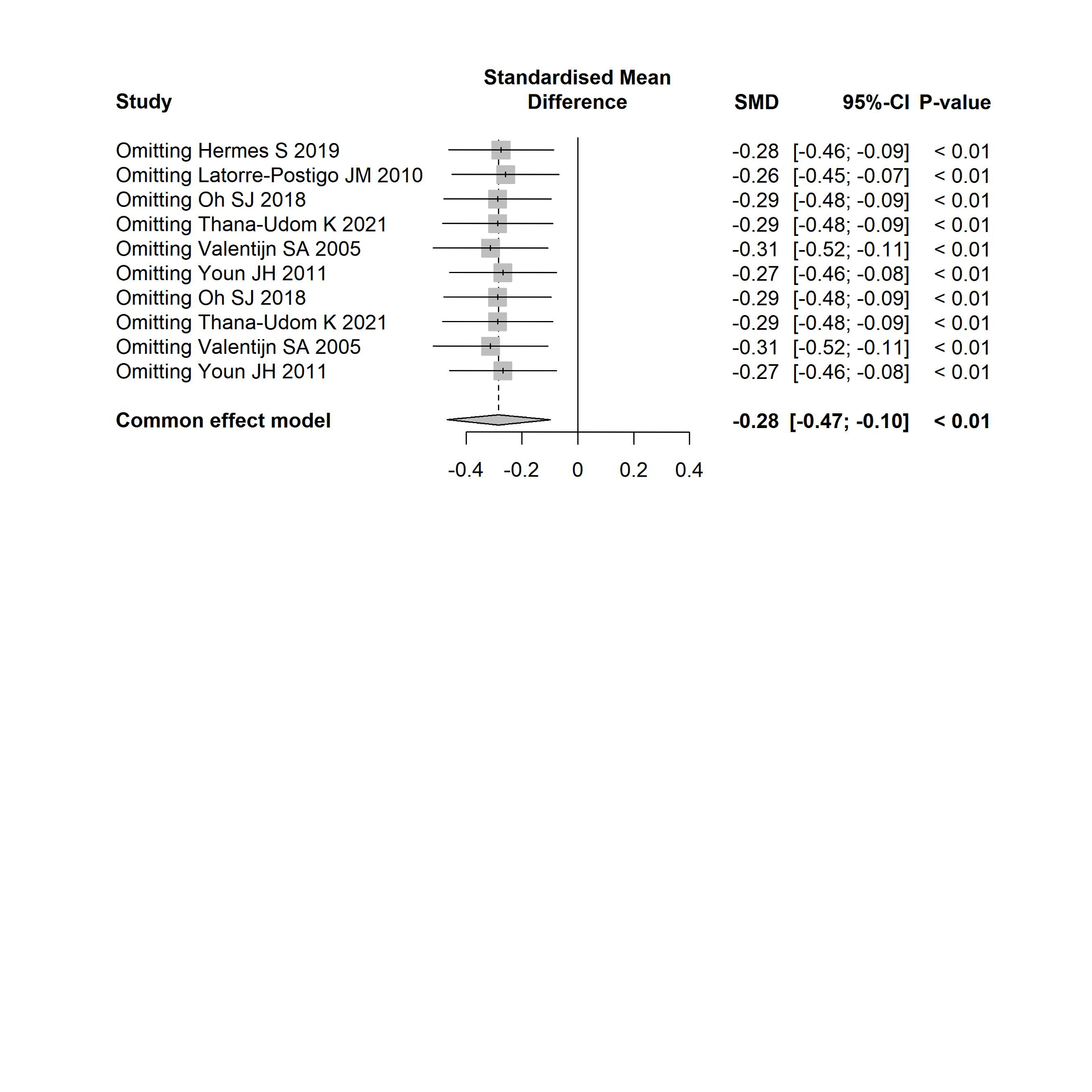


**13.2 Results of exclusion of high-risk of bias studies**

In the primary outcome (subjective memory complaints), only the Kwok TC et al. (2012) study was rated as high risk. Therefore, sensitivity analyses were performed on models that included cognitive intervention vs usual care, and Kwok TC (2012) was excluded. This result is consistent with our previous sensitivity analysis using the leave-one-out method.

# eAppendix 14. Subgroup analyses

**14.1 Subjective memory complaints—NPIs vs usual care**


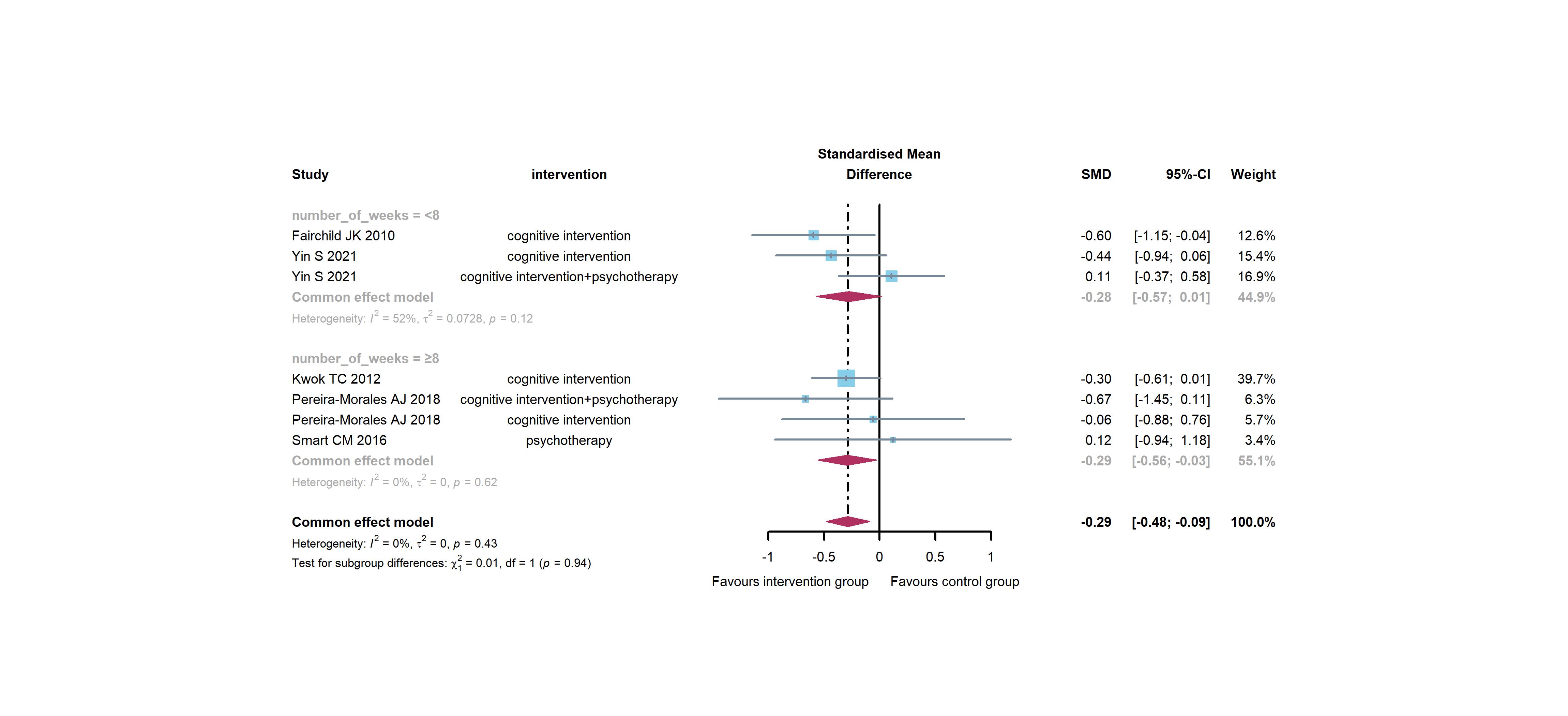


**14.1 Subjective memory complaints—NPIs vs waitlist**

**
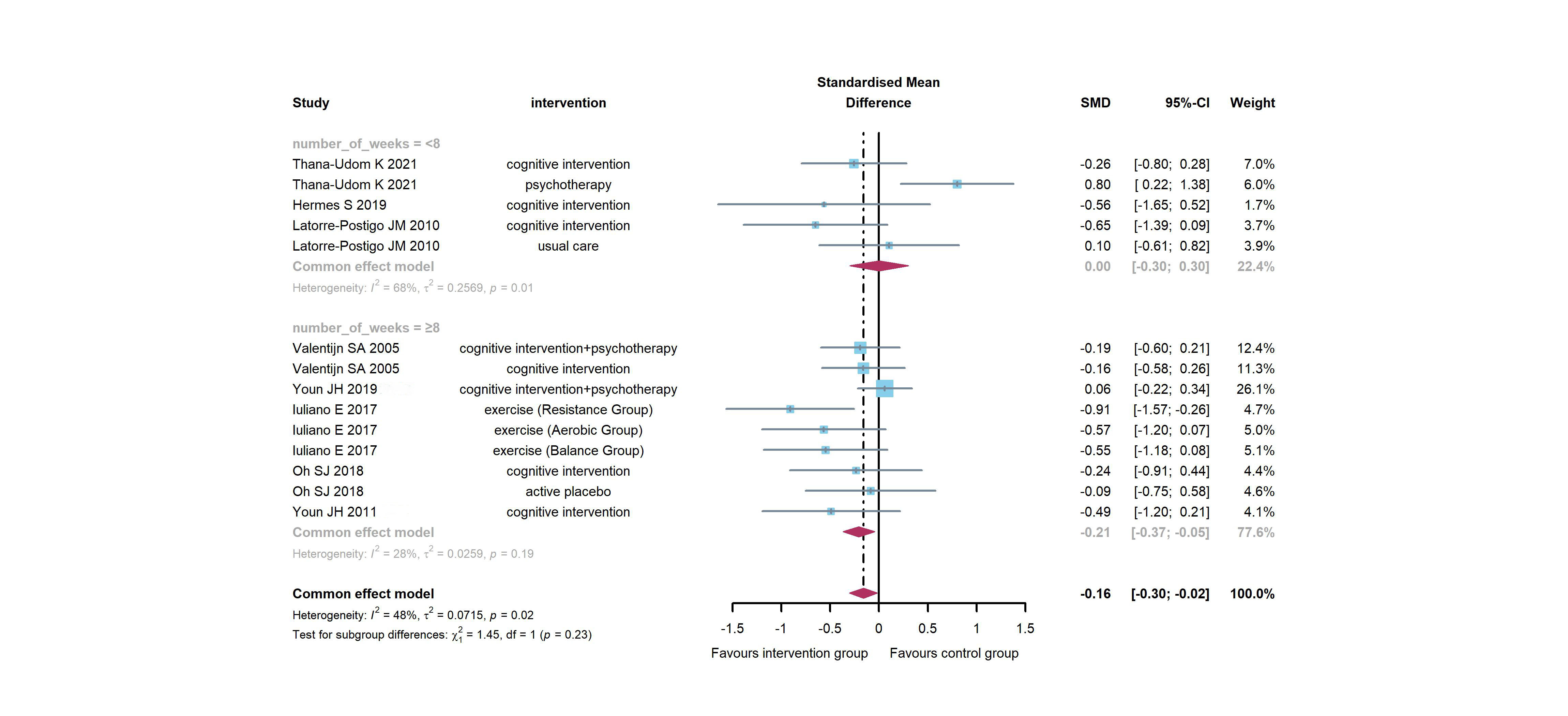
**
